# Metabolic Labeling of Legionaminic Acid in Flagellin Glycosylation of *Campylobacter jejuni* Identifies Maf4 as a Putative Legionaminyl Transferase

**DOI:** 10.1002/anie.202107181

**Published:** 2021-10-27

**Authors:** Xianke Meng, Geert‐Jan Boons, Marc M. S. M. Wösten, Tom Wennekes

**Affiliations:** ^1^ Department of Chemical Biology and Drug Discovery Utrecht Institute for Pharmaceutical Sciences and Bijvoet Center for Biomolecular Research Utrecht University Universiteitsweg 99 3584 CG Utrecht The Netherlands; ^2^ Complex Carbohydrate Research Center and Department of Chemistry University of Georgia 315 Riverbend Road Athens GA 30602 USA; ^3^ Department Biomolecular Health Sciences Utrecht University Yalelaan 1 3584 CL Utrecht The Netherlands

**Keywords:** bacterial flagella, carbohydrates, glycoproteins, glycosyltransferase, metabolic labeling

## Abstract

*Campylobacter jejuni* is the major human food‐borne pathogen. Its bipolar flagella are heavily *O*‐glycosylated with microbial sialic acids and essential for its motility and pathogenicity. However, both the glycosylation of flagella and the exact contribution of legionaminic acid (Leg) to flagellar activity is poorly understood. Herein, we report the development of a metabolic labeling method for Leg glycosylation on bacterial flagella with probes based on azide‐modified Leg precursors. The hereby azido‐Leg labeled flagellin could be detected by Western blot analysis and imaged on intact bacteria. Using the probes on *C. jejuni* and its isogenic *maf4* mutant we also further substantiated the identification of Maf4 as a putative Leg glycosyltransferase. Further evidence was provided by UPLC–MS detection of labeled CMP‐Leg and an in silico model of Maf4. This method and the developed probes will facilitate the study of Leg glycosylation and the functional role of this modification in *C. jejuni* motility and invasiveness.

## Graphical abstract

Probes used to metabolically label legionaminic acid on bacterial flagella substantiated the identification of Maf4 as a putative legionaminyl transferase in *Campylobacter jejuni* (see picture). The labeled glycan could be detected intracellularly as its activated CMP analogue, on flagella by Western blot analysis, and on intact bacteria by confocal microscopy.

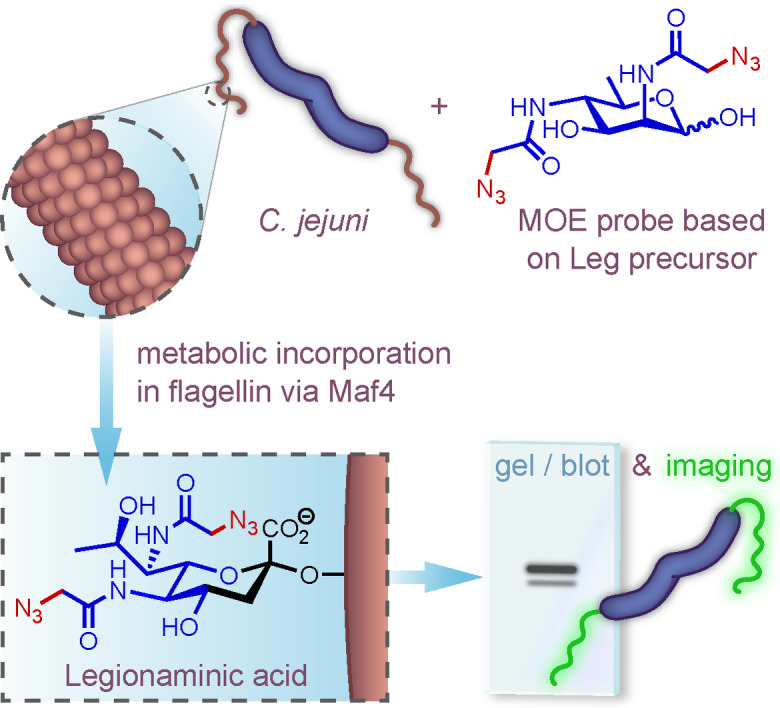

*Campylobacter jejuni* is the leading cause of bacterial gastroenteritis worldwide. It is a highly motile bacterium due to its bipolar flagella that play an important role in host colonization and infections of humans.^[1]^ The flagellar filament of *C. jejuni* is composed of two highly homologous flagellin proteins, FlaA and FlaB. The flagellins of *C. jejuni* are extensively *O*‐glycosylated with microbial nonulosonic acids such as pseudaminic acid (Pse) and legionaminic acid (Leg) (Figure 1).^[2]^ It has been shown that *O*‐glycosylation with Pse is required for proper filament formation in *C. jejuni* and thus for a functional flagella.^[3]^ Glycosylation of flagellin proteins with Leg derivatives correlates with an ability to form biofilms and auto‐agglutinate, the latter activity is often a marker for virulence and interaction with host cells in Gram‐negative bacterial pathogens. However, the functional role Leg plays in these processes and in flagella functioning is largely unknown.[^2^, ^4^]

**Figure 1:**
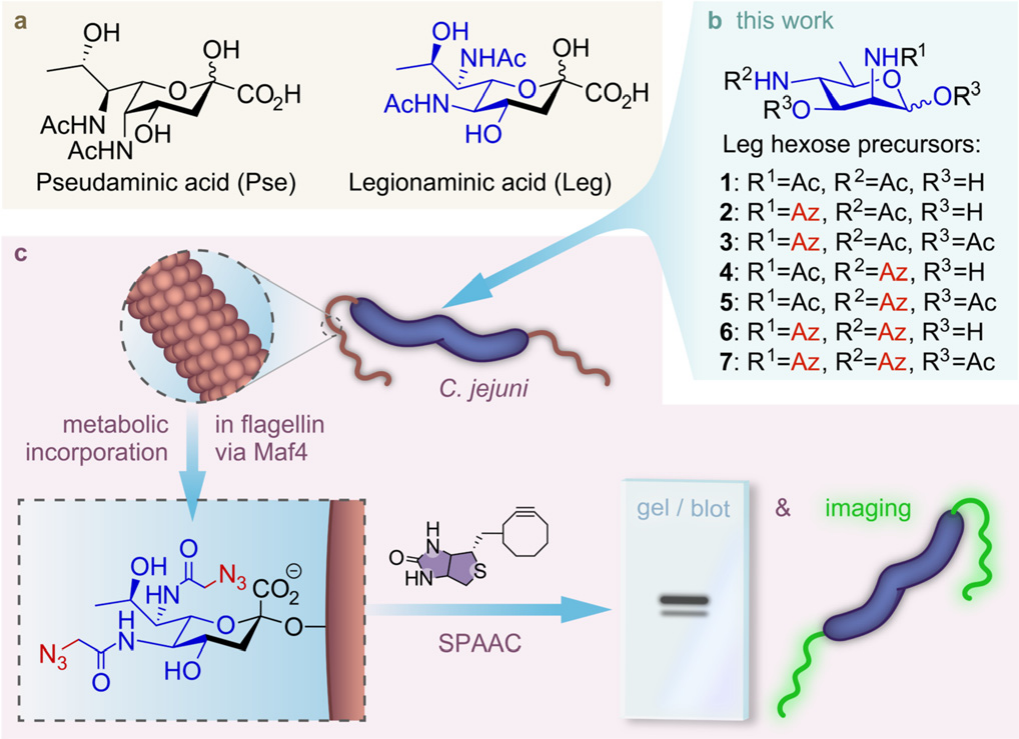
a) Structures of Pse and Leg. b) Structures of native Leg precursor **1**, and Leg precursor analogues **2**–**7** developed for this study. c) Depiction of workflow for metabolic labeling and analysis of labeled Leg in flagellin glycosylation of *C. jejuni*. Az=azidoacetyl.

In 1994, legionaminic acid was identified by Knirel and co‐workers as a component of the lipopolysaccharide (LPS) of *Legionella pneumophila*, the causative agent of Legionnaires’ disease.^[5]^ Subsequently, legionaminic acid and derivatives thereof have been found in numerous bacterial species, including, *Campylobacter*, *Pseudomonas*, *Vibrio*, *Acinetobacter*, *Escherichia* and *Salmonella*.^[6]^

The biosynthetic pathway of CMP‐legionaminic acid (CMP‐Leg) has been elucidated in *C. jejuni*.^[7]^ It involves six‐enzymes and starts with the conversion of GDP‐GlcNAc into **8** by LegB, a NAD^+^‐dependent dehydratase (Scheme 1). The second enzyme is a PLP‐dependent aminotransferase, LegC, that installs an amino group at C4 to produce **9**. The latter intermediate is transformed into **10** by the action of the acetyltransferase LegH. A hydrolase and 2‐epimerase, LegG, converts **10** into the key non‐phosphorylated hexose precursor **1** that is condensed with phosphoenolpyruvate (PEP) by legionaminic acid synthase, LegI, to produce Leg. Finally, Leg is activated by the cytidyltransferase LegF as CMP‐Leg for glycoconjugate synthesis mediated by a glycosyltransferase that has yet to be identified.

**Scheme 1:**
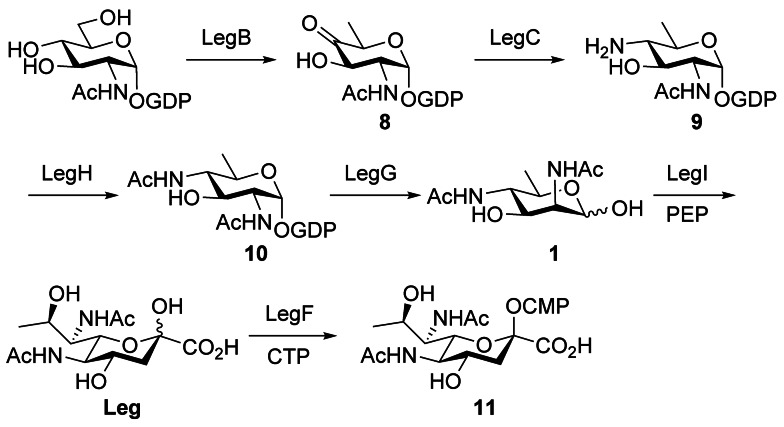
Legionaminic acid biosynthetic pathway in *C. jejuni*.

The development of a versatile molecular toolbox of Leg‐based probes represents a key approach to illuminate the presence and functional role of Leg glycosylation in bacteria and identify the involved enzymatic activities. We herein report the design and synthesis of six probes (**2**–**7**) with azide reporters based on Leg hexose precursor (**1**). We evaluated their ability for metabolic oligosaccharide engineering (MOE) of Leg glycosylation in *C. jejuni* (Figure 1) and establish that three of these probes (**2**, **4** and **6**) could be incorporated into the flagella of *C. jejuni* strains through their legionaminic acid biosynthetic pathway. Furthermore, by using probe **6** we identified that the Maf4 protein of *C. jejuni* may be a putative Leg glycosyltransferase, as this probe could successfully be converted into its CMP‐Leg analogue, but its incorporation into the flagellins of the *maf4* mutant was strongly diminished.

MOE is a powerful approach to explore and visualize cell‐surface glycoconjugates, also in bacteria.^[8]^ It has been successfully applied for the detection of Pse glycosylation on bacteria, such as *C. jejuni* and *Pseudomonas aeruginosa*.^[9]^ For legionaminic acid it is known that a C6 azide modification of hexose precursor **1** allows for species‐specific labeling by MOE of *Legionella pneumophilia*.^[10]^ For this study, we also selected precursor **1** as a suitable target for MOE as we judged that it could be readily synthetized with bioorthogonal chemical reporters and its lack of a charge would hopefully allow for sufficient uptake by *C. jejuni*. As it was not yet known if one or both of the *N*‐acyl positions of **1** could be used for modification towards a MOE probe, we planned to install an azidoacetamide group on either C2 and C4 or both positions of **1**. Additionally, the hydroxyls on these modified precursors could be acetylated to potentially facilitate the passive diffusion into bacterial cells. *In situ* deacetylation and further processing by the downstream Leg biosynthesis enzymes would then also lead to labeling.^[11]^

Commercially available d‐fucose **12** was chosen as the starting material based on the ability to simultaneously substitute with inversion at the C2 and C4 position, as reported by Chen (Scheme 2).^[12]^ After full acetylation of **12**, the *p*‐methoxyphenyl group was installed onto the anomeric center in the presence of BF_3_⋅Et_2_O to afford **13**. Deacetylation of **13** using sodium methoxide and aminoethyl diphenylborinate‐mediated regioselective O3‐benzoylation afforded **14** in 73 % yield in two steps.^[13]^ Conversion of **14** into the corresponding 2,4‐bistriflate, followed by bis‐azidation with tetrabutylammonium azide through S_N_2 substitution, resulted in diazide **15** with the d‐*manno* configuration in 84 % yield over two steps. The benzoate group was removed using sodium methoxide in methanol to obtain **16** in 95 % yield. This step proved necessary as migration of the benzoate group was otherwise observed during reduction of the azide in its presence. Reduction of the 2,4‐azido groups was performed by hydrogenation over Pd(OH)_2_/C and followed by N‐acetylation to give **20** in 58 % yield over 2 steps. To synthesize **19** with di‐*N*‐azidoacetyl moieties, the amines that formed after reduction of azido groups, were directly coupled with azidoacetic acid through an EDC‐mediated coupling reaction. Interestingly, next to the desired di‐*N*‐azidoacetyl product **19** another more polar product **S1** also formed and was identified as having only a N2‐azidoacetyl modification (Scheme S1). This reactivity difference between the amine at C2 and C4 could be used to our advantage by subsequently converting **S1** into **17** by N4‐acetylation. For the synthesis of **18**, the reactivity difference was again used to install an acetyl group onto N2 selectively. Subsequent N4‐azidoacetyl modification and treatment with sodium methoxide provided **18**. Removal of the methoxyphenyl group of intermediates **17**–**20** by ceric ammonium nitrate gave the target chemical probes **2**, **4**, **6** and native precursor **1** respectively. Finally, acetylation of **2**, **4**, **6** afforded the corresponding *O*‐acetylated versions **3**, **5**, **7** of the probes in good yield.

**Scheme 2:**
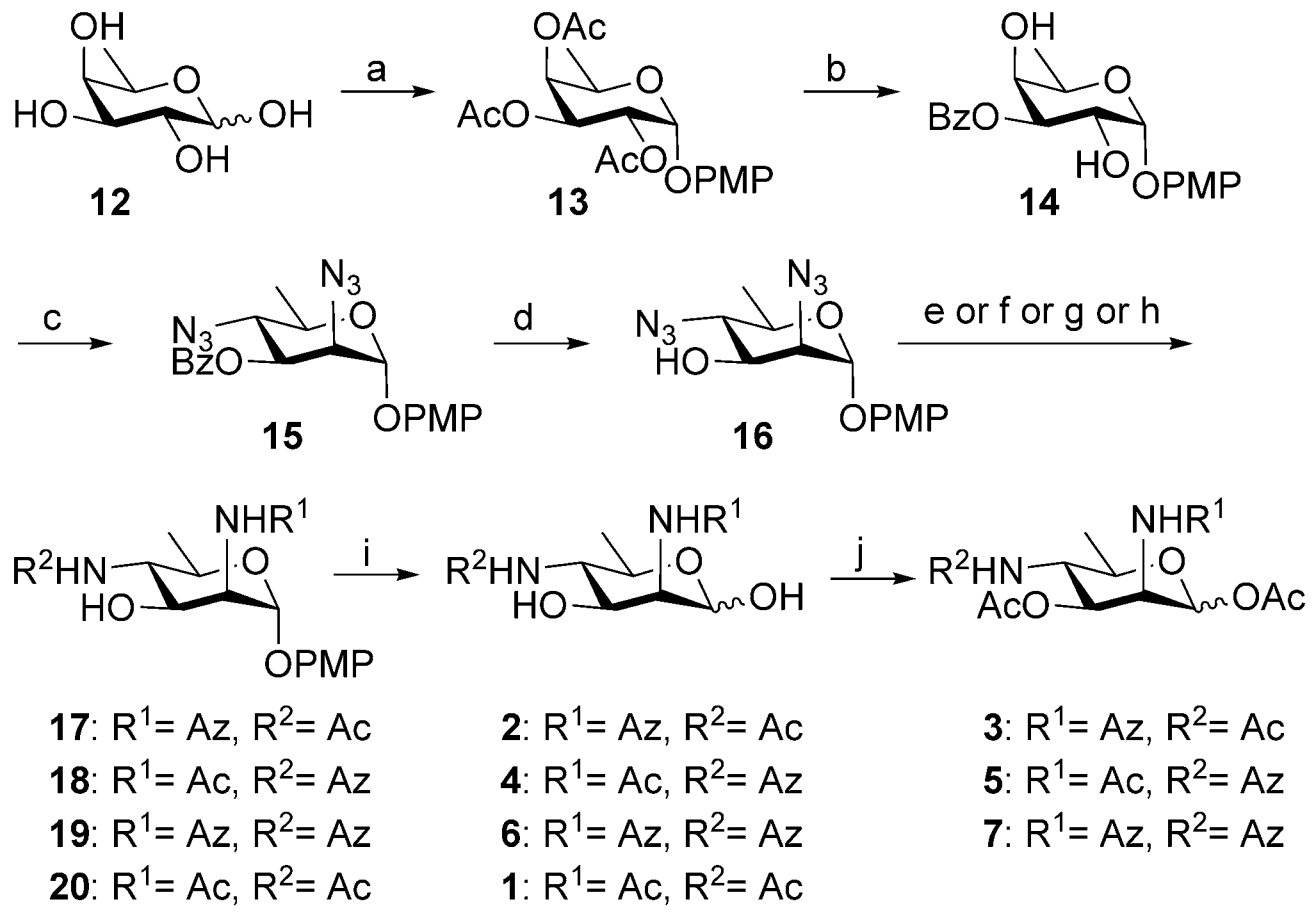
Synthesis of Leg precursor **1** and its azido analogues **2**–**7**. Reagents and conditions: a) i) Ac_2_O, pyridine, 0 °C→rt; ii) PMPOH, BF_3_⋅Et_2_O, CH_2_Cl_2_, 0 °C→rt, 83 % over two steps; b) i) NaOMe, MeOH, reflux; ii) 2‐aminoethyl diphenylborinate, DIPEA, BzCl, CH_3_CN, 74 % over two steps; c) i) Tf_2_O, pyridine, DCM, −10 °C; ii) TBAN_3_, toluene, 70 °C for 1 h, then 100 °C for 1 h, 84 % over two steps; d) NaOMe, MeOH, reflux, 95 %; e) for **17**: i) Pd(OH)_2_/C, H_2_, MeOH; ii) EDC, HOBT, NaHCO_3_, AzOH, CH_3_CN; iii) Ac_2_O, MeOH, 0 °C, 40 % over three steps; f) for **18**: i) Pd(OH)_2_/C, H_2_, MeOH; ii) Ac_2_O, MeOH, 0 °C; iii) EDC, HOBT, NaHCO_3_, AzOH, CH_3_CN; iv) NaOMe, MeOH, 36 % over four steps; g) for **19**: i) Pd(OH)_2_/C, H_2_, MeOH; ii) EDC, HOBT, NaHCO_3_, AzOH, CH_3_CN; iii) NaOMe, MeOH, 55 % over three steps; h) for **20**: i) Pd(OH)_2_/C, H_2_, MeOH; ii) Ac_2_O, MeOH, 58 % over two steps; i) CAN, CH_3_CN/H_2_O (3:1 v/v), 71 % for **2**, 72 % for **4**, 60 % for **6**, 75 % for **1**; j) Ac_2_O, Et_3_N, CH_2_Cl_2_, 81 % for **3**, 85 % for **5**, 82 % for **7**. Bz=benzoyl, CAN=ceric ammonium nitrate, DCM=dichloromethane, DIPEA=*N*,*N*‐diisopropylethylamine, EDC=1‐ethyl‐3‐(3‐dimethylaminopropyl)carbodiimide hydrochloride, HOBT=1‐hydroxy‐1*H*‐benzotriazole, Tf=trifluoromethanesulfonate.

We first set out to screen the set of developed probes **2**–**7** for their ability to be salvaged and incorporated by *C. jejuni* as legionaminic acid into its glycans. *C. jejuni* 11 168 was cultured for 4 h in the presence of 1 mM of probes **2**–**7** and afterwards any incorporated Leg probe was labeled through a strain‐promoted azide–alkyne cycloaddition (SPAAC) reaction with DBCO (dibenzocyclooctyne)‐PEG_4_‐biotin. The resulting biotinylated glycoconjugates in whole cell lysates were detected on Western blot using Streptavidin‐HRP. The only biotinylated proteins from the bacterial cells that were detected originated from experiments with the non‐acetylated probes (**2**, **4**, **6**). Treatment with these probes only produced a single labeled band at approximately 70 kDa (Figure 2 a), which is the approximate size of *C. jejuni* flagellins. To confirm that these streptavidin‐reactive bands indeed belonged to flagellin, the whole‐cell lysates were treated with an anti‐flagellin antibody and the hereby labeled bands all proved to have an identical migration patterns to the streptavidin‐reactive bands (Figure 2 a).^[14]^ Further inspection indicated that probe **6**, containing two azido groups, shows the best labeling result compared to probe **2** or **4** (relative band intensity for **2** and **4** was, respectively 36 % and 30 % lower compared to **6**), which only contain a single azido group at C2 or C4 position. With the acetylated versions of these probes (**3**, **5**, **7**) we wanted to test whether labeling could also be achieved by entry into the bacteria by passive transport, and intracellular conversion into probes **2**, **4** and **6** by nonspecific bacterial esterases. No labeling was however observed with these three probes, suggesting that there is insufficient esterase activity inside *C. jejuni* to remove the acetyl group.[^10^, ^15^]

**Figure 2:**
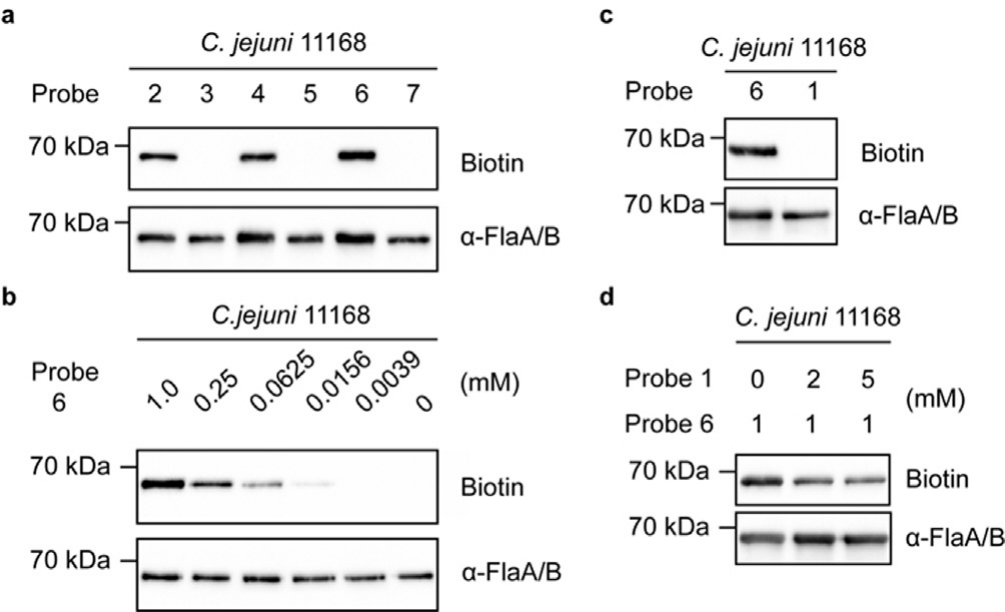
Western blot analysis of *C. jejuni* 11168 flagellins probed with Leg hexose precursor analogues. a) Incorporation of probes **2**–**7** into the *C. jejuni* 11168 flagellin. *C. jejuni* 11168 cells were grown in HI medium supplemented with 1 mM probe **2**–**7** for 4 h. Next the bacteria were incubated with 10 μM DBCO‐PEG_4_‐biotin for 1 h in PBS. Cells were lysed and biotin detection was performed by streptavidin blotting. b) Concentration‐dependent labeling of probe **6** at various concentrations. c) Comparison of labeling between probe **6** and 6‐deoxyManNAc4NAc **1**. d) Competition experiment between probe **6** (1 mM) and 6‐deoxyManNAc4NAc **1** with increasing concentration. The anti‐FlaA/FlaB antibody was used to detect the amount of flagellins in each sample.

Next, a concentration‐dependent labeling experiment was performed with probe **6**, ranging from 0 to 1.0 mM (Figure 2 b). The use of probe **6** as low as 16 μM still resulted in detectable labeling of the flagellar proteins and there was a positive correlation between the signal intensity and the probe concentration. The optimal incorporation of probe **6** was however observed when bacteria were grown with 1 mM of **6** for 4 h at 42 °C. To verify whether probe **6** was being incorporated through the native Leg biosynthetic pathway, we conducted a competition experiment with native *N*‐acetylated Leg precursor **1**. First, bacteria cells were cultured with **1** and Western blot analysis indicated as expected that the natural precursor **1** did not result in detectable labeling of the flagellin (Figure 2 c). Then a competition assay between **1** and **6** was conducted by culturing the bacteria with both substrates simultaneously in different ratios (Figure 2 d). The detectable signal from incorporated azido‐labeled Leg decreased when **1** was present in twofold excess. This result indicates that probe **6** is competing with native precursor **1** and thus plausibly being incorporated through the same metabolic pathway. Interestingly, the signal originating from incorporation of **6** could still be detected after adding fivefold excess of **1**, which indicates that **6** might even be a better substrate for one or several of the enzymes involved. To further confirm that probe **6** is converted into the nucleotide–sugar by the bacterial biosynthetic machinery, we sought to detect the existence of cellular CMP‐LegdiNAz. Bacterial cells treated with 1 mM probe **6** for 4 h were lysed, extracted and analyzed by UPLC–MS. For CMP‐LegdiNAz, [M−H]^−^ is calculated as *m*/*z* 720.1744, and it was indeed detected as *m*/*z* 720.1728 (2.2 ppm) in the sample treated with **6** while such signal was not detected in the sample untreated with **6**, suggesting that the unnatural nucleotide sugar was indeed produced (Figure 3, S2).

**Figure 3:**
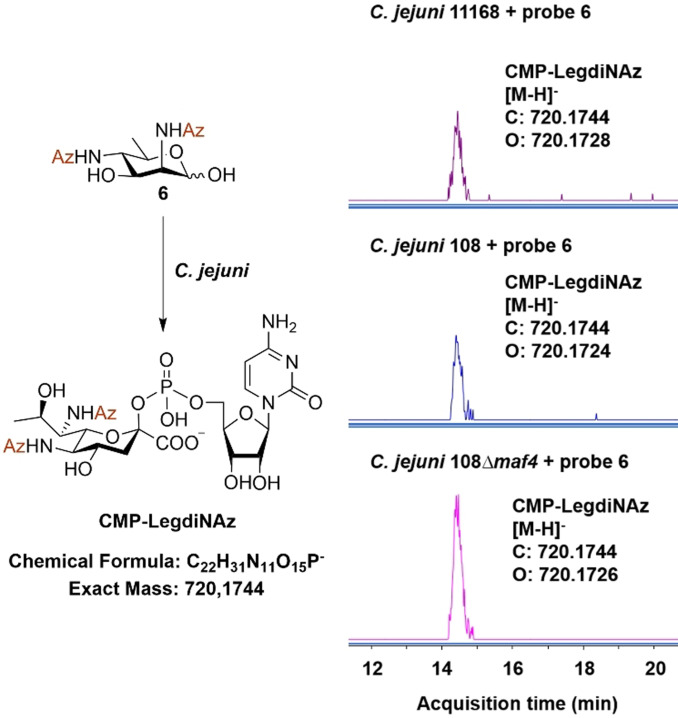
UPLC–MS extracted‐ion chromatogram (EIC) analysis of CMP‐LegdiNAz from *C. jejuni* 11168, *C. jejuni* 108 and *C. jejuni* 108 *Δmaf4* cells treated with 1 mM probe **6**.

Encouraged by these results, we applied the developed labeling strategy with probe **6** on two other *C. jejuni* strains, 129108 (abbreviated as 108) and 81116. Genomic analysis shows that *C. jejuni* 108 has the genes that encode for the enzymes responsible for the Leg biosynthetic pathway, while *C. jejuni* 81116 lacks this pathway.^[16]^ Both strains were grown in HI media supplemented with probe **6** for 7 h and as expected Western blot analysis revealed that the flagella of *C. jejuni* 108 could indeed be labeled, while those of strain 81116 were not labeled*. C. jejuni* 81116 ΔflaAB, a non‐flagellated mutant, was used as an additional negative control that as expected did not show any labeling with **6** (Figure S1). To gain insight into the degree of labeling, a band shift assay was performed with DBCO‐PEG5K on *C. jejuni* 11168 and 108 treated with **6**. This showed at least about 25 % of FlaA/B subunits were each labeled with a multiple copies of LegdiNAz (Figure S7). Finally, an autoagglutination analysis of both probe **6** treated and untreated *C. jejuni* 108 samples showed no difference in their rate of autoagglutination that tentatively indicates the azido modifications do not impact the functional role of Leg on the bacteria (Figure S5).

We next aimed to visualize incorporated azido‐labeled Leg on the flagella of intact bacterial cells. To achieve this, *C. jejuni* 11168 was grown in the presence of probe **6**, and the incorporation of the probe into flagella was detected through a SPAAC reaction with DBCO‐PEG_4_‐biotin. Any biotin labeling on the flagella was then visualized with Streptavidin‐Alexa Fluor 488 and CTY (CellTrace Yellow) was subsequently added to label the bacterial cell body. A distinct fluorescent signal was observed on the bacteria flagella by TIRF (total internal reflection fluorescent) microscopy (Figure 4).

**Figure 4:**
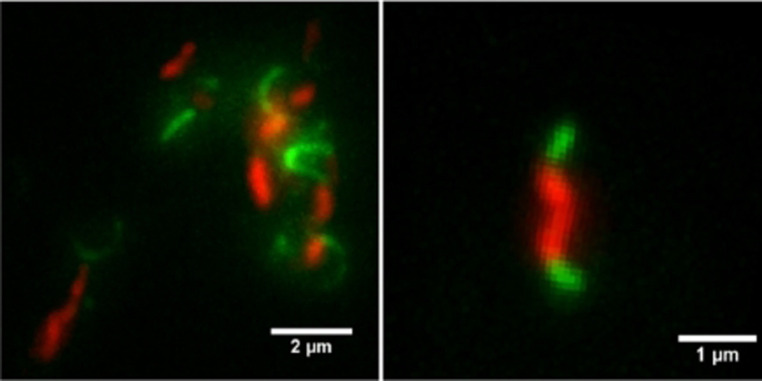
Live‐cell fluorescence labeling of *C. jejuni*. Bacteria treated with probe **6** for 4 h were incubated with DBCO‐PEG_4_‐biotin and stained with Streptavidin‐Alexa Fluor 488 (green). The cell body was stained with CTY (red). The bacteria were imaged by TIRF microscopy. Left: a labeled bacteria cluster; right: a single labeled bacterium.

The glycosyltransferase that uses CMP‐Leg to glycosylate the hydroxyl of Ser/Thr residues in the flagellin of *C. jejuni* has not been annotated yet so we next set out to use our optimal Leg MOE probe (**6**) to identify the involved bacterial glycosylation gene.[^17^, ^18^] Potential candidate genes encoding for it are members of the motility associated factors (*maf*) gene family.^[19]^ In *C. jejuni*, the *maf* genes are a part of the flagellar glycosylation locus that could influence flagella assembly and motility, but their exact functions remain unclear.^[20]^ A previous study by us proved that *maf4* in *C. jejuni* 108 is not required for flagellar assembly, but that it did affect flagellin glycosylation and altered flagellin glycosylation was observed in the mutant *C. jejuni* 108 *Δmaf4*. Analysis on 2D‐PAGE revealed that the flagellin proteins of strain 108 migrated at a more acidic pI than the protein of the *maf4* mutant.^[4]^ These results raised the possibility that *maf4* encodes for a glycosyltransferase that is capable of glycosylating flagellin with an acidic glycan, such as legionaminic acid. We first conducted a genomic analysis in 258 *C. jejuni* strains that indeed showed a very high correlation between the presence of *maf4* homologs and the genes to biosynthesize CMP‐Leg (Table S1). Incubation of probe **6** with two other representative strains from this analysis, *C. jejuni* RM1221 and 81‐176, verified that only the *maf4* and CMP‐Leg biosynthesis genes containing strain, RM1221, showed Leg labeling (Figure S8).

To further investigate the function of *maf4*, we incubated a *C. jejuni* 108 *Δmaf4* mutant with probe **6** and visualized labeled legionaminic acid glycosylation on the flagellin by Western blot. This resulted in only a very weak signal compared to the normal 108 strain (Figure 5). The minor residual labeling might be caused by another Leg transferase activity, for instance Maf1 (87 % amino acid sequence similarity to Maf4). Complementation of the *Δmaf4* mutant by a plasmid carrying an intact *maf4* restored the incorporation of the metabolized probe **6** into the Leg‐glycosylated flagellin. This result suggested Maf4 could be a putative legionaminic acid glycosyltransferase. We next used UPLC–MS again to determine the biosynthesis of the azido‐labeled Leg nucleotide–sugar for both strains when treated with probe **6**. This experiment showed CMP‐LegdiNAz in both strains (Figure 3, S2, S3) and interestingly the amount of CMP‐LegdiNAz in the *Δmaf4* mutant was found to be elevated compared to the wild type 108. This accumulation of CMP‐LegdiNAz might be explained by the lack of its consumption by the Leg glycosyltransferase, due to the knockout of the *maf4* gene.

**Figure 5:**
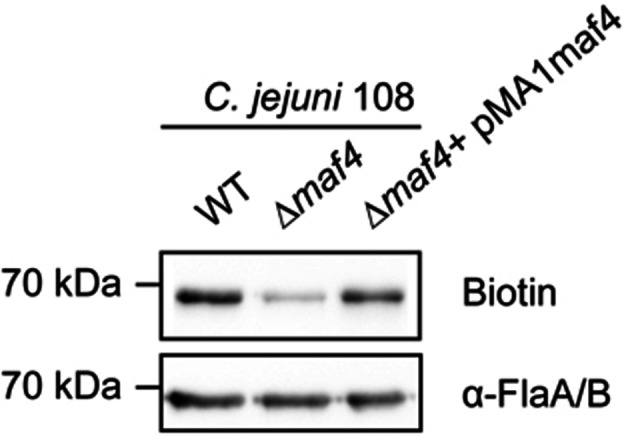
Western blot analysis of *C. jejuni* 108 (wild‐type, the *Δmaf4* mutant and the complemented *Δmaf4* mutant) probed with **6**. Bacteria cells were grown in HI medium supplemented with 1 mM probe **6** for 4 h, then incubated with 10 μM DBCO‐PEG_4_‐biotin for 1 h in PBS, and then lysed and further analyzed by streptavidin blotting. The anti‐FlaA/FlaB antibody was used to detect the amount of flagellins in each sample.

To further substantiate the role of Maf4 as a glycosyltransferase of microbial sialic acids, we used *in silico* analysis to build a model of the Maf4 protein (Figure 6) that was compared with the recently published crystal structure of the Maf enzyme of *M*. *magneticum* AMB‐1, a putative Pse glycosyltransferase.^[21]^ The hereby generated Maf4 model shares high similarity with Maf in the central α/β domain of the bacterial glycosyltransferase that was assigned as the substrate‐binding and catalysis region (Figure 6). A sequence alignment of this central α/β domain amino acid stretch for both Maf4 and Maf, together with other selected homologous bacterial proteins (Figure S6) that have been implicated with microbial sialic acid metabolism, showed many fully and highly conserved residues among which previously identified potential catalytic sites in Maf. Taken together, these results provide compelling evidence that Maf4 is a putative glycosyltransferase responsible for the transfer of Leg onto the flagellin in *C. jejuni*.

**Figure 6:**
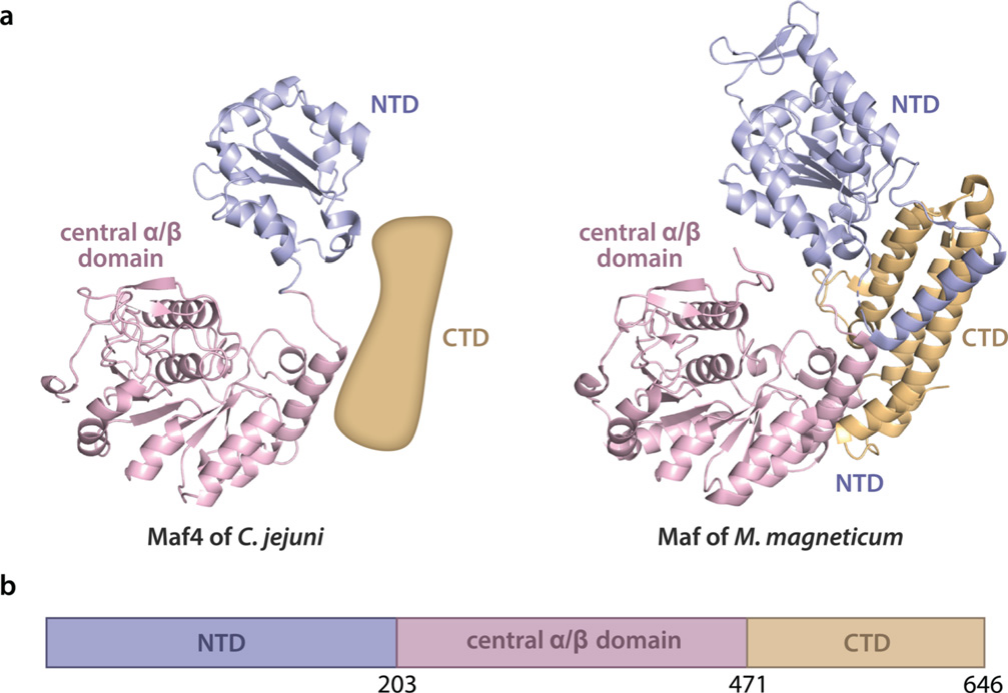
a) Comparison of the domain arrangement of the computational model of putative legionaminic acid transferase Maf4 from *C. jejuni* (from P88 to N473) with an approximated placeholder for unmodeled CTD domain due to limited similarity with the input model (left) and the crystal structure of putative pseudaminic acid transferase Maf of *Magnetospirillum magneticum* AMB‐1 (from I7 to D664; PDB entry 5MU5; right). b) Functional domain prediction of Maf4. Maf4 was comprised of the N‐terminal domain (residues 1–203), central α/β domain (residues 204–471), and C‐terminal domain (residues 472–646).

In summary, we have designed and synthesized probes based on the Leg hexose precursor (**1**) with azide reporters that were successfully used to study flagella glycosylation in *C. jejuni* strains using a MOE approach. This study shows that probes (**2**, **4**, **6**) based on **1** with non‐natural modifications on either N2 or N4 could be metabolically incorporated into bacterial flagella in specific strains, but not their *O*‐acetylated versions (**3**, **5**, **7**). With the azide reporters, the bacterial flagella could be selectively visualized by attaching a biotin and a fluorophore, respectively, thus providing the opportunity to study the location and abundance of Leg glycosylation. Moreover, the identity of Maf4, a putative Leg glycosyltransferase, was further substantiated using the developed probes and *in silico* modeling. We are currently further developing this approach as an efficient method for labeling *C. jejuni* flagella and to investigate the functional role of legionaminic acid in the dynamics of flagellin glycosylation and the filament assembly process in *C. jejuni*, also in relation to pseudaminic acid. An increased understanding of the role these microbial sialic acids have in bacterial motility and invasiveness of *C. jejuni* will aid in our ability to deal with this common human pathogen.

## Conflict of interest

The authors declare no conflict of interest.

## Supporting information

As a service to our authors and readers, this journal provides supporting information supplied by the authors. Such materials are peer reviewed and may be re‐organized for online delivery, but are not copy‐edited or typeset. Technical support issues arising from supporting information (other than missing files) should be addressed to the authors.

Supporting Information

Supporting Information
