# Supplementary material for: Metabolic Labeling of Legionaminic Acid in Flagellin Glycosylation of Campylobacter jejuni Identifies Maf4 as a Putative Legionaminyl Transferase
Source: Angew Chem Int Ed Engl. 2021 Oct 27;60(47):24811–6. doi: 10.1002/anie.202107181 (PMC9298399; doi:10.1002/anie.202107181)

Angewandte Chemie
Eine Zeitschrift der Gesellschaft Deutscher Chemiker

Supporting Information

Metabolic Labeling of Legionaminic Acid in Flagellin Glycosylation of *Campylobacter jejuni* Identifies Maf4 as a Putative Legionaminyl Transferase

Xianke Meng, Geert-Jan Boons, Marc M. S. M. Wösten, and Tom Wennekes**

anie_202107181_sm_miscellaneous_information.pdf
anie_202107181_sm_SI.xlsx

Supporting Information

Table of Contents

1. Chemical Synthesis **2**

1.1 General Methods 2

1.2 Synthesis of Leg precursor 1 and its azido-analogues (2-7). 2

2. Biology **9**

2.1 Bacterial strains, plasmids and growth conditions 9

2.2 Complementation of the Δ*maf4* mutant 9

2.3 Metabolic labeling of bacterial strains 9

2.4 Visualization of live bacteria 10

2.5 MS analysis of CMP-LegdiNAz from *C. jejuni* 10

2.6 Sequence alignment and *in silico* analysis of Maf4 11

3. Supplemental Figures **12**

4. References for Supporting Information **18**

5. NMR spectrum **19**

1. Chemical Synthesis

1.1 General Methods

All reagents were purchased from commercial sources unless otherwise stated. All reactions were carried out in oven-dried glassware with anhydrous organic solvents under argon atmosphere at room temperature unless otherwise specified. Thin layer chromatography (TLC) on silica gel-coated aluminum or glass plates was used to monitor the reaction progress. TLC plates were visualized by UV light (254 nm), and/or by charring with sulfuric acid (10% in ethanol) or a solution of $(NH_4)_6Mo_7O_{24}\cdot 4H_2O$ (24.0 g, 19.4 mmol), $Ce(NH_4)_2(NO_3)_6$ (0.50 g, 0.9 mmol) and sulfuric acid (28 mL) in water (500 mL). Organic solutions were concentrated under reduced pressure with a water bath at 40 °C. Flash column chromatography was performed on silica gel G60 (Silicycle, 60-200 μm, 60 Å). NMR spectra were recorded on a Bruker 600 MHz instrument or a Varian Mercury 400 MHz instrument at room temperature. Chemical shifts are reported in parts per million (ppm). Tetramethylsilane (TMS) and the residual solvents (D_2O or CD_3OD or $CDCl_3$) were used in spectra as internal standards when applicable. NMR data are presented as follows: chemical shift, multiplicity (s = singlet, bs = broad singlet, d = doublet, t = triplet, dd = doublet of doublets, td = triplet of doublets, q = quartet, dq = doublet of quartets and m = multiplet and/or multiple resonances), integration and coupling constants reported as Hertz (Hz). NMR signals were assigned based on ^1H NMR, ^{13}C NMR, COSY, HSQC and HMBC experiments. Mass spectra were recorded on a Kratos Analytical Maxima-CFR MALDI-TOF system (2,5-dihydroxybenzoic acid as matrix) or a Bruker micrOTOF-QII (ESI LC-MS). High resolution masses were recorded on an Agilent 6560 Ion Mobility Q-TOF LC-MS system.

1.2 Synthesis of Leg precursor 1 and its azido-analogues (2-7).

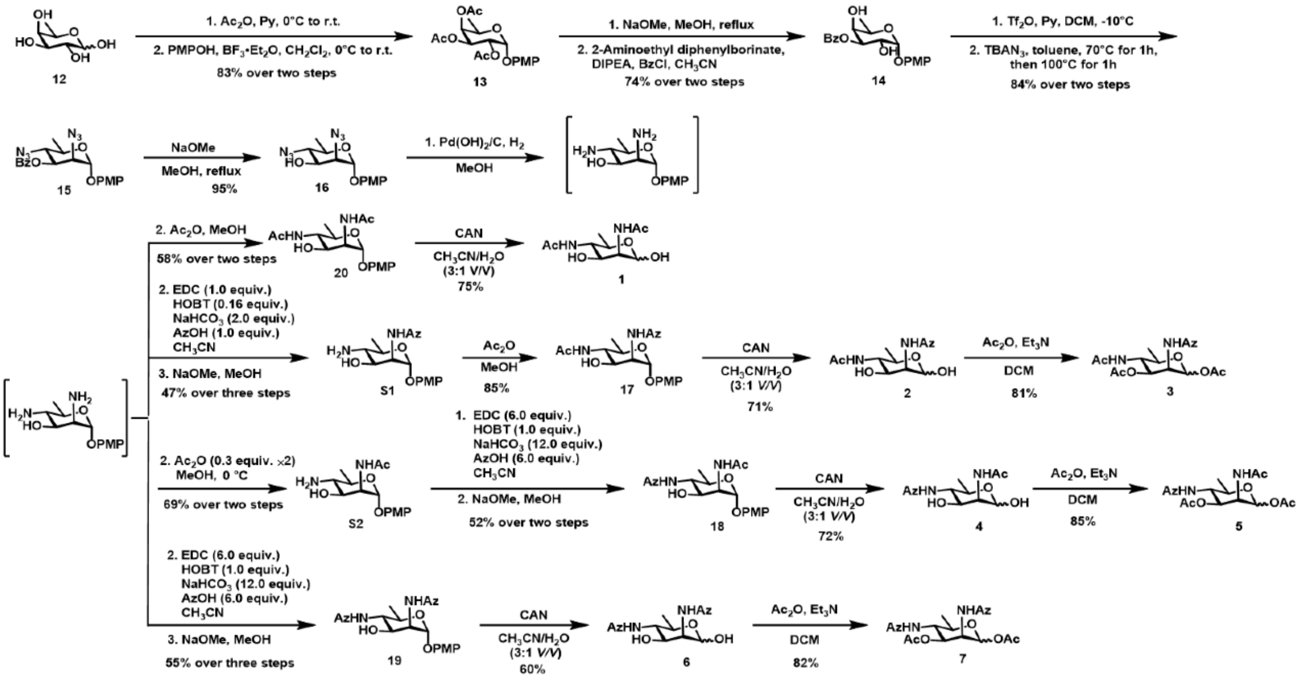

Scheme S1. Synthesis of Leg precursor **1** and its azido-analogues (**2-7**).

p-Methoxyphenyl-2,3,4-tri-*O*-acetyl-α-D-fucopyranoside (13)

To a solution of D-fucose **12** (9.00 g, 54.82 mmol) in pyridine (54 mL) was added Ac_2O (45 mL) dropwise at 0 °C. After stirring at 0 °C for 1 h, the mixture was then warmed to room temperature and stirred for 21 h. The solvent was removed *in vacuo* and the residue was co-evaporated with toluene (60 mL, 4 times). The acetylated D-fucose was dried *in vacuo* for ~6 h and directly used in the next step without further purification. To a mixture of the above peracetate (16.00 g, 48.13 mmol) and *p*-methoxyphenol (8.96 g, 72.20 mmol) in anhydrous CH_2Cl_2

(125 mL) under argon was added $BF_3 \cdot OEt_2$ (11.89 mL, 96.28 mmol) dropwise at 0°C. After stirring at this temperature for 2 h, the mixture was then warmed to room temperature, stirred for 18 h and diluted with CH_2Cl_2 (80 mL). The organic layer was washed with water, saturated $NaHCO_3$ and brine respectively, then dried over Na_2SO_4. After filtration, the solvent was removed under reduced pressure and the residue was purified by silica gel chromatography using petroleum ether : EtOAc (8:1 to 2:1 *v/v*) as an eluent to give compound **13** as a white solid (15.8 g, 83% over two steps). R_f = 0.25 (petroleum ether/ EtOAc, 8:1 *v/v*). ^{1}H NMR (600 MHz, $CDCl_3$) δ 6.98 (d, *J* = 9.0 Hz, 2H, Ar-*H*), 6.83 (d, *J* = 9.0 Hz, 2H, Ar-*H*), 5.62 (d, *J* = 3.6 Hz, 1H, H-1), 5.57 (dd, *J* = 10.9, 3.4 Hz, 1H, H-3), 5.36 (dd, *J* = 3.5, 1.3 Hz, 1H, H-4), 5.25 (dd, *J* = 10.9, 3.6 Hz, 1H, H-2), 4.37-4.26 (m, 1H, H-5), 3.77 (s, 3H, C*H*$_3$O), 2.19 (s, 3H, C*H*$_3$CO), 2.07 (s, 3H, C*H*$_3$CO), 2.02 (s, 3H, C*H*$_3$CO), 1.14 (d, *J* = 6.6 Hz, 3H, H-6). ^{13}C NMR (151 MHz, $CDCl_3$) δ 170.75, 170.64, 170.29, 155.35, 150.79, 117.91, 114.83, 95.81, 71.19, 68.12, 68.09, 65.36, 55.82, 20.95, 20.89, 20.82, 16.04. HRMS (ESI): m/z calcd. for $C_{19}H_{24}NaO_9$ $[M + Na]^+$ 419.1318, found 419.1337.

***p*-Methoxyphenyl-3-*O*-benzoyl-α-D-fucopyranoside (14)**

Sodium methoxide (196 μL, 30% in MeOH) was added to a solution of compound **13** (4.20 g, 10.59 mmol) in MeOH (50 mL). While being stirred vigorously, the reaction mixture was heated to 45°C for 2 h. After being cooled to room temperature, the reaction mixture was neutralized with Amberlite (H^+), filtered and concentrated under reduced pressure. This intermediate was dried *in vacuo* for 5-6 h before used in the next step without further purification. 2-Aminoethyl diphenylborinate (110 mg, 0.44 mmol) and the crude product (1.00 g, 4.44 mmol) were placed in a 50 mL round bottom flask, dried under vacuum for 30 min, then dissolved in dry acetonitrile (23 mL). *N*,*N*-Diisopropylethylamine (1.03 mL, 8.88 mmol) and benzoyl chloride (1.55 mL, 8.88 mmol) were added and the resulting mixture was stirred at room temperature for 5h. After the completion of the reaction as indicated by TLC, the reaction mixture was then diluted with EtOAc (150 mL), washed with water (30 mL), and extracted with EtOAc (30 mL, three times). The combined organic layers were dried over anhydrous Na_2SO_4, filtered, and concentrated under reduced pressure. The resulting crude material was purified by silica gel chromatography using CH_2Cl_2 : EtOAc (10:1 *v/v*) as an eluent to afford compound **14** as a white solid (1.23 g, 74% over two steps). R_f = 0.52 (CH_2Cl_2 / EtOAc, 9:1 *v/v*). ^{1}H NMR (600 MHz, $CDCl_3$) δ 8.11 (d, *J* = 7.9 Hz, 2H, Ar*H*), 7.58 (t, *J* = 7.4 Hz, 1H, Ar*H*), 7.45 (t, *J* = 7.5 Hz, 2H, Ar*H*), 7.05 (d, *J* = 8.4Hz, 2H, Ar*H*), 6.85 (d, *J* = 8.5 Hz, 2H, Ar*H*), 5.52-5.50 (m, 2H, H-1, H-3), 4.31-4.23 (m, 2H, H-5, H-2), 4.04 (s, 1H, H-4), 3.78 (s, 3H, C*H*$_3$O), 2.27 (d, *J* = 11.3 Hz, 1H, O*H*), 2.24 (d, *J* = 4.3 Hz, 1H, O*H*), 1.28 (d, *J* = 6.6 Hz, 3H, H-6). ^{13}C NMR (151 MHz, $CDCl_3$) δ 165.63, 155.46, 149.89, 133.95, 130.17, 128.80, 117.69, 114.85, 97.10, 72.59, 67.92, 63.13, 61.39, 55.77, 18.52. HRMS (ESI): m/z calcd. for $C_{20}H_{22}NaO_7$ $[M + Na]^+$ 397.1263, found 397.1292.

***p*-Methoxyphenyl-2,4-diazido-3-*O*-benzoyl-6-deoxy-α-D-mannopyranoside (15)**

Trifluoromethanesulfonic anhydride (7.48 mL, 44.45 mmol) was added dropwise at -10 °C to a solution of compound **14** (2.08 g, 5.56 mmol) and pyridine (4.49 mL, 55.60 mmol) in dry CH_2Cl_2 (33 mL). The temperature was slowly increased to 0 °C over a period of 1 h and the mixture was stirred at 0 °C for another 1 h. The reaction mixture was diluted with CH_2Cl_2 (50 mL) and washed successively with a solution of 1N HCl and saturated brine solution. The organic phase was dried over anhydrous Na_2SO_4. After filtration, the solvent was removed under reduced pressure at room temperature and the resulting residue was dried *in vacuo* for ~2h and directly used for next step without further purification. The crude 2,4-bis-triflate obtained was dissolved in toluene (35 mL) and tetra-*n*-butylammonium azide (4.74 g, 16.67 mmol) was added. While being stirred vigorously, the reaction mixture was heated to 70°C for 1 h, then the temperature was increased to 110 °C and

the reaction mixture was stirred for 1h. After being cooled to room temperature, the solvent was carefully removed under reduced pressure at room temperature and the condensed residue was diluted with CH_2Cl_2 (50 mL). The organic layer was washed with saturated brine solution and dried over anhydrous Na_2SO_4. After filtration, the solvent was removed under reduced pressure at room temperature and the resulting crude material was purified by flash column chromatography on silica gel using petroleum ether : EtOAc (10:1 to 7:1 *v/v*) as an eluent to give compound **15** as a white solid (1.97 g, 84 % over two steps). R_f = 0.66 (petroleum ether/ EtOAc, 7:1 *v/v*). ^{1}H NMR (600 MHz, $CDCl_3$) δ 8.15 (dd, *J* = 8.4, 1.4 Hz, 2H, Ar*H*), 7.66-7.61 (m, 1H, Ar*H*), 7.51 (dd, *J* = 8.2, 7.4 Hz, 2H, Ar*H*), 7.02 (d, *J* = 9.1 Hz, 1H, Ar*H*), 6.85 (d, *J* = 9.1 Hz, 1H, Ar*H*), 5.72 (dd, *J* = 10.1, 3.8 Hz, 1H, H-3), 5.40 (d, *J* = 1.8 Hz, 1H, H-1), 4.38 (dd, *J* = 3.8, 1.8 Hz, 1H, H-2), 3.89-3.82 (m, 1H, H-5), 3.78 (s, 3H, OC*H*$_3$), 3.76 (t, *J* = 10.1 Hz, 1H, H-4), 1.38 (d, *J* = 6.2 Hz, 3H, H-6). ^{13}C NMR (151 MHz, $CDCl_3$) δ 165.66, 155.48, 149.93, 133.99, 130.20, 128.83, 117.71, 114.88, 97.13, 72.61, 67.95, 63.16, 61.42, 55.81, 18.55.

***p*-Methoxyphenyl-2,4-diazido-6-deoxy-α-D-mannopyranoside (16)**

Sodium methoxide (377 μL, 2M in MeOH) was added to a solution of compound **15** (1.60 g, 3.77 mmol) in MeOH (25 mL). While being stirred vigorously, the reaction mixture was heated to 45°C for 1.2 h. TLC was used to monitor the completion of the reaction. After being cooled to room temperature, the reaction mixture was neutralized with Amberlite (H^+), filtered and concentrated under reduced pressure. The residue was purified by flash column chromatography on silica gel using petroleum ether : EtOAc (7:1 *v/v*) as an eluent to afford **16** as a white solid (1.15 g, 95 %). R_f = 0.42 (petroleum ether/ EtOAc, 7:1 *v/v*). ^{1}H NMR (600 MHz, $CDCl_3$) δ 6.96 (d, *J* = 9.1 Hz, 2H, Ar*H*), 6.83 (d, *J* = 9.1 Hz, 2H, Ar*H*), 5.39 (d, *J* = 1.8 Hz, 1H, H-1), 4.22 (ddd, *J* = 10.1, 6.7, 3.8 Hz, 1H, H-3), 4.09 (dd, *J* = 3.9, 1.7 Hz, 1H, H-2), 3.78 (s, 3H, C*H*$_3$O), 3.71 (dq, J = 9.9, 6.2 Hz, 1H, H-5), 3.38 (t, *J* = 9.9 Hz, 1H, H-4), 2.44 (d, *J* = 6.6 Hz, 1H, O*H*), 1.32 (d, *J* = 6.2 Hz, 3H, H-6). ^{13}C NMR (151 MHz, $CDCl_3$) δ 155.42, 149.98, 117.64, 114.86, 97.09, 70.28, 67.94, 66.26, 63.33, 55.80, 18.44.

***p*-Methoxyphenyl-2,4-diacetamido-6-deoxy-α-D-mannopyranoside (20)**

A solution of compound **16** (65 mg, 0.203 mmol) in MeOH (2mL) was hydrogenated with 20% $Pd(OH)_2$/C (18 mg) under the atmosphere of hydrogen at 1 atm for 12 h at room temperature. TLC was used to monitor the completion of the reaction. The catalyst was filtered off through a Celite pad and the filtrate was concentrated to dryness under reduced pressure. The crude residue was dissolved in MeOH (1 mL) and Ac_2O (47 μL, 0.492 mmol) was added, after which the mixture was stirred for 1 h at room temperature. The residue obtained after evaporation of the solvent was purified by flash column chromatography on silica gel using CH_2Cl_2 : MeOH (9:1 *v/v*) as an eluent to afford product **20** as a white solid (41 mg, 58 % over two steps). R_f = 0.24 (CH_2Cl_2 / MeOH, 9:1 *v/v*). ^{1}H NMR (600 MHz, CD_3OD) δ 6.99 (d, *J* = 9.1 Hz, 2H, Ar*H*), 6.84 (d, *J* = 9.1 Hz, 2H, Ar*H*), 5.34 (d, *J* = 1.6 Hz, 1H, H-1), 4.44 (dd, *J* = 4.7, 1.7 Hz, 1H, H-2), 4.15 (dd, *J* = 10.6, 4.8 Hz, 1H, H-3), 3.90 (t, *J* = 10.4 Hz, 1H, H-4), 3.80 (dq, J = 10.1, 6.2 Hz, 1H, H-5), 3.74 (s, 3H, C*H*$_3$O), 2.07 (s, 3H, C*H*$_3$CO), 1.99 (s, 3H, C*H*$_3$CO), 1.16 (d, *J* = 6.2 Hz, 3H, H-6). ^{13}C NMR (151 MHz, CD_3OD) δ 174.43, 174.06, 156.58, 151.65, 118.81, 115.62, 99.24, 69.11, 68.15, 56.03, 54.87, 54.17, 22.86, 22.58, 18.15. HRMS (ESI): m/z calcd. for $C_{17}H_{24}N_2NaO_6$ $[M + Na]^+$ 375.1532, found 375.1566.

2,4-Diacetamido-2,4,6-trideoxy-D-mannose (1)

To a solution of compound 20 (22 mg, 62.4 μmol) in CH_3CN/H_2O (1.5 mL, 3:1) was added cerium ammonium nitrate (103 mg, 0.188 mmol) at 0°C. The resulting clear orange solution was stirred for 1 h and then was warmed to room temperature and stirred for another 1 h. TLC was used to monitor the completion of the reaction. The reaction mixture was concentrated under reduced pressure, and the residue was purified by silica gel

chromatography using EtOAc : MeOH (10:1 *v/v*) as an eluent to give compound 1 as a white solid (11 mg, yield 75 %). R_f = 0.1 (EtOAc / MeOH, 10:1 *v/v*). α/β=1/1. ^{1}H NMR (600 MHz, D_2O) δ 5.21 (d, *J* = 1.6 Hz, 1H, H-1α), 5.06 (d, *J* = 1.6 Hz, 1H, H-1β), 4.57 (dd, *J* = 4.4, 1.6 Hz, 1H, H-2β), 4.40 (dd, *J* = 4.5, 1.6 Hz, 1H, H-2α), 4.16 (dd, *J* = 10.8, 4.6 Hz, 1H, H-3α), 4.11-4.04 (m, 1H, H-5α), 3.94 (dd, *J* = 10.8, 4.4 Hz, 1H, H-3β), 3.88 (t, *J* = 10.5 Hz, 1H, H-4α), 3.77 (t, *J* = 10.4 Hz, 1H, H-4β), 3.65-3.57 (m, 1H, H-5β), 2.22 (s, 3H, CH_3COβ), 2.17 (s, 3H, CH_3COα), 2.14 (s, 3H, CH_3COα), 2.13 (s, 3H, CH_3COβ), 1.32 (d, *J* = 6.2 Hz, 3H, H-6β), 1.29 (d, *J* = 6.3 Hz, 3H, H-6α). ^{13}C NMR (151 MHz, D_2O) δ 175.88, 174.94, 174.78, 174.71, 92.79, 92.76, 71.60, 69.99, 67.01, 66.56, 53.59, 53.56, 53.20, 52.72, 22.14, 22.12, 22.10, 21.94, 16.82, 16.78. HRMS (ESI): m/z calcd. for $C_{10}H_{18}N_2NaO_5$ $[M + Na]^+$ 269.1113, found 269.1144.

_p_-Methoxyphenyl-2,4-diazidoacetamido-2,4,6-trideoxy-α-D-mannopyranoside (19)

A solution of compound **16** (65 mg, 0.203 mmol) in MeOH (2mL) was hydrogenated with 20% $Pd(OH)_2$/C (18 mg) under the atmosphere of hydrogen at 1 atm for 12 h at room temperature. TLC was used to monitor the completion of the reaction. The catalyst was filtered off through a Celite pad and the filtrate was concentrated to dryness under reduced pressure. The residue obtained was dissolved in CH_3CN (2.5 mL), to this $NaHCO_3$ (202 mg, 2.4 mmol) and azido acetic acid (90μL, 1.2 mmol) were added. After 5 min, EDC•HCl (460 mg, 1.2 mmol) and HOBt (30 mg, 0.2 mmol) were added and the stirring was continued at room temperature for 5 h. After completion of the reaction indicated by TLC, solvent was removed under reduced pressure. The crude residue was dissolved in MeOH (1 mL) and to this mixture sodium methoxide (2M in MeOH) was added slowly to adjust the pH to 10 ~11, to remove any undesired esters formed in the first step. After 30 min, the reaction mixture was neutralized with Amberlite (H^+), filtered and the resin washed with MeOH (3×10 mL). The combined filtrate was concentrated and the crude product was purified by column chromatography on silica gel using CH_2Cl_2: MeOH (20:1 to 15:1 *v/v*) to afford the desired product **19** as a white solid (53 mg, 55% over three steps). R_f = 0.58 (CH_2Cl_2/MeOH, 10:1 *v/v*). ^{1}H NMR (600 MHz, CD_3OD) δ 7.01 (d, *J* = 9.1 Hz, 2H, Ar*H*), 6.85 (d, *J* = 9.1 Hz, 2H, Ar*H*), 5.36 (d, *J* = 1.6 Hz, 1H, H-1), 4.50 (dd, *J* = 4.7, 1.7 Hz, 1H, H-2), 4.23 (dd, *J* = 10.1, 4.8 Hz, 1H, H-3), 3.98 (dd, *J* = 15.8, 14.0 Hz, 2H, N_3CH_2CO), 3.93-3.84 (m, 4H, N_3CH_2CO, H-4, H-5), 3.75 (s, 3H, CH_3CO), 1.17 (d, *J* = 5.8 Hz, 3H, H-6). ^{13}C NMR (151 MHz, CD_3OD) δ 171.27, 170.94, 156.66, 151.55, 118.86, 115.65, 99.18, 68.92, 67.82, 56.04, 55.06, 54.12, 53.08, 52.50, 18.12. HRMS (ESI): m/z calcd. for $C_{17}H_{22}N_8NaO_6$ $[M + Na]^+$ 457.1560, found 457.1553.

2,4-Diazidoacetamido-2,4,6-trideoxy-D-mannose (6)

To a solution of compound **19** (138 mg, 0.317 mmol) in CH_3CN/H_2O (4 mL, 3:1) at 0 °C was added cerium ammonium nitrate (523 mg, 0.953 mmol). The resulting clear orange solution was stirred for 1 h and was next warmed to room temperature followed by stirring for another 2 h. TLC was used to monitor the completion of the reaction. The reaction mixture was concentrated under reduced pressure, and the residue was purified by silica gel chromatography using EtOAc : MeOH (20:1 *v/v*) as an eluent to give compound **6** as a white solid (61 mg, yield 60%). R_f = 0.21 (CH_2Cl_2/MeOH, 15:1 *v/v*). α/β=5/3. ^{1}H NMR (600 MHz, CD_3OD) δ 5.03 (d, *J* = 1.7 Hz, 1H, H-1α), 4.83 (d, *J* = 1.6 Hz, 0.6H, H-1β), 4.46 (dd, *J* = 4.2, 1.6 Hz, 0.6H, H-2β), 4.29 (dd, *J* = 4.6, 1.7 Hz, 1H, H-2α), 4.09 (dd, *J* = 10.7, 4.5 Hz, 1H, H-3α), 4.04-3.85 (m, 7H, H-5β, N_3CH_2COαβ), 3.81-3.75 (m, 1.6H, H-3β, H-4α), 3.72-3.63 (m, 0.6H, H-4β), 3.47-3.39 (m, 0.6H, *H*-5β), 1.23 (d, *J* = 6.2 Hz, 1.8H, H-6β), 1.18 (d, *J* = 6.3 Hz, 3H, H-6α). ^{13}C NMR (151 MHz, CD_3OD) δ 172.00, 171.07, 171.05, 170.90, 94.66, 94.42, 72.79, 71.66, 67.88, 67.68, 55.54, 55.40, 55.10, 54.87, 53.12, 53.05, 52.76, 52.59, 18.29, 18.23. HRMS (ESI): m/z calcd. for $C_{10}H_{16}N_8NaO_5$ $[M + Na]^+$ 351.1141, found 351.1137.

2,4-Diazidoacetamido-1,3-di-_O_-acetyl-2,4,6-trideoxy-D-mannopyranoside (7)

Compound **6** (18 mg, 0.054 mmol) was dissolved in CH_2Cl_2 (2 mL) and to this mixture Et_3N (152μL, 1.08 mmol)

and Ac_2O (32µL, 0.32 mmol) were added. After stirring at room temperature for 12 h, solvent was removed *in vacuo* and the crude product was purified by silica gel chromatography using CH_2Cl_2 : MeOH (20:1 *v/v*) as an eluent to afford the desired product **7** as a white solid (22 mg, yield 82 %). R_f = 0.48 (CH_2Cl_2/MeOH, 15:1 *v/v*). α/β=10/9 ^{1}H NMR (600 MHz, CD_3OD) δ 5.93 (d, *J* = 1.8 Hz, 1H, H-1α), 5.82 (d, *J* = 1.7 Hz, 0.9H, H-1β), 5.25 (dd, *J* = 11.1, 4.4 Hz, 1H, H-3α), 5.05 (dd, *J* = 11.1, 4.2 Hz, 0.9H, H-3β), 4.70 (dd, *J* = 4.2, 1.7 Hz, 0.9H, H-2β), 4.55 (dd, *J* = 4.5, 1.8 Hz, 1H, H-2α), 4.09 (t, *J* = 10.7 Hz, 1H, H-4α), 4.03-3.90 (m, 5.7H, H-5α, H-4β, N_3CH_2COαβ), 3.88 (d, *J* = 1.3 Hz, 1.8H, N_3CH_2COβ), 3.87 (d, *J* = 1.3 Hz, 2H, N_3CH_2COα), 3.68-3.62 (m, 0.9H, H-5β), 2.15, 1.97, 2.07, 1.97, 1.97 (s, 11.4H, 4 CH_3CO), 1.27 (d, *J* = 6.2 Hz, 2.7H, H-6β), 1.23 (d, *J* = 6.2 Hz, 3H, H-6α). ^{13}C NMR (151 MHz, CD_3OD) δ 172.00, 171.87, 171.28, 170.89, 170.71, 170.64, 170.24, 170.16, 93.47, 92.10, 73.67, 72.47, 70.62, 70.22, 53.08, 53.07, 52.45, 52.33, 51.85, 51.82, 50.88, 50.23, 20.79, 20.74, 20.70, 20.63, 18.02, 17.90. HRMS (ESI): m/z calcd. for $C_{14}H_{20}N_8NaO_7$ $[M + Na]^+$ 351.1141, found 351.1137.

***p*-Methoxyphenyl-4-acetamido-2-azidoacetamido-2,4,6-trideoxy-α-D-mannopyranoside (S1)**

A solution of compound **16** (96 mg, 0.3 mmol) in MeOH (5 mL) was hydrogenated with 20% $Pd(OH)_2/C$ (30mg) under the atmosphere of hydrogen at 1 atm for 12 h at room temperature. TLC was used to monitor the completion of the reaction. The catalyst was filtered off through a Celite pad and the filtrate was concentrated to dryness under reduced pressure. The crude residue (79 mg, 0.29mmol) was dissolved in CH_3CN (3 mL), $NaHCO_3$ (49mg, 0.58 mmol) and azido acetic acid (22 µL, 0.29 mmol) were added. After 5 min, EDC•HCl (56 mg, 0.29mmol) and HOBT(6 mg, 46.4 µmol) were added at 0°C and the stirring was continued at room temperature for 1h. (*To avoid the byproduct **19**, TLC analysis is important to monitor the reaction progress). After completion of the reaction indicated by TLC, solvent was removed under reduced pressure. The crude residue was dissolved in MeOH (2 mL) and sodium methoxide (2M in MeOH) was added slowly to adjust the pH to 10 ~11. After 30 min, the reaction mixture was neutralized with Amberlite (H^+) and filtered. Filtrate was concentrated and the crude product was purified by silica gel chromatography using CH_2Cl_2 : MeOH (20:1 *v/v*) as an eluent to produce compound **S1** as a white foam (49 mg, yield 47%). R_f = 0.22 (CH_2Cl_2/MeOH, 11:1 *v/v*). ^{1}H NMR (600 MHz, CD_3OD) δ 7.00 (d, *J* = 9.1 Hz, 2H, Ar*H*), 6.85 (d, *J* = 9.1 Hz, 2H, Ar*H*), 5.34 (d, *J* = 1.5 Hz, 1H, H-1), 4.42 (dd, *J* = 4.7, 1.6 Hz, 1H, H-2), 4.02 (dd, *J* = 10.3, 4.7 Hz, 1H, H-3), 3.96 (d, *J* = 9.6 Hz, 2H, N_3CH_2CO), 3.77-3.73 (m, 4H, H-5, CH_3CO), 2.72 (t, *J* = 10.0 Hz, 1H, H-4), 1.24 (d, *J* = 6.3 Hz, 3H, H-6). ^{13}C NMR (151 MHz, CD_3OD) δ 171.07, 156.61, 151.63, 118.92, 115.61, 99.47, 70.41, 69.74, 56.03, 54.00, 52.55, 18.22. HRMS (ESI): m/z calcd. for $C_{15}H_{22}N_2NaO_5$ $[M + Na]^+$ 374.1440, found 374.1469.

***p*-Methoxyphenyl-4-acetamido-2-azidoacetamido-2,4,6-trideoxy-α-D-mannopyranoside (17)**

The compound **S1** (53 mg, 0.15 mmol) was dissolved in MeOH (2.5 mL), Ac_2O (29 µl, 0.30 mmol) was added and the mixture was stirred for 2 h at room temperature. TLC was used to monitor the completion of the reaction. The mixture was concentrated under reduced pressure, and the residue was purified by silica gel chromatography using CH_2Cl_2 : MeOH (20:1 *v/v*) as an eluent to give compound **17** as a white solid (50 mg, 85%). R_f = 0.38 (CH_2Cl_2/ MeOH, 11:1 *v/v*). ^{1}H NMR (600 MHz, CD_3OD) δ 7.00 (d, *J* = 9.1 Hz, 2H, Ar*H*), 6.85 (d, *J* = 9.1 Hz, 2H, Ar*H*), 5.36 (d, *J* = 1.6 Hz, 1H, H-1), 4.48 (dd, *J* = 4.8, 1.7 Hz, 1H, H-2), 4.16 (dd, *J* = 10.3, 4.7 Hz, 1H, H-3), 3.98 (dd, J = 15.8, 13.3 Hz, 2H, N_3CH_2CO), 3.88-3.78 (m, 2H, H-5, H-4), 3.75 (s, 3H, CH_3O), 1.99 (s, 3H, CH_3CO), 1.16 (d, *J* = 6.0 Hz, 3H, H-6). ^{13}C NMR (151 MHz, CD_3OD) δ 174.00, 171.24, 156.64, 151.56, 118.83, 115.64, 99.13, 69.15, 68.08, 56.03, 54.89, 54.15, 52.51, 22.85, 18.14. HRMS (ESI): m/z calcd. for $C_{17}H_{23}N_5NaO_6$ $[M + Na]^+$ 416.1546, found 416.1592.

4-Acetamido-2-azidoacetamido-2,4,6-trideoxy-D-mannose (2)

To a solution of compound **17** (26 mg, 0.066 mmol) in CH_3CN/H_2O (1.6 mL, 3:1) at 0 °C was added cerium ammonium nitrate (109 mg, 0.198 mmol). The resulting clear orange solution was stirred for 1 h and then was

warmed to room temperature and was stirred for 1 h. TLC was used to monitor the completion of the reaction. The reaction mixture was concentrated under reduced pressure, and the residue was purified by silica gel chromatography using CH_2Cl_2 : MeOH (20:1 *v/v*) as an eluent to give compound **2** as a white solid (13mg, yield 71 %). R_f = 0.25 (CH_2Cl_2 / MeOH, 7:1 *v/v*). α/β=3/1. ^{1}H NMR (600 MHz, CD_3OD) δ 5.02 (d, *J* = 1.6 Hz, 1H, H-1α), 4.81 (d, *J* = 1.6 Hz, 0.3H, H-1β), 4.45 (dd, *J* = 4.2, 1.6 Hz, 0.3H, H-2β), 4.27 (dd, *J* = 4.6, 1.7 Hz, 1H, H-2α), 4.04-3.90 (m, 4.7H, H-3α, $N_3C\mathit{H_2}CO$αβ, H-5α), 3.74 (t, *J* = 10.4 Hz, 1H, H-4α), 3.65-3.61 (m, 0.3H, H-4β), 3.38-3.34 (m, 0.3H, H-5β), 1.98 (s, 3H, $C\mathit{H_3}CO$α), 1.98 (s, 1H, $C\mathit{H_3}CO$β), 1.22 (d, *J* = 6.2 Hz, 1H, H-6β), 1.17 (d, *J* = 6.3 Hz, 3H, H-6α). ^{13}C NMR (151 MHz, CD_3OD) δ 174.00, 173.98, 171.97, 171.03, 94.65, 94.36, 73.11, 71.95, 68.11, 67.89, 55.52, 55.21, 54.89, 52.74, 52.57, 22.89, 22.83, 18.30, 18.25. HRMS (ESI): m/z calcd. for $C_{10}H_{17}N_5NaO_5$ $[M + Na]^+$ 310.1127, found 310.1193.

4-Acetamido-2-azidoacetamido-1,3-di-*O*-acetyl-2,4,6-trideoxy-D-mannopyranoside (3)

Compound **2** (8 mg, 0.028 mmol) was dissolved in CH_2Cl_2 (2 mL) and to this mixture Et_3N (39 µL, 0.28 mmol) and Ac_2O (14µL, 0.14 mmol) were added. TLC was used to monitor the completion of the reaction. After stirring at room temperature for 7 h, The reaction mixture was concentrated under reduced pressure, and the residue was purified by silica gel chromatography using CH_2Cl_2 : MeOH (20:1 *v/v*) as an eluent to give compound **3** as a white solid (7 mg, yield 81 %). R_f = 0.19 (CH_2Cl_2 / MeOH, 20:1 *v/v*). α/β=1/1. ^{1}H NMR (600 MHz, CD_3OD) δ 5.93 (d, *J* = 1.8 Hz, 1H, H-1α), 5.81 (d, *J* = 1.7 Hz, 1H, H-1β), 5.18 (dd, *J* = 11.1, 4.4 Hz, 1H, H-3α), 4.98 (dd, *J* = 11.0, 4.2 Hz, 1H, H-3β), 4.69 (dd, *J* = 4.2, 1.7 Hz, 1H, H-2β), 4.53 (dd, *J* = 4.5, 1.8 Hz, 1H, H-2α), 4.04 (t, *J* = 10.7 Hz, 1H, H-4α), 3.99-3.93 (m, 5H, H-4β, $N_3C\mathit{H_2}CO$αβ), 3.92-3.85 (m, 1H, H-5α), 3.58 (dq, *J* = 10.1, 6.2 Hz, 1H, H-5β), 2.15 (s, 3H, $C\mathit{H_3}CO$), 2.06 (s, 3H, $C\mathit{H_3}CO$), 1.97 (s, 3H, $C\mathit{H_3}CO$), 1.97 (s, 3H, $C\mathit{H_3}CO$), 1.94 (s, 6H, 2 $C\mathit{H_3}CO$), 1.26 (d, *J* = 6.1 Hz, 3H, H-6β), 1.22 (d, *J* = 6.2 Hz, 3H, H-6α). ^{13}C NMR (151 MHz, CD_3OD) δ 173.67, 173.59, 172.01, 171.88, 171.26, 170.89, 170.26, 170.16,93.46, 92.12,73.98, 72.67, 70.92, 70.40, 52.45, 52.33, 51.65, 51.62, 50.86, 50.22, 22.72, 22.68, 20.78, 20.74, 20.69, 20.64, 18.04, 17.91. HRMS (ESI): m/z calcd. for $C_{14}H_{21}N_5NaO_7$ $[M + Na]^+$ 394.1339, found 394.1359.

***p*-Methoxyphenyl-4-amino-2-azidoacetamido-2,4,6-trideoxy-α-D-mannopyranoside (S2)**

A solution of compound **16** (90 mg, 0.28 mmol) in MeOH (4 mL) was hydrogenated with 20% $Pd(OH)_2/C$ (25 mg) under the atmosphere of hydrogen at 1 atm for 8 h at room temperature. TLC was used to monitor the completion of the reaction. The catalyst was filtered off through Celite and the filtrate was concentrated to dryness. The crude residue (73mg, 0.27 mmol) was dissolved in MeOH (2 mL) and cooled to 0°C, Ac_2O (7.6 µL, 81.7 µmol) was added dropwise and the mixture was stirred for 1h at 0°C. Then Ac_2O (7.6 µL, 81.7 µmol) was added dropwise in addition at 0°C and the mixture was stirred for another 1h at 0°C. The residue obtained after evaporation of the solvent, was purified by silica gel chromatography using CH_2Cl_2 : MeOH (10:1 to 7:1 *v/v*) as an eluent to afford **S2** as a light yellow solid (58 mg, 69%). R_f = 0.18 (CH_2Cl_2 /MeOH, 10:1 *v/v*). ^{1}H NMR (600 MHz, CD_3OD) δ 6.99 (d, *J* = 9.1 Hz, 2H, Ar*H*), 6.84 (d, *J* = 9.1 Hz, 2H, Ar*H*), 5.30 (d, *J* = 1.6 Hz, 1H, H-1), 4.41 (dd, *J* = 4.8, 1.6 Hz, 1H, H-2), 4.02 (dd, *J* = 10.4, 4.8 Hz, 1H, H-3), 3.79-3.73 (m, 4H, $C\mathit{H_3}O$, H-5), 2.82 (t, *J* = 10.1 Hz, 1H, H-4), 2.05 (s, 3H, $C\mathit{H_3}CO$), 1.25 (d, *J* = 6.3 Hz, 3H, H-6). ^{13}C NMR (151 MHz, CD_3OD) δ 174.30, 156.58, 151.67, 118.89, 115.60, 99.64, 70.07, 69.49, 56.03, 55.89, 53.95, 22.54, 18.19. HRMS (ESI): m/z calcd for $C_{15}H_{22}N_2NaO_5$ $[M + Na]^+$ 333.1426, found 333.1471.

***p*-Methoxyphenyl-4-acetamido-2-azidoacetamido-2,4,6-trideoxy-α-D-mannopyranoside (18)**

The compound **S2** (58mg, 0.187mmol, 6.0 eq) was dissolved in CH_3CN (2 mL), $NaHCO_3$ (188 mg, 2.244 mmol) and azido acetic acid (133 mg, 83µL, 1.122 mmol) were added to the reaction mixture. After 10 min, EDC•HCl (215mg, 1.122 mmol) and HOBT (25 mg, 0.187 mmol) were added at 0°C and the stirring was continued at room temperature for 3 h. After completion of the reaction indicated by TLC, solvent was removed under

reduced pressure. The crude residue was then dissolved in MeOH (1 mL) and to this mixture sodium methoxide (2M in MeOH) was added slowly to adjust the pH to 10 ~11. After 30 min, the reaction mixture was neutralized with Amberlite (H^+) and filtered. Filtrate was concentrated and the crude product was purified by column chromatography on silica gel using CH_2Cl_2 : MeOH (20:1 to 15:1 *v/v*) as an eluent to afford the desired product **18** as a white solid (38 mg, 52 % over two steps). R_f = 0.31 (CH_2Cl_2 / MeOH, 10:1 *v/v*). ^{1}H NMR (600 MHz, CD_3OD) δ 7.00 (d, *J* = 9.1 Hz, 2H, Ar*H*), 6.85 (d, *J* = 9.1 Hz, 2H, Ar*H*), 5.33 (d, *J* = 1.7 Hz, 1H, H-1), 4.46 (dd, *J* = 4.8, 1.7 Hz, 1H, H-2), 4.20 (dd, *J* = 10.6, 4.8 Hz, 1H, H-3), 3.96-3.83 (m, 4H, N_3CH_2CO, H-4, H-5), 3.75 (s, 3H, CH_3O), 2.07 (s, 3H, CH_3CO), 1.17 (d, *J* = 6.2 Hz, 3H, H-6). ^{13}C NMR (151 MHz, CD_3OD) δ 174.47, 170.99, 156.62, 151.65, 118.85, 115.63, 99.33, 68.91, 67.91, 56.04, 55.05, 54.15, 53.10, 22.57, 18.12. HRMS (ESI): m/z calcd. for $C_{17}H_{23}N_5NaO_6$ $[M + Na]^+$ 416.1546, found 416.1544.

4-Acetamido-2-azidoacetamido-2,4,6-trideoxy-mannose (4)

To a solution of compound **18** (28 mg, 0.071mmol) in CH_3CN/H_2O (1.2 mL, 3:1) at 0 °C was added cerium ammonium nitrate (117 mg, 0.213 mmol). The resulting clear orange solution was stirred for 1 h and then was warmed to room temperature and was stirred for another 4 h. TLC was used to monitor the completion of the reaction. The reaction mixture was concentrated under reduced pressure, and the residue was purified by silica gel chromatography using CH_2Cl_2 : MeOH (15:1 *v/v*) as an eluent to produce compound **4** as a white solid (14 mg, 72 %). R_f = 0.22 (CH_2Cl_2/MeOH, 9:1 *v/v*). α/β=20/1. (major peak selected for NMR) ^{1}H NMR (600 MHz, CD_3OD) δ 5.01 (d, *J* = 1.6 Hz, 1H, H-1), 4.26 (dd, *J* = 4.6, 1.6 Hz, 1H, H-2), 4.06 (dd, *J* = 10.7, 4.6 Hz, 1H, H-3), 3.98-3.85 (m, 3H, N_3CH_2COα, H-5), 3.82 (t, *J* = 10.4 Hz, 1H, H-4), 2.04 (s, 3H, CH_3CO), 1.18 (d, *J* = 6.3 Hz, 3H, H-6). ^{13}C NMR (151 MHz, CD_3OD) δ 174.35, 170.94, 94.53, 67.99, 67.71, 55.37, 54.90, 53.06, 22.59, 18.28. HRMS (ESI): m/z calcd. for $C_{10}H_{17}N_5NaO_5$ $[M + Na]^+$ 310.1127, found 310.1127.

4-Acetamido-2-azidoacetamido-1,3-di-*O*-acetyl-2,4,6-trideoxy-mannopyranoside (5)

Compound **4** (7 mg, 0.024 mmol) was dissolved in CH_2Cl_2 (1 mL) and to this mixture Et_3N (66 µL, 0.48 mmol) and Ac_2O (14 µL, 0.144 mmol) were added. After stirring at room temperature for 12 h, the solvent was removed *in vacuo* and the crude product was purified by silica gel chromatography using CH_2Cl_2 : MeOH (25:1 *v/v*) as an eluent to afford the desired product **5** as a white solid (7.5 mg, 85%). R_f = 0.37 (CH_2Cl_2 / MeOH, 12:1 *v/v*). α/β=2/1. ^{1}H NMR (600 MHz, CD_3OD) δ 5.90 (d, *J* = 1.8 Hz, 1H, H-1α), 5.79 (d, *J* = 1.7 Hz, 0.5H, H-1β), 5.23 (dd, *J* = 11.2, 4.5 Hz, 1H, H-3α), 5.02 (dd, *J* = 11.1, 4.2 Hz, 0.5H, H-3β), 4.69 (dd, *J* = 4.3, 1.7 Hz, 0.5H, H-2β), 4.52 (dd, *J* = 4.6, 1.8 Hz, 1H, H-2α), 4.13 (t, *J* = 10.7 Hz, 1H, H-4α), 4.04 (t, *J* = 10.5 Hz, 0.5H, H-4β), 3.96-3.93 (m, 1H, H-5α), 3.88 (d, *J* = 1.3 Hz, 1H, N_3CH_2COβ), 3.87 (d, *J* = 1.5 Hz, 2H, N_3CH_2COα), 3.63-3.61 (m, 0.5H, H-5β), 2.14 (s, 3H, CH_3COα), 2.06 (s, 1.5H, CH_3COβ), 2.06 (s, 1.5H, CH_3COβ), 2.04 (s, 3H, CH_3COα), 1.96 (s, 3H, CH_3COα), 1.96 (s, 1.5H, CH_3COβ), 1.27 (d, *J* = 6.2 Hz, 1.5H, H-6β), 1.23 (d, *J* = 6.2 Hz, 3H, H-6α). ^{13}C NMR (151 MHz, CD_3OD) δ 174.44, 173.92, 171.97, 171.87, 170.66, 170.61, 170.32, 170.22, 93.65, 92.29, 73.68, 72.59, 70.61, 70.27, 53.10, 53.07, 51.85, 51.82, 50.65, 50.16, 22.37, 22.34, 20.75, 20.71, 20.65, 18.01, 17.87. HRMS (ESI): m/z calcd. for $C_{14}H_{21}N_5NaO_7$ $[M + Na]^+$ 394.1339, found 394.1380.

2. Biology

2.1 Bacterial strains, plasmids and growth conditions

The bacterial strains and plasmids used in this study are listed in **Table S1**.

C. jejuni strains were routinely cultured on 5% saponin-lysed horse blood agar plates (Biotrading, the Netherlands) at 42°C or in Heart Infusion (HI) medium (Biotrading, the Netherlands) at 42°C shaking at 160 rpm, under micro-aerophilic conditions (5% O_2, 10% CO_2, 5% H_2, 80% N_2). When appropriate, growth media were supplemented with ampicillin (100 μg ml^{-1}), kanamycin (50 μg ml^{-1}) or chloramphenicol (20 μg ml^{-1}).

Table S1. Bacterial strains and plasmid used in this study

Strain	Genotype or relevant characteristics	Reference/Source
C.jejuni 11168	Wild type	[1]
C.jejuni 108	Wild type	[2]
Δ*maf4*	108 derivate *maf4*:: *cat*	[3]
C.jejuni 81116	Wild type	[4]
Δ*flaAB*	81116 derivate *flaAB*:: *cat*	[5]
C.jejuni RM1221	Wild type	[8]
C.jejuni 81-176	Wild type	[9]
**Plasmid**		
PMA1maf4	Shuttle vector containing *maf4* gene	This study

2.2 Complementation of the Δ*maf4* mutant

The *maf4* gene including its ribosomal binding site was PCR amplified from strain *C. jejuni* 108 using primers maf4F 5-CTCGAGAAATAATTAGGATTTCCAATGACCT-3 and maf4R 5-GGTACCTTCTCTCACTTTCTCACCCTTTCC-3. After digestion with XhoI and KpnI, the resulting 1874 bp fragment was cloned into the pMA1 vector behind the *C. jejuni metK* promoter[6], yielding pMA1maf4. The plasmid was introduced by electroporation into *C. jejuni* 108 *maf4* mutant, and kanamycin resistant transformants were selected.

2.3 Metabolic labeling of bacterial strains

C. jejuni strains were grown in HI at 42°C at 160 rpm under micro-aerobic conditions. Overnight cultured bacteria were diluted in 1 mL fresh HI medium (OD_{550} ~0.1) containing Az-probe (1 mM final concentration) and incubated at 42 °C. After growth for 4h (*C. jejuni* 11168) or 7h (*C. jejuni* 108, 81116), mid-exponentially grown *C. jejuni* cultures were adjusted to OD_{550} of 0.1. 100 μL of each culture was centrifuged (14000 rpm, 2 min), bacterial pellets were washed once with 100 μL phosphate buffer saline (PBS) and resuspended in 100 μL PBS.

<u>Probing for azide on cells via Cu(I)-free strain-promoted Alkyne-Azide Click Chemistry (SPAAC) reaction</u>

To probe for azido-labeled glycoconjugates on the cell surface, the cells were treated with 10 μM DBCO-PEG_4-biotin (Jena Bioscience) for 1 h at room temperature. After the reaction, the cells were pelleted at 14000 rpm for 2 min, washed once with 100 μL PBS and resuspended in 100 μL PBS, then 50 μL 3×Laemmli buffer was added to lyse the cells upon boiling at 95 °C for 3 min. The samples were stored at -20°C before use.

<u>Western blot analysis</u>

Samples (15 μL) were loaded onto a 12% SDS-PAGE gel. Proteins were transferred to polyvinylidene difluoride

(PVDF) membrane (Bio-Rad) using the Trans-Blot Turbo Transfer System (Bio-Rad). Non-specific binding to the membrane was blocked by incubation (1 h, RT) of the blot with blocking buffer (5% skim milk powder (Elk, Campina) in PBS with 0.5% Tween-20). Biotin was detected with Streptavidin-HRP probe (1:10000) diluted in washing buffer (1% skim milk in PBS with 0.1% Tween-20). After washes with washing buffer and PBS, reactive bands were visualized using Chemiluminescent Substrate (Bio Rad). Images were taken with ChemiDoc MP system (Bio-Rad).
For flagellin detection the PVDF membrane containing equal amount of samples was probed (1 h) with monoclonal antibody anti-FlaA/B CF17(1:1000)[7] followed by incubating for (1 h) with HRP-conjugated goat anti-rabit IgG antibody (Santa Cruz Biotechnology) (1:3000). After washes with washing buffer and PBS, reactive bands were visualized using Chemiluminescent Substrate (Bio Rad). Images were taken with ChemiDoc MP system (Bio-Rad).

PEG Mass shift of flagellin

The bacterial cells, treated and untreated with probe 6 (as described above), were treated with 1 mM DBCO-PEG-5kDa (Click Chemistry Tools) for 4 h at room temperature. After the reaction, the cells were pelleted at 14000 rpm for 2 min, washed once with 100 µL PBS and resuspended in 100 µL PBS, then 50 µL 3×Laemmli buffer was added to lyse the cells upon boiling at 95 °C for 3 min. The samples were stored at -20°C before Western blot analysis. (For the control samples, either the probe 6 or DBCO-PEG5K reagent was omitted during the labeling procedure.) Western blotting was then performed according to the western blot procedure described above to detect the flagellin. The stoichiometry of probe incorporation was quantified by calculating the relative band intensities of modified and unmodified protein.

2.4 Visualization of live bacteria

After growth for 4h (*C. jejuni* 11168), mid-exponentially grown *C. jejuni* cultures were adjusted to OD_{550} of 0.2. Next, 200 µL of culture was centrifuged (14000 rpm, 2 min), bacterial pellets were washed and resuspended with 200 µL PBS. Cells were incubated with 10 µM DBCO-PEG_4-Biotin for 1 h at room temperature. After incubation, cells were washed three times with 200 µL PBS, to remove any unbound DBCO-PEG_4-Biotin. Next Streptavidin-Alexa Fluor 488 (Thermo Fisher S11223) (1:1000) was added and reacted at room temperature. After 1h, the cells were washed again three times with 200 µL PBS to remove any remaining unbound streptavadin-dye conjugate. To stain the body of the bacteria, CellTrace Yellow (CTY) (1:1000) dye (Thermo Fisher Scientific) was added and incubated for 20 min at room temperature. Cells were spun at 14,000 rpm for 2 min and washed three times with 200 µL PBS. Finally the pelleted cells were resuspended in Prolong diamond mounting solution (Thermo Fisher Scientific). The resulting mixture (10µL) was dropped onto a glass slide and covered with a thin glass coverslip. Coverslips were cured for 24 h in a dark and dry place before imaging. Images were recorded with DeltaVision OMX V4 Blaze (GE) microscope coupled to a workstation running SoftWoRx vs 3.7 software (Cytiva, Seatle, USA). Default filter sets were used to detect Alexa 488 fluorescence and CellTrace Yellow fluorescence. Digital analysis and image processing were conducted using FIJI.

2.5 MS analysis of CMP-LegdiNAz from *C. jejuni*

Campylobacter jejuni strains were grown (with/without probe) to OD_{550} ~0.4. Then samples were immediately placed onto ice with NaCl. Following this, cells were transferred to Eppendorf vial and spun (pre-cooled centrifuge at 4 °C, 14000 rpm) for 1 min. Supernatant was discarded and the pellet was washed with 1 mL wash buffer (75 mM $(NH_4)_2CO_3$, pH 7.4). The pellet was stored at -80 °C until metabolite extraction. Metabolites were extracted at -20˚C with 1 mL cold extraction buffer (40:40:20, *v/v/v* acetonitrile : methanol : water) for 5 min and pelleted. After centrifugation (4 °C, 14000 rpm) for 3 minutes the resulting supernatants were dried

using a vacuum centrifuge and the tubes were stored at -80 °C until analysis.

UPLC-MS experiments were performed on an Agilent 6560 Ion Mobility iFunnel quadrupole time-off-flight (Q-TOF) with an Agilent 1290 Infinity LC system (Agilent Technologies, Santa Clara, CA, US). Experiments were performed using a SeQuant ZIC-HILIC 3µm 100Å (150 X 2.1mm) column. Solvent A was 50 mM NH_4HCO_2H pH 4 in MilliQ, solvent B was 100% acetonitrile. The column was run at 40°C at a flow of 0.1 ml/min starting at 75% B over a course of 30 min to 25% B, followed by a 10 minute equilibration at 75% B before starting the next run.

Extracted ion chromatograms were generated by adding all product mass peaks identified for a given calculated mass $[M-H]^-$ ionization state with a 20 ppm error range around the observed mass, summed across all isotopes with significant signal above baseline noise. Calculated and observed masses are for most abundant isotope peaks in all cases to avoid issues with low signal to noise for minor side product monoisotopic peaks. Peak areas were calculated using Agilent Masshunter.

2.6 Sequence alignment and *in silico* analysis of Maf4

Homologous protein modeling of Maf4 was generated on the SWISS-MODEL server (https://swissmodel.expasy.org/). Three- dimensional structural analysis and superimposition were performed using the program PyMOL Molecular Graphics System (http://www. pymol.org). The coordinates and structure of the Maf protein of *M.magneticum* AMB-1 was retrieved from the Protein Data Bank with accession numbers 5MU5. Eight homologous protein sequences of Maf4 (protein ID: ACC7728.1; NCBI genome accession number: EU448320.1) were selected. Alignment of Maf4 and its homologous protein sequences was generated using the ESPript 3.0.

3. Supplemental Figures

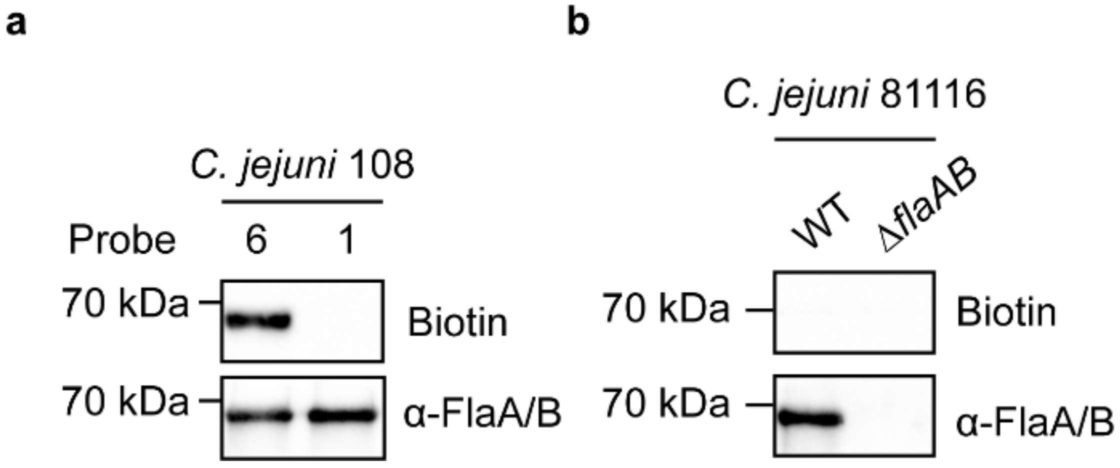

Figure S1. Western blot analysis of *C. jejuni* flagellins . a) *C. jejuni* 108 cells were grown in HI medium supplemented with 1 mM probe **6** or **1** for 7 h respectively, then incubated with 10 μM DBCO-PEG$_4$-Biotin for 1h in PBS and then lysed, and further analyzed by streptavidin blotting. b) *C. jejuni* 81116 and *C. jejuni* 81116 Δ*flaAB.* were grown in HI medium supplemented with 1 mM probe **6** for 7 h respectively, then incubated with 10 μM DBCO-PEG$_4$-Biotin for 1h in PBS and then lysed, and further analyzed by streptavidin blotting. The anti FlaA/FlaB antibody[7] was used to detect the amount of flagellins in each sample.

A.

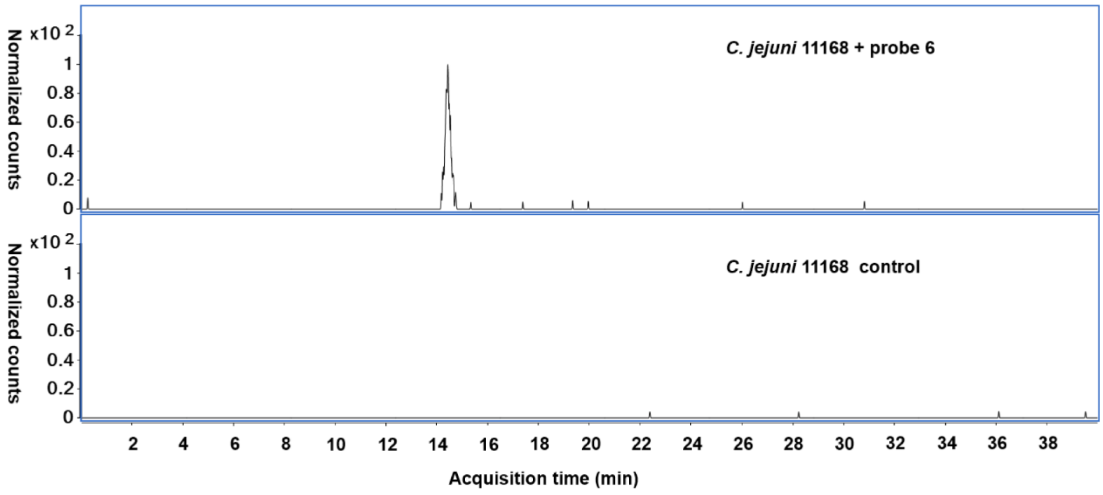

B.

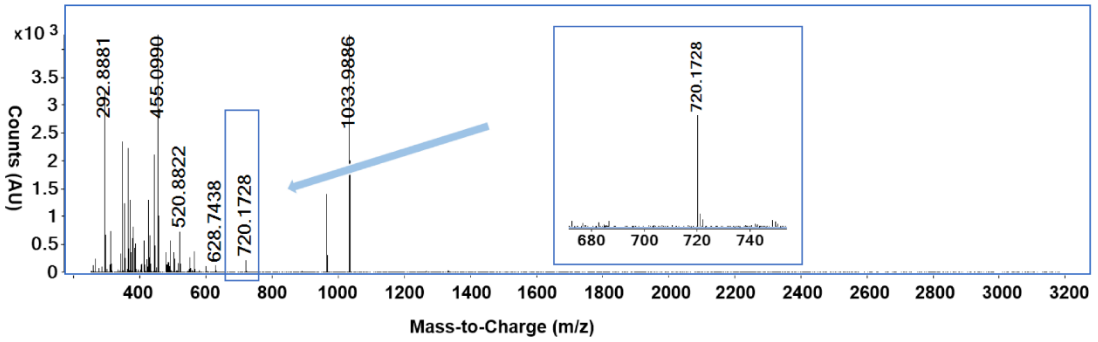

Figure S2. UPLC-MS extracted-ion chromatograms (EIC) analysis of CMP-LegdiNAz (m/z 720.1744) is shown for culture extracts from *C. jejuni* 11168, treated with 1 mM probe **6** and control group. A. The EIC for CMP-LegdiNAz. B. The mass spectrum (14.15-14.78 min, 158 scans).

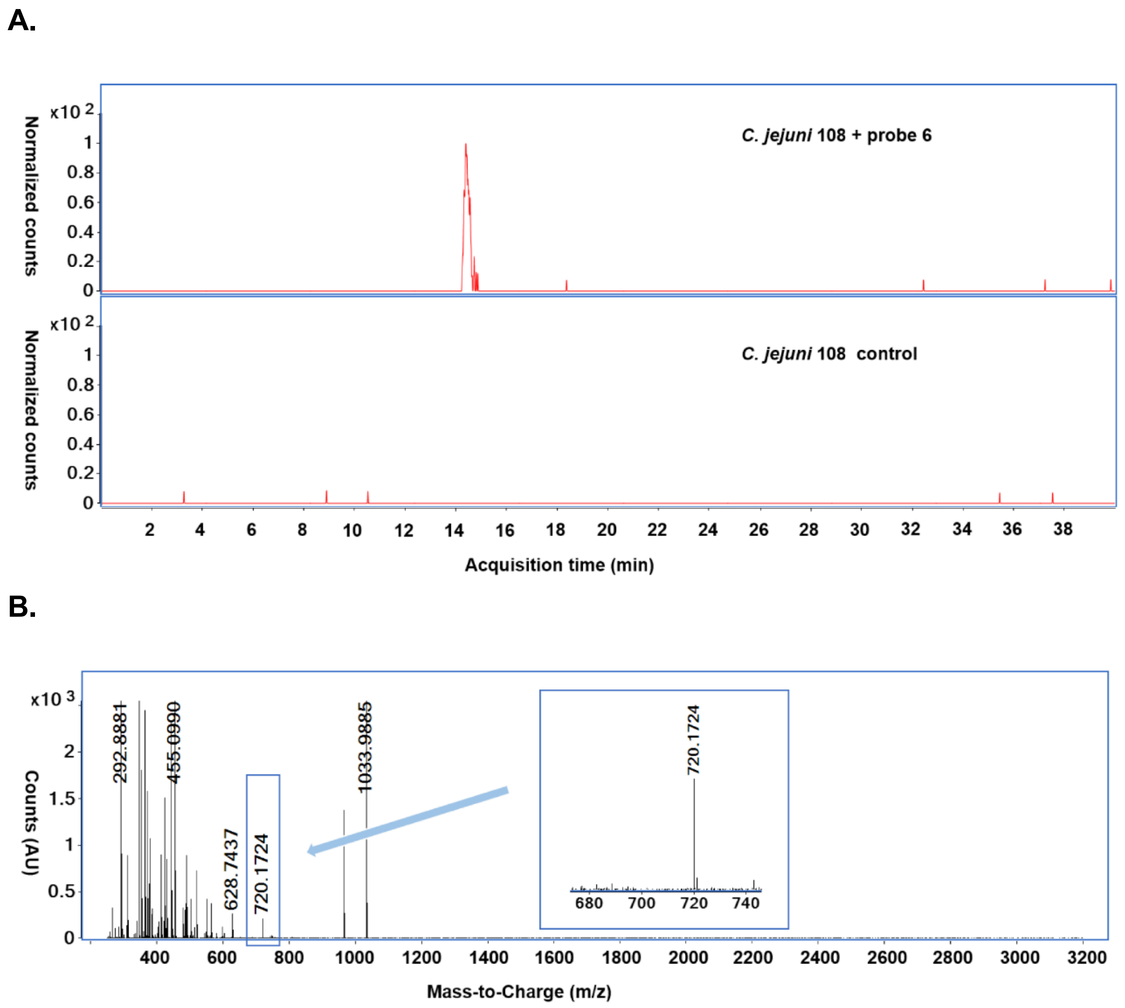

Figure S3. UPLC-MS extracted-ion chromatograms (EIC) analysis of CMP-LegdiNAz (m/z 720.1744) is shown for culture extracts from *C. jejuni* 108, treated with 1 mM probe **6** and control group. A. The EIC for CMP-LegdiNAz. B. The mass spectrum (14.22-14.86 min, 160 scans).

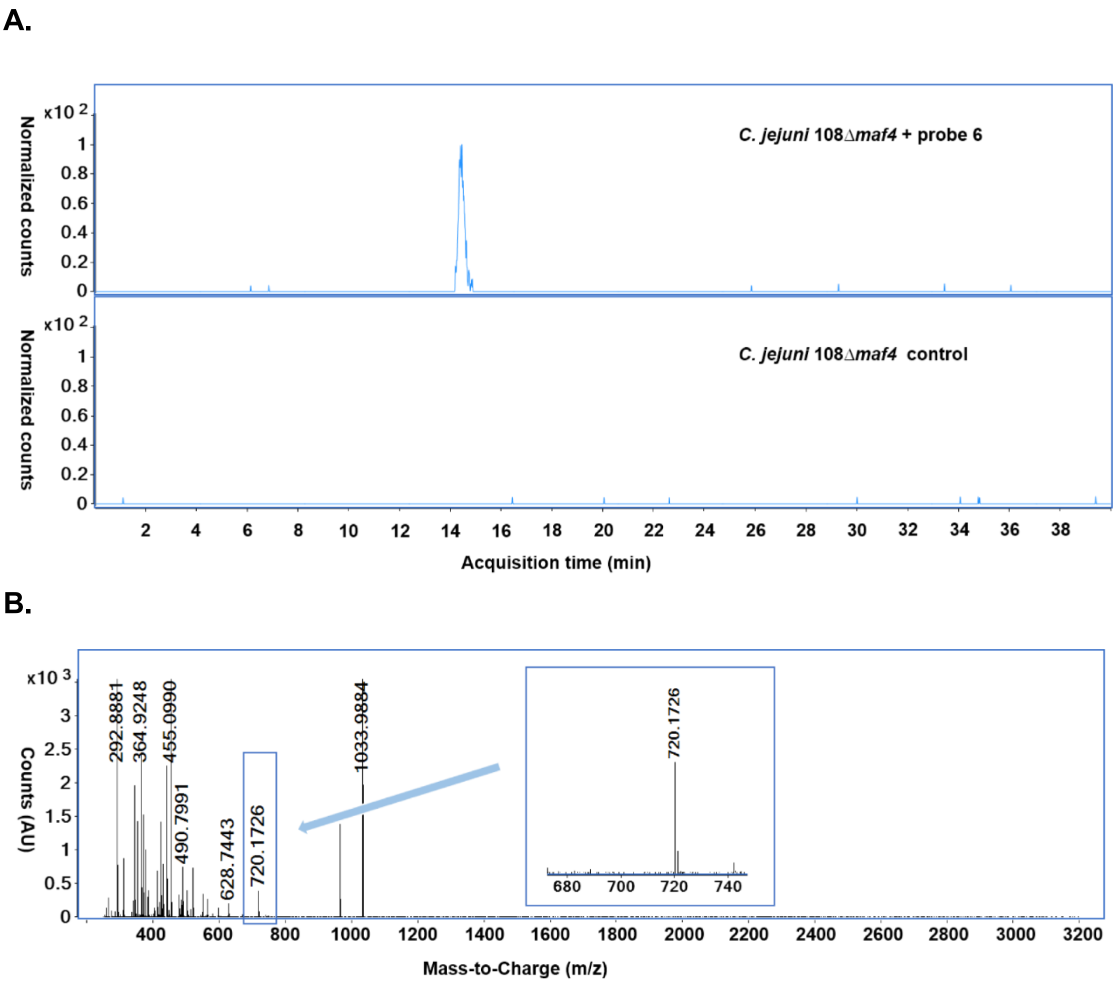

Figure S4. UPLC-MS extracted-ion chromatograms (EIC) analysis of CMP-LegdiNAz (m/z 720.1744) is shown for culture extracts from *C. jejuni* 108 *maf4*, treated with 1 mM probe **6** and control group. A. The EIC for CMP-LegdiNAz. B. The mass spectrum (14.20-14.86 min, 166 scans)

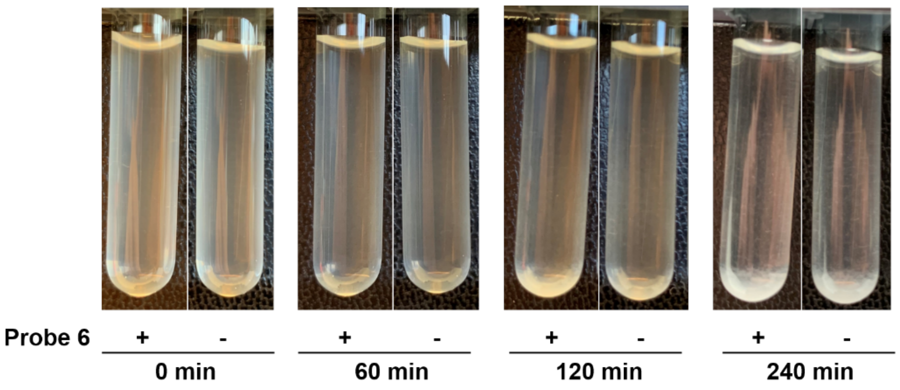

Figure S5. Autoagglutination at 0, 1, 2 and 4 h of incubation is depicted to illustrate the influence of probe **6** to *C.jejuni* 108 in kinetics of pellet formation.

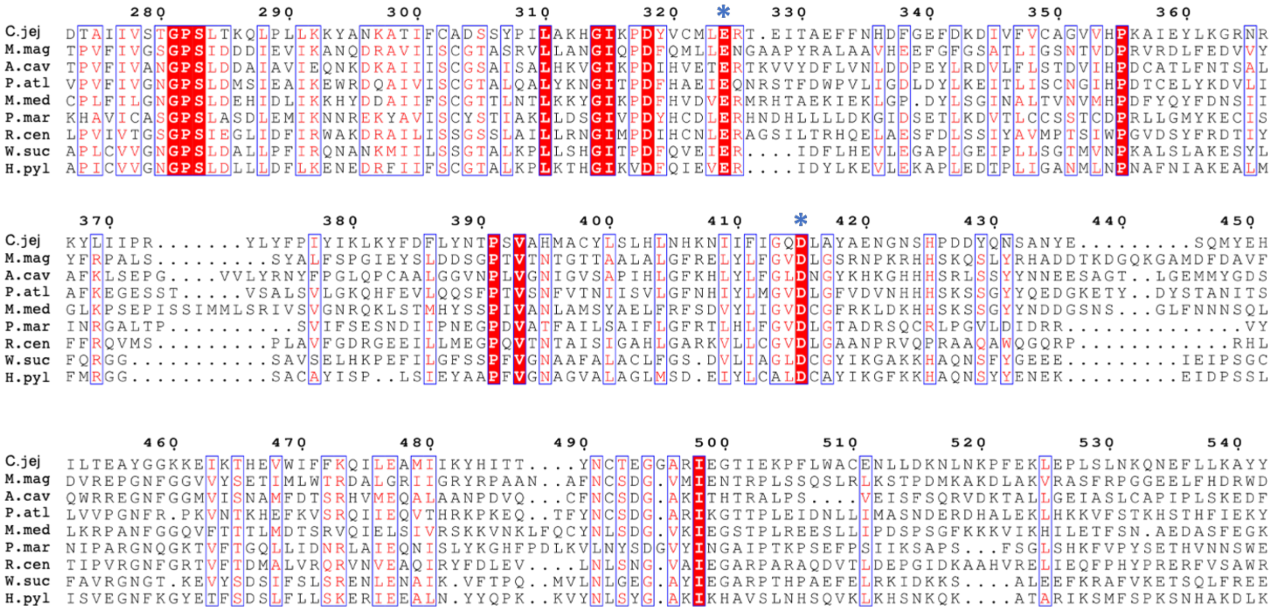

Figure S6 . Sequence alignment of putative legionaminyl transferase Maf4 (amino acids K239 to A457 central α/β domain) from *C.jejuni* 108 (C.jej) with homologous proteins from: M.mag, *Magnetospirillum magneticum* (NCBI accession WP_011383128.1) (used as standard); A.cav, *Aeromonas caviae* (WP_042015967.1); P.atl, *Pseudoalteromonas atlantica* (WP_011575799.1); M.med, *Marinomonas mediterranea* (WP_013662468.1); P.mar, *Prochlorococcus marinus* (WP_011129470.1); R.cen, *Rhodospirillum centenum* (WP_012568882.1); W.suc, *Wolinella succinogenes* (WP_011139562.1); H.pyl, *Helicobacter pylori* (WP_001116302.1). The putative catalytic residues are marked with blue asterisks.

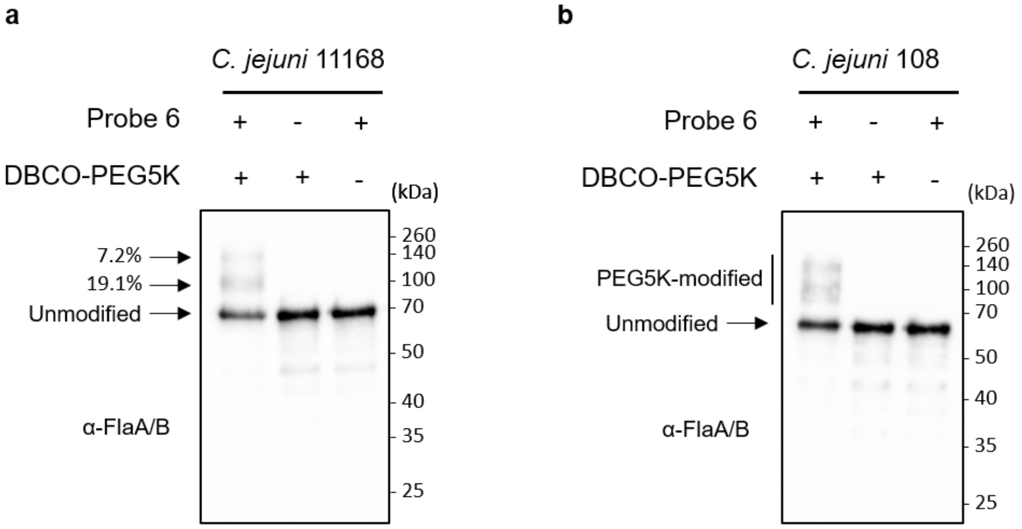

Figure S7. PEG mass shift allows visualization of metabolic labeled fraction of flagellins. *C. jejuni* 11168 cells (a) or *C. jejuni* 108 cells (b) were grown in HI medium supplemented with 1 mM probe **6** respectively, then incubated with 1 mM DBCO-PEG5K for 4h in PBS and then lysed, and further analyzed by western blotting. For the control samples, either the probe **6** or DBCO-PEG5K was omitted during the labeling procedure. The anti FlaA/FlaB antibody was used to detect the flagellin in each sample. The modified fraction of protein run at higher molecular weights due to PEGylation. The indicated stoichiometries were determined by measuring the relative intensities of the modified and unmodified bands.

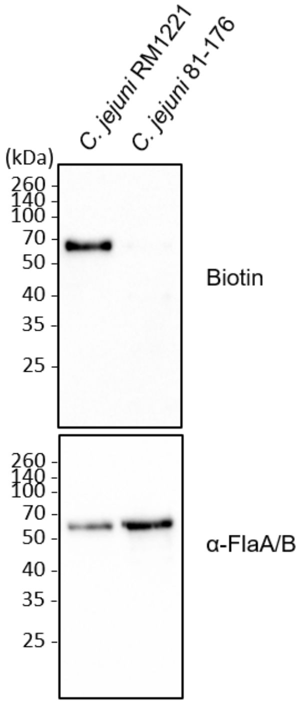

Figure S8. Western blot analysis of *C. jejuni* flagellins. *C. jejuni* RM1221 and *C. jejuni* 81-176 cells were grown in HI medium supplemented with 1 mM probe **6** for 4 h respectively, then incubated with 10 μM DBCO-PEG$_4$-Biotin for 1h in PBS and then lysed, and further analyzed by streptavidin blotting. The anti FlaA/FlaB antibody was used to detect the amount of flagellin in each sample.

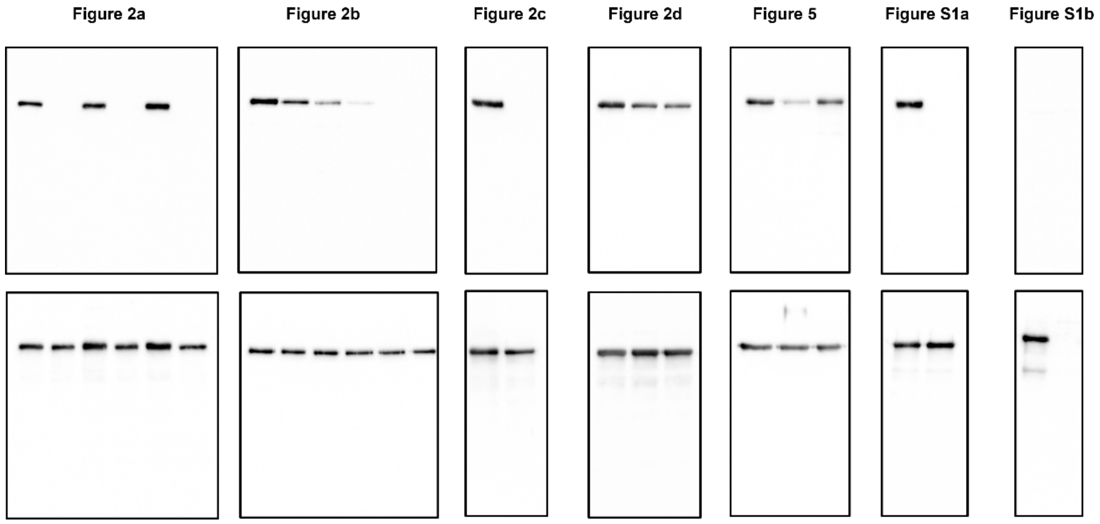

Figure S9. Full blots from corresponding Figures and Supplementary Figures. For each figure: The upper panel shows blots detected by Streptavidin-HRP; The panel below shows blots detected by the anti FlaA/FlaB antibody.

4. References for Supporting Information

[1] J. Parkhill, B. Wren, K. Mungall, J. Ketley, C. Churcher, D. Basham, T. Chillingworth, R. Davies, T. Feltwell, S. Holroyd, *Nature* **2000**, *403*, 665-668.

[2] H. Endtz, B. Giesendorf, A. Van Belkum, S. Lauwers, W. Jansen, W. Quint, *Res. Microbiol.* **1993**, *144*, 703-708.

[3] L. B. van Alphen, M. Wuhrer, N. M. Bleumink-Pluym, P. J. Hensbergen, A. M. Deelder, J. P. van Putten, *Microbiology* **2008**, *154*, 3385-3397.

[4] S. Palmer, P. Gully, J. White, A. Pearson, W. Suckling, D. Jones, J. Rawes, J. Penner, *Lancet* **1983**, *321*, 287-290.

[5] M. M. Wösten, L. Van Dijk, A. K. Veenendaal, M. R. De Zoete, N. M. Bleumink-Pluijm, J. P. Van Putten, *Mol. Microbiol.* **2010**, *75*, 1577-1591.

[6] A. van Mourik, N. M. Bleumink-Pluym, L. van Dijk, J. P. van Putten, M. M. Wösten, *Microbiology* **2008**, *154*, 584-592.

[7] P. Nuijten, N. Bleumink-Pluym, W. Gaastra, B. Van Der Zeijst, *Infect. Immun.* **1989**, *57*, 1084-1088.

[8] W. G. Miller, A. H. Bates, S. T. Horn, M. T. Brandl, M. R. Wachtel, R. E. Mandrell, *Appl. Microbiol. Biotechnol.* **2000**, *66,* 5426-5436.

[9] J. A. Korlath, M. T. Osterholm, L. A. Judy, J. C. Forfang, R. A. Robinson, *J. Infect. Dis.* **1985**, *152,* 592-596.

5. NMR spectrum

^{1}H NMR spectrum of 13 (600 MHz, $CDCl_3$)

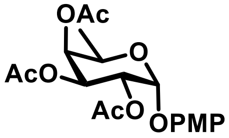

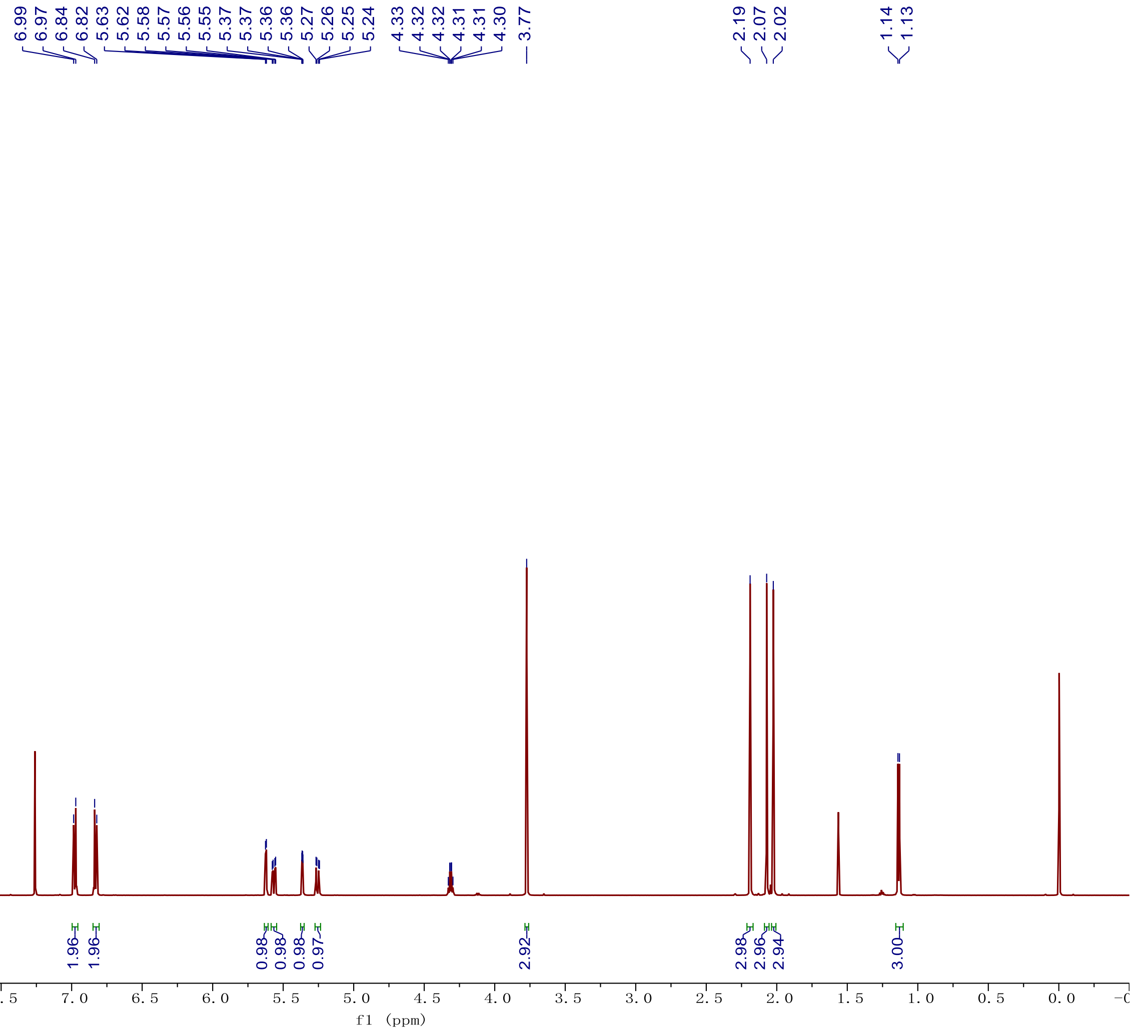

^{13}C NMR spectrum of 13 (151 MHz, CDCl3)

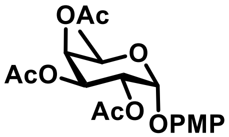

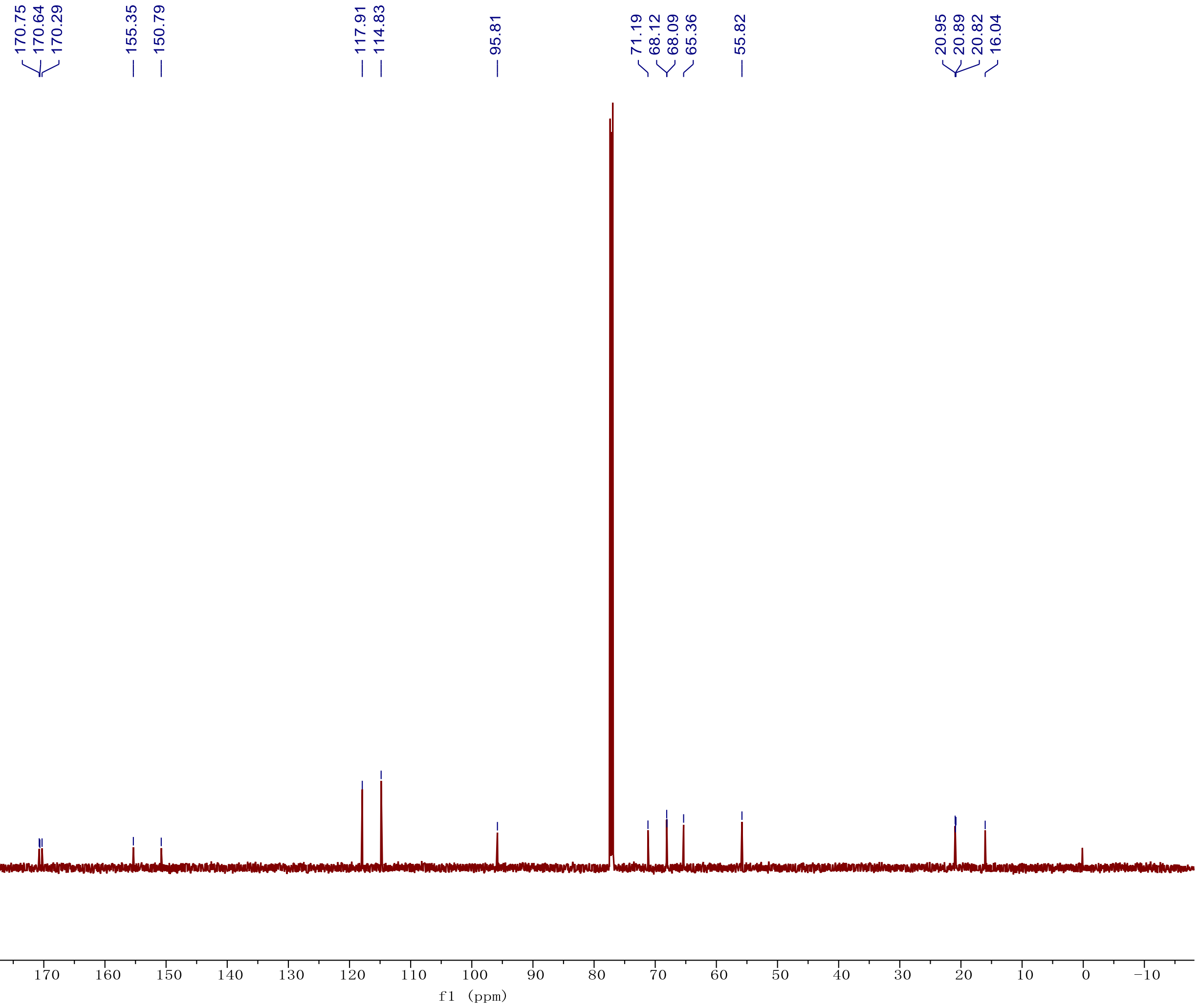

^{1}H NMR spectrum of 14 (600 MHz, $CDCl_3$)

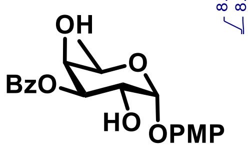

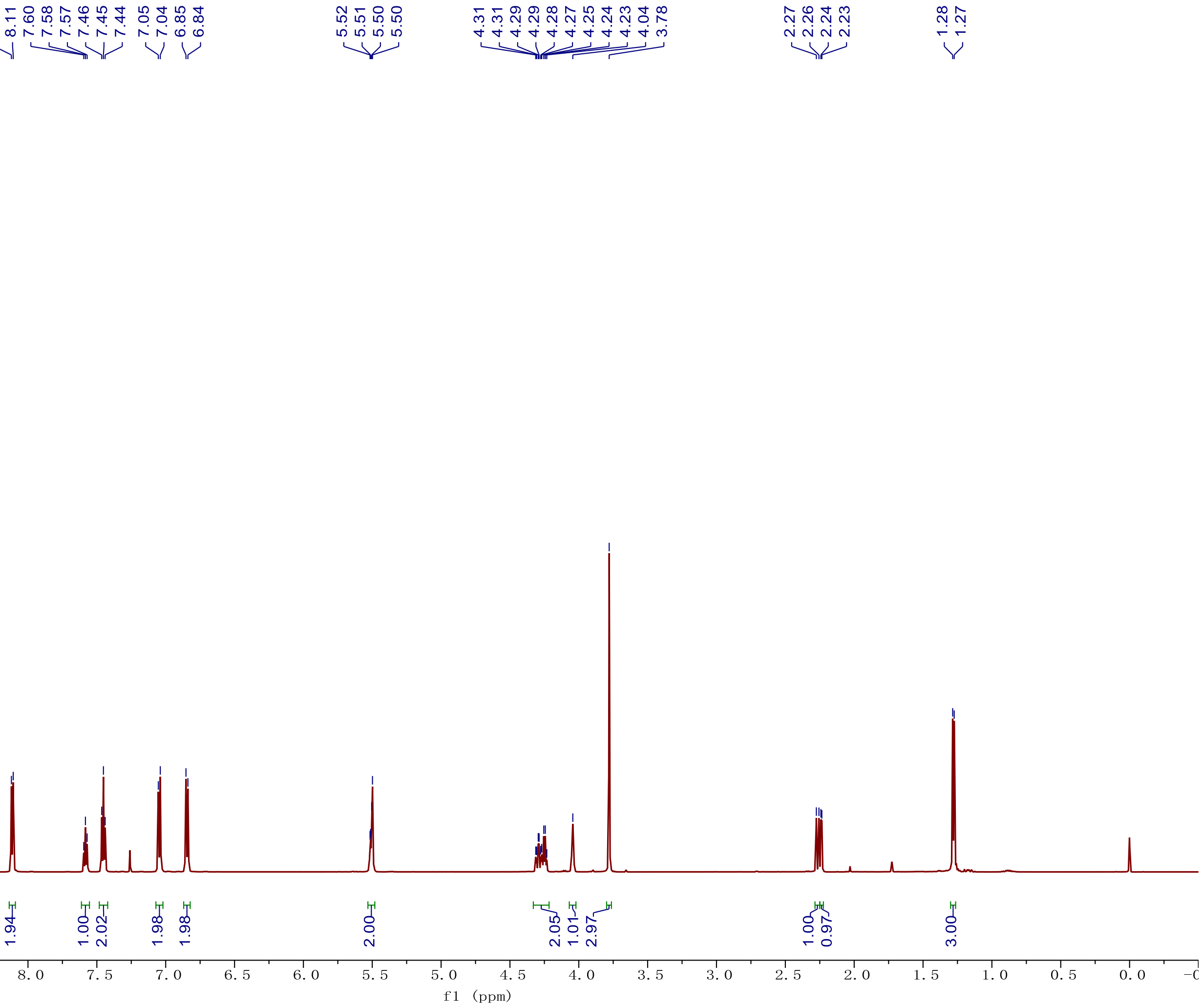

^{13}C NMR spectrum of 14 (151 MHz, CDCl₃)

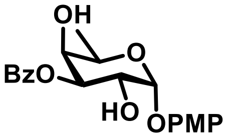

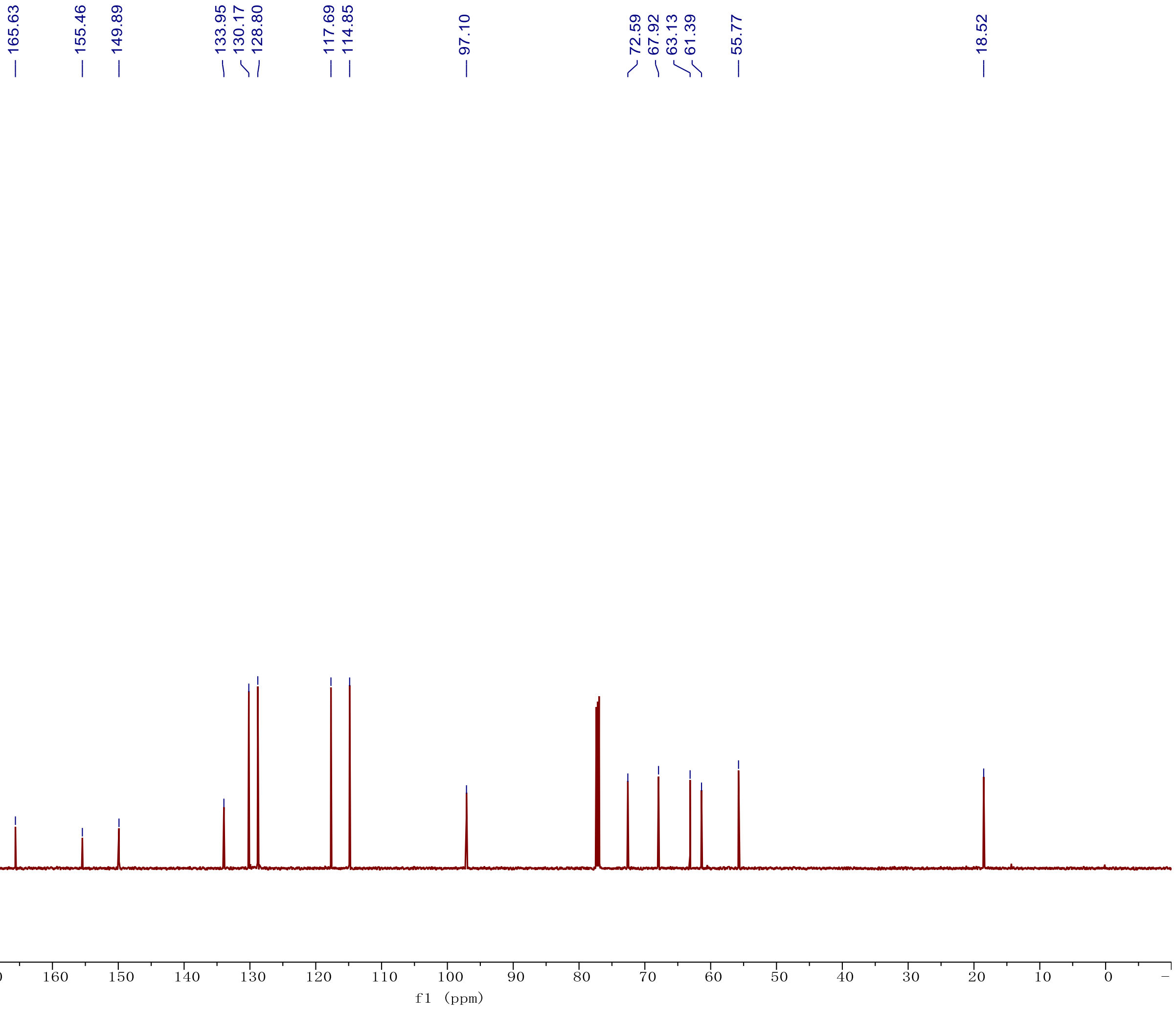

^{1}H NMR spectrum of 15 (600 MHz, $CDCl_3$)

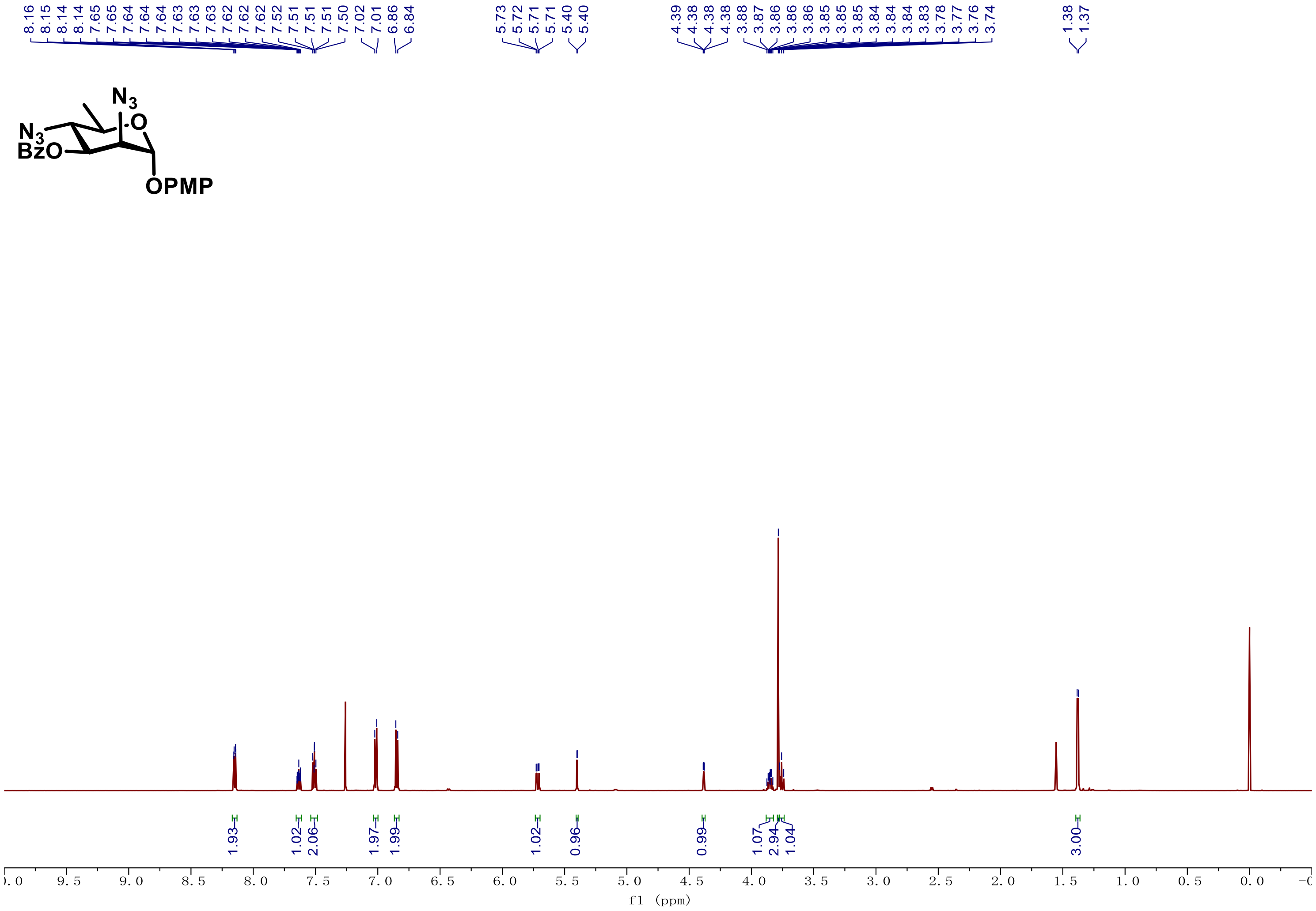

^{13}C NMR spectrum of 15 (151 MHz, CDCl$_3$)

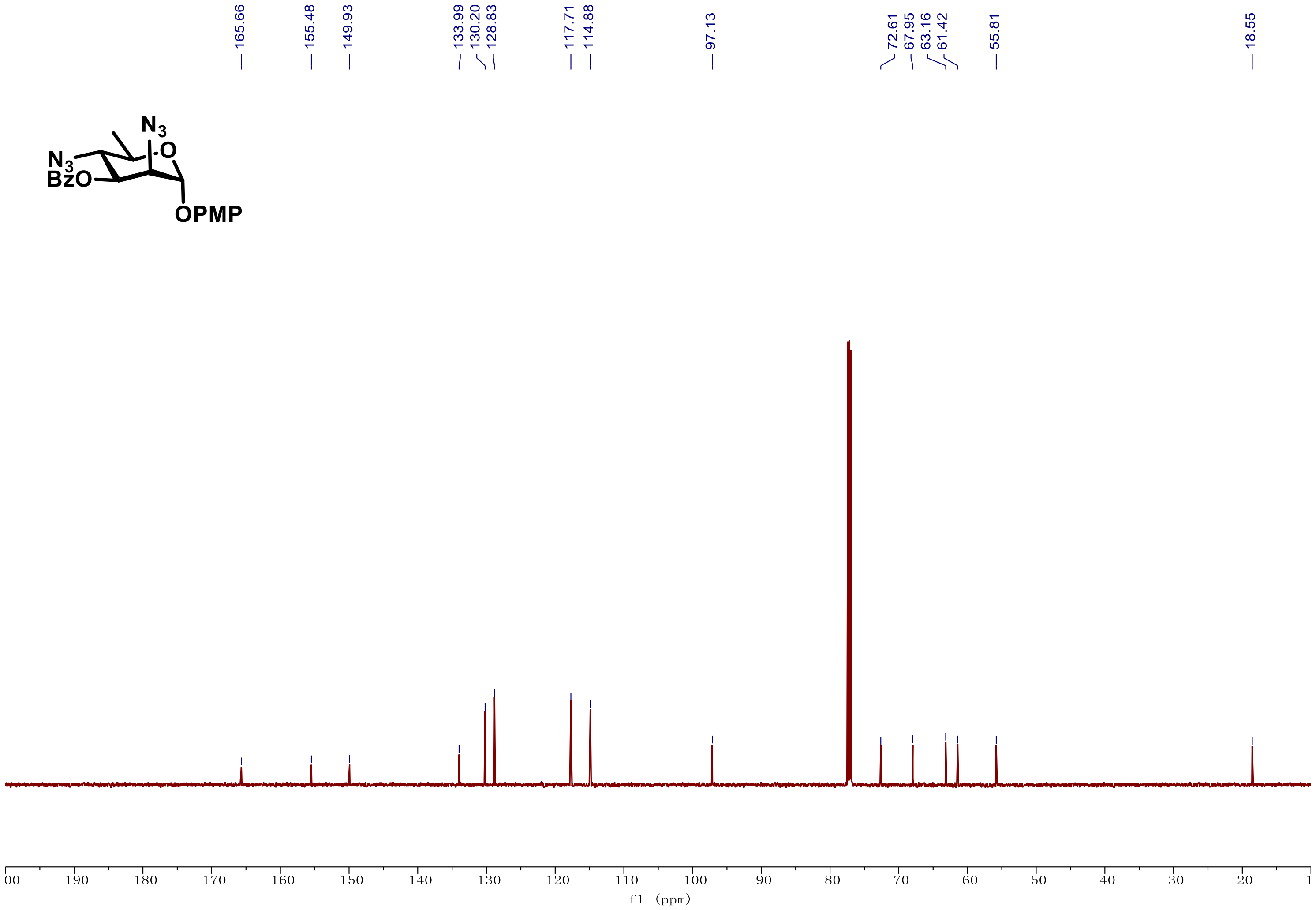

^{1}H NMR spectrum of 16 (600 MHz, $CDCl_3$)

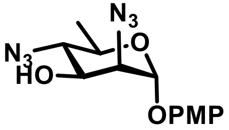

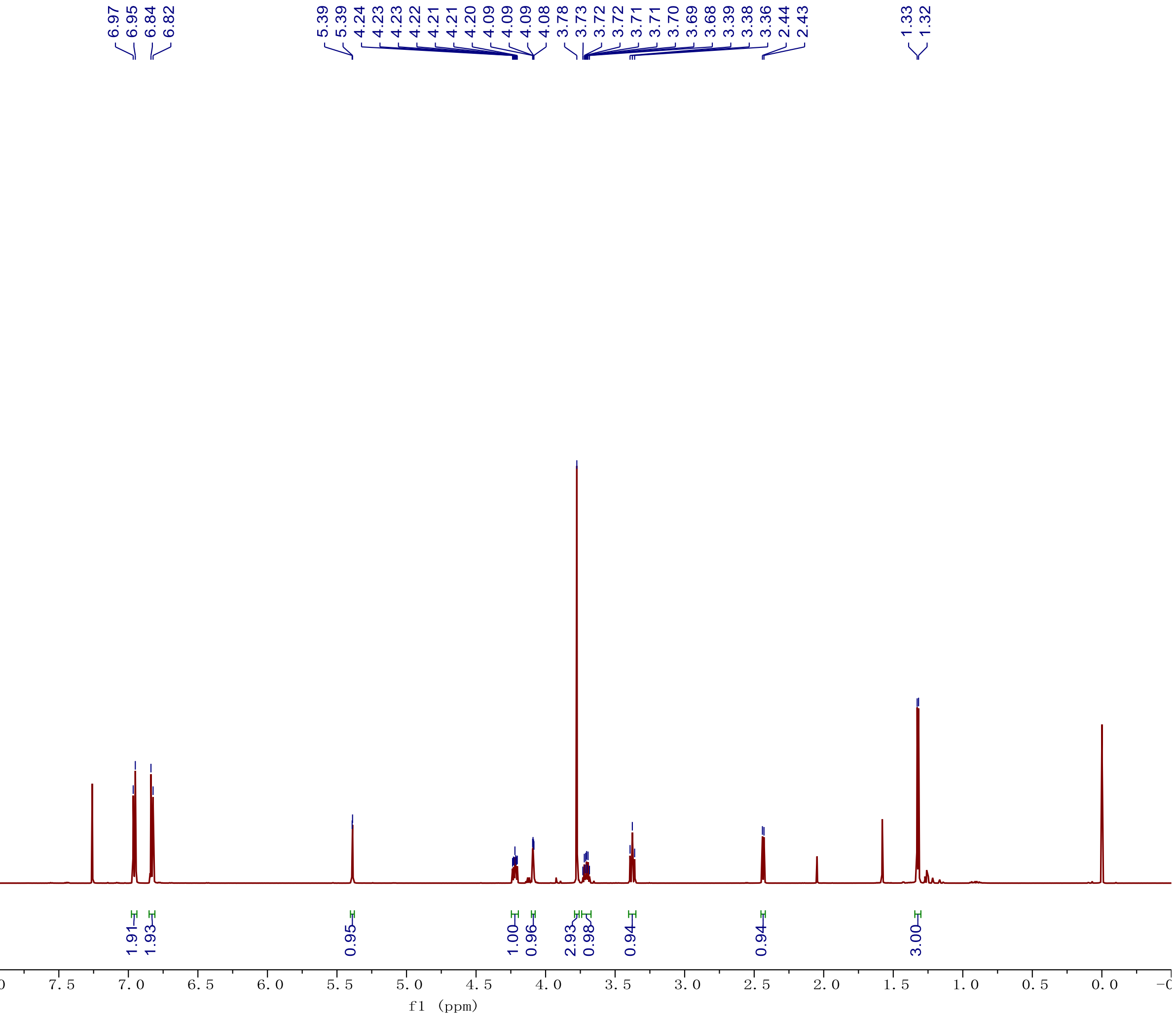

^{13}C NMR spectrum of 16 (151 MHz, $CDCl_3$)

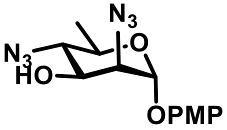

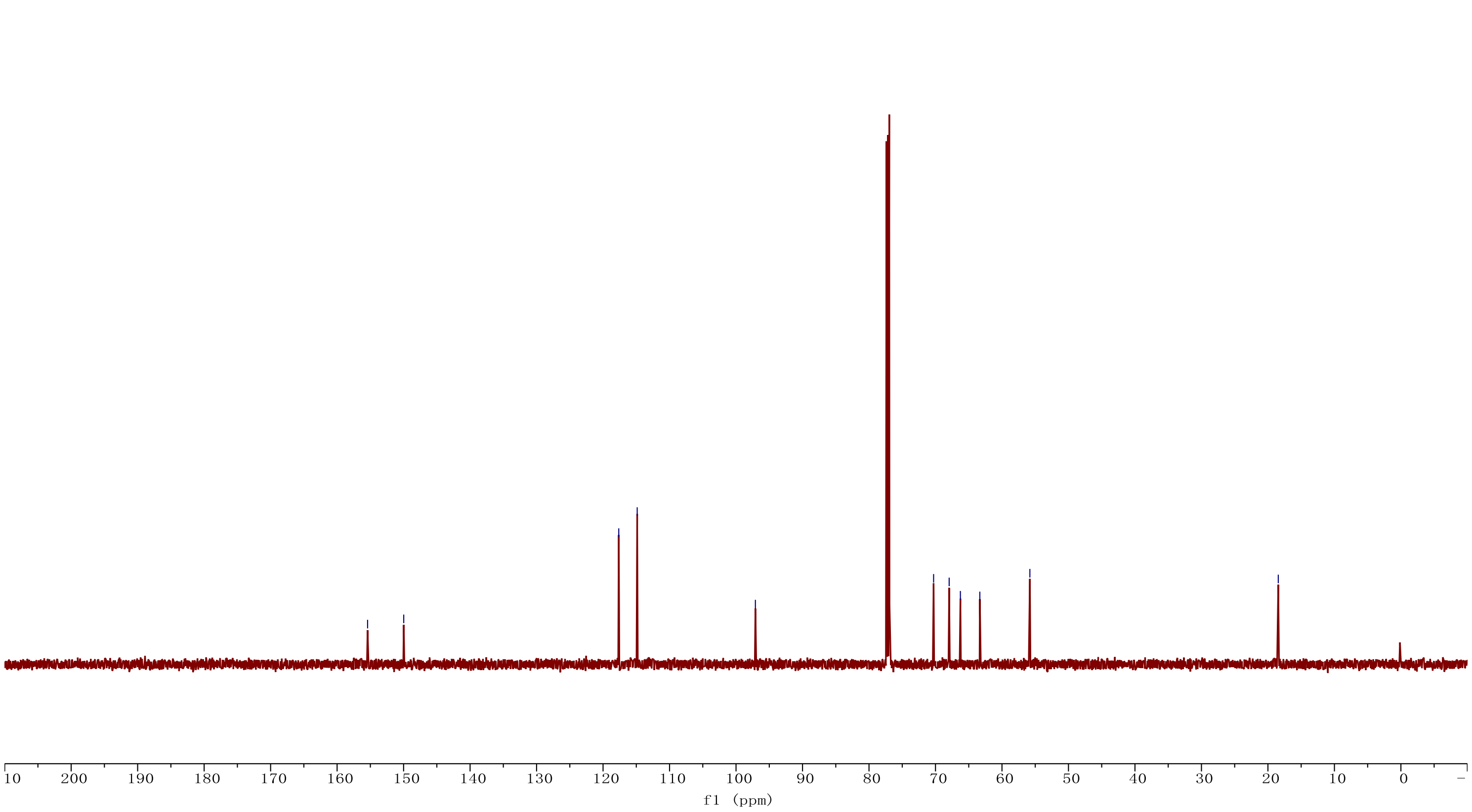

^{1}H NMR spectrum of 20 (600 MHz, CD_3OD)

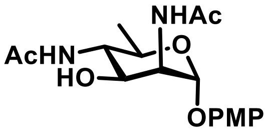

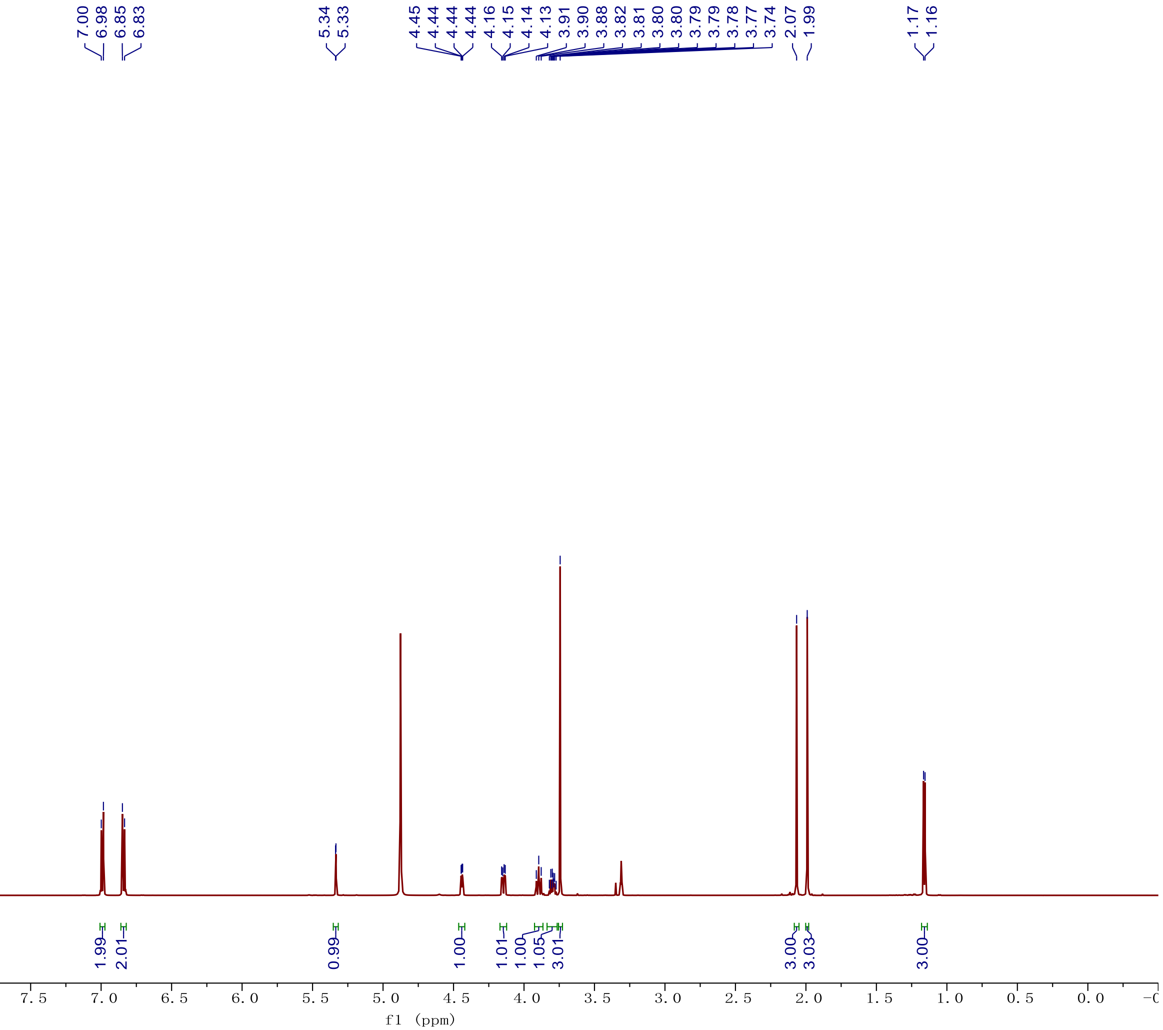

^{13}C NMR spectrum of 20 (151 MHz, CD_3OD)

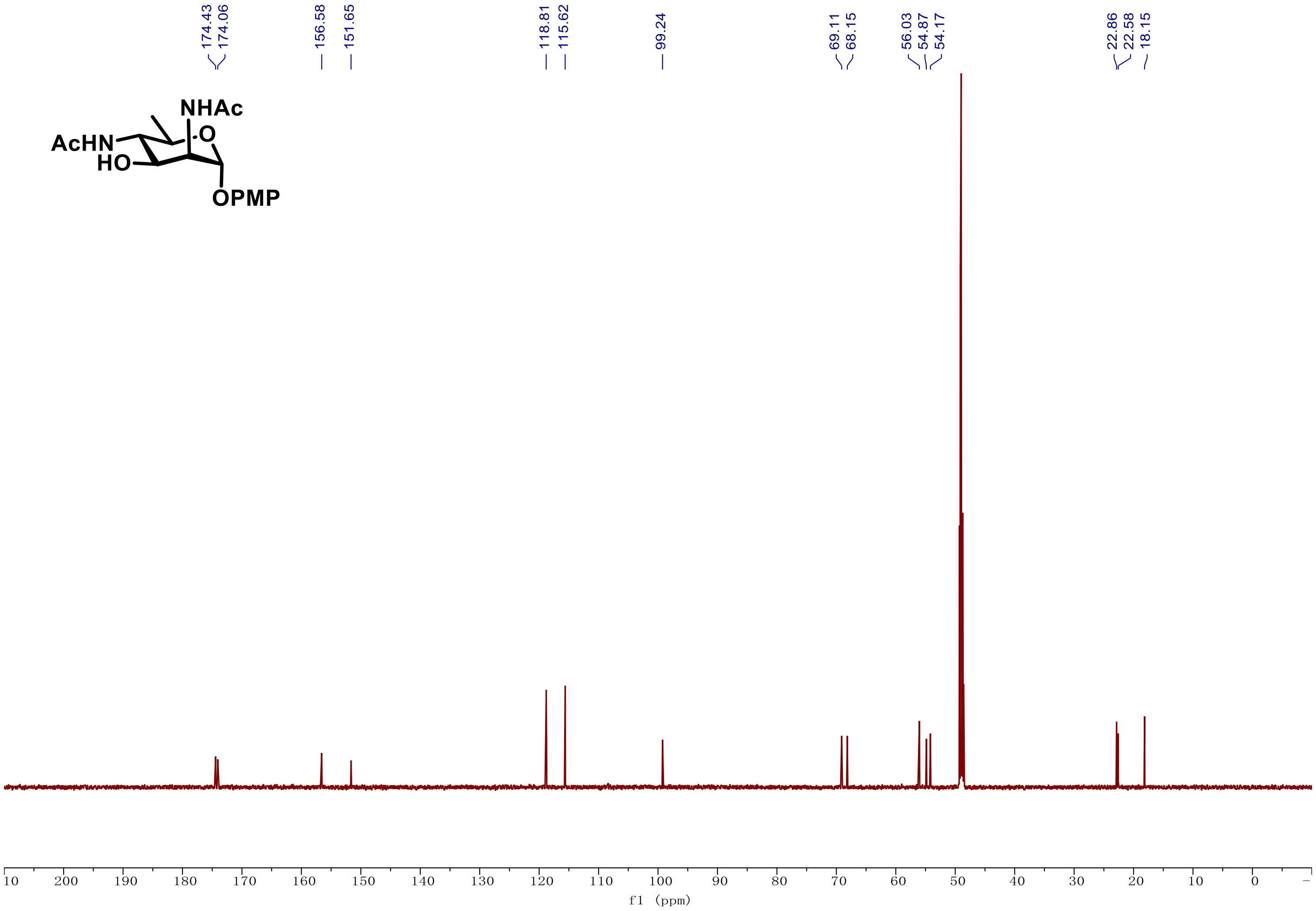

^{1}H NMR spectrum of 1 (600 MHz, D_2O)

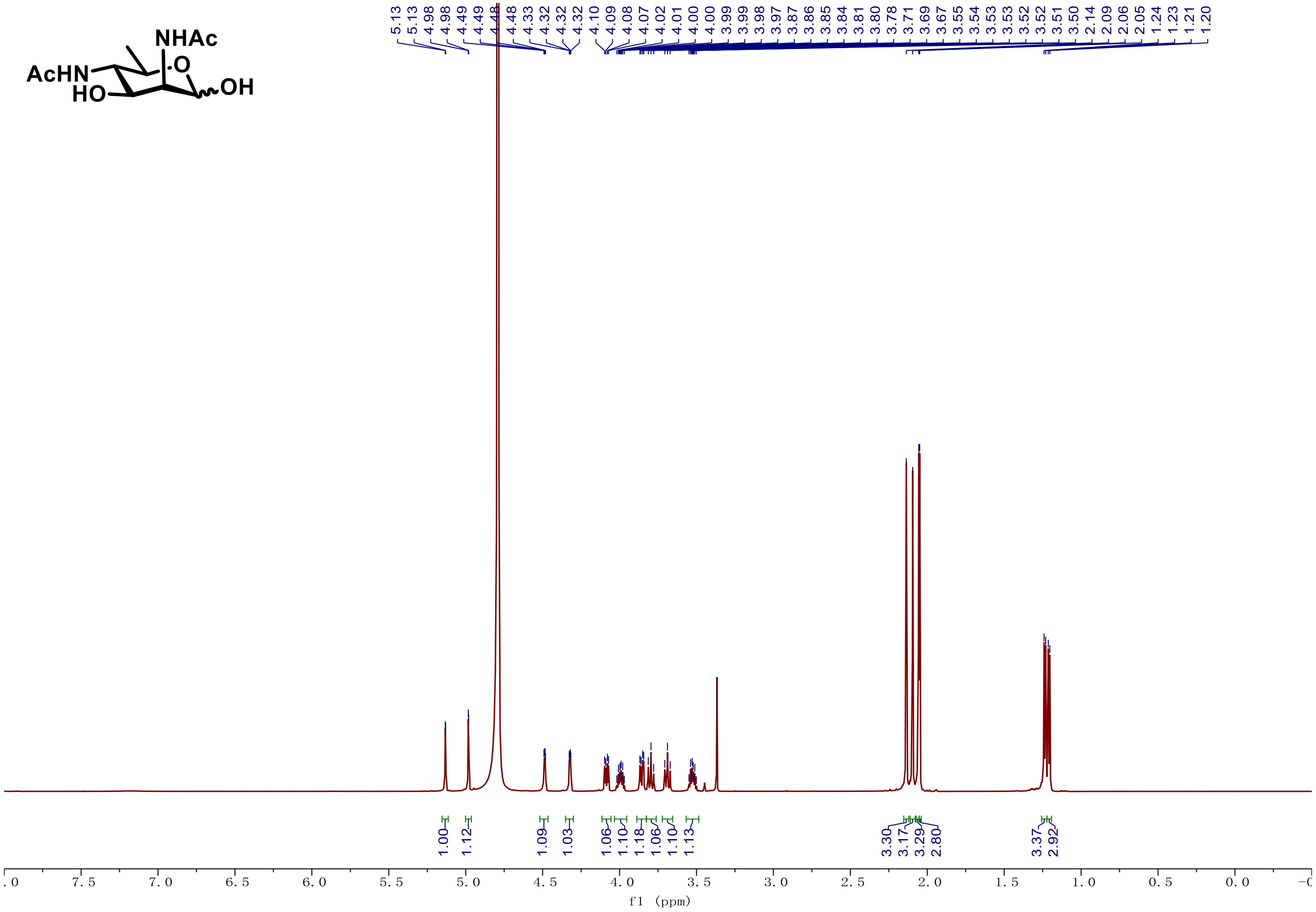

^{13}C NMR spectrum of 1 (151 MHz, D_2O)

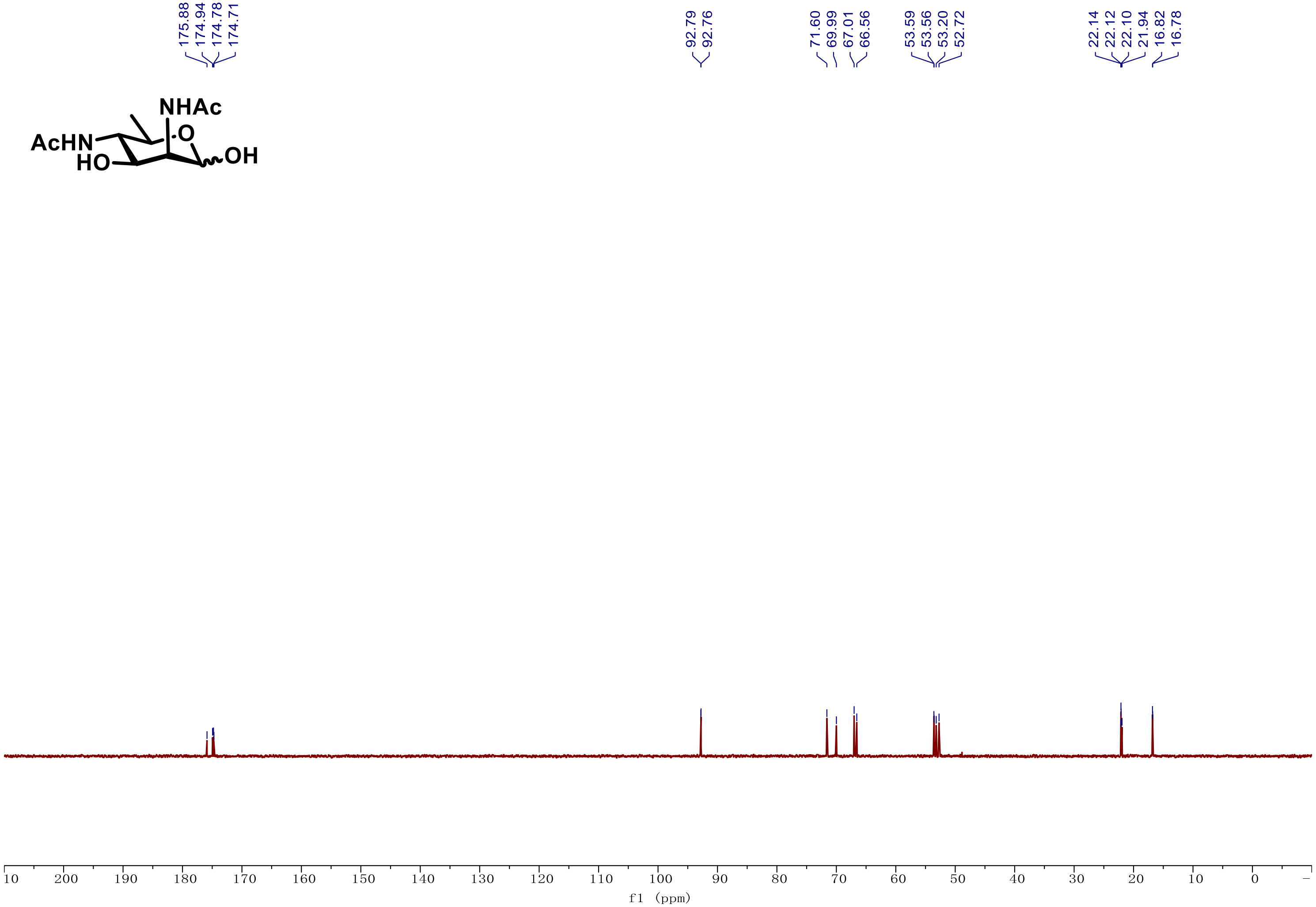

^{1}H NMR spectrum of S1 (600 MHz, CD_3OD)

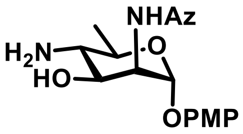

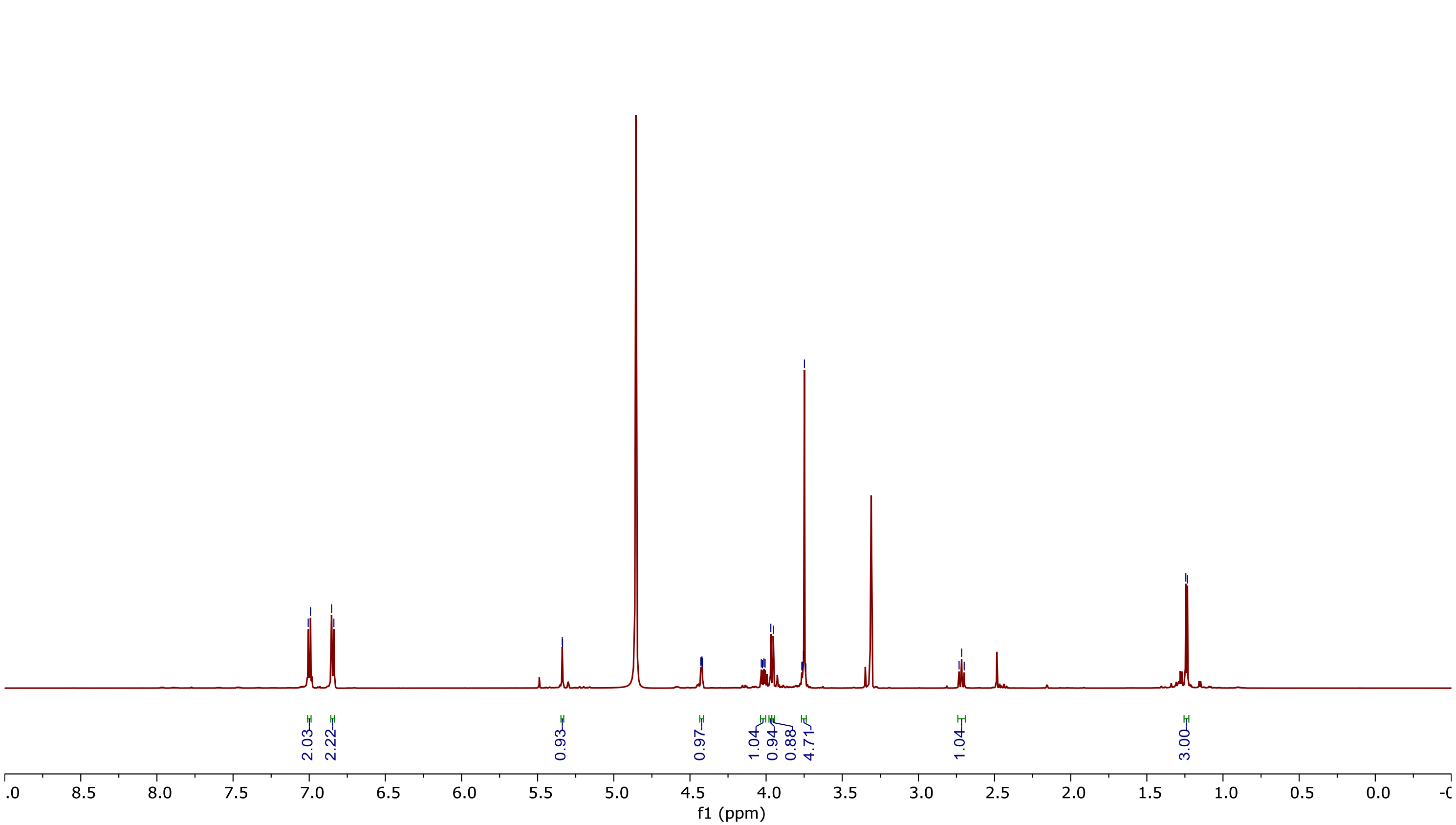

^{13}C NMR spectrum of S1 (151 MHz, CD_3OD)

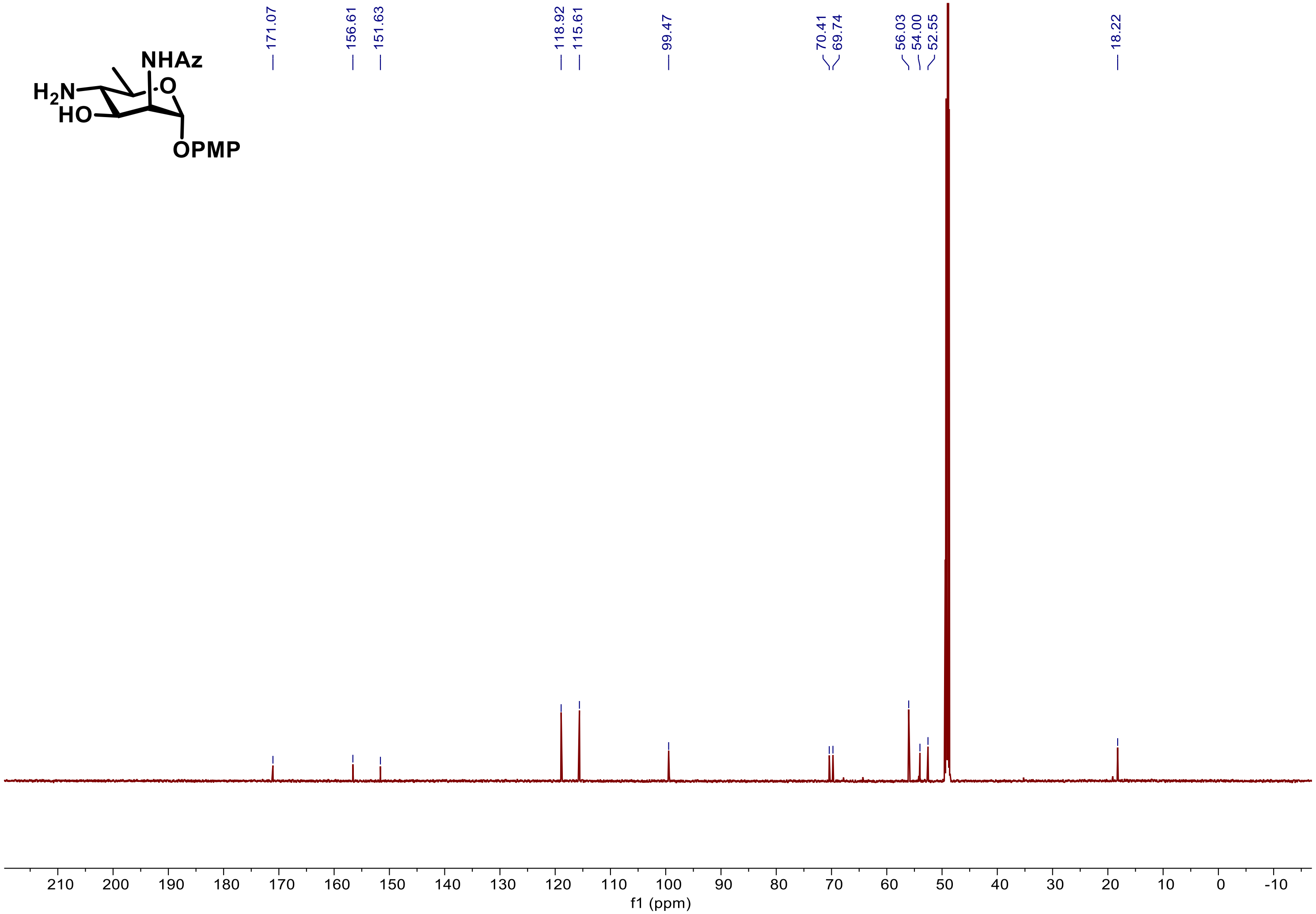

HSQC NMR spectrum of S1 (CD_3OD)

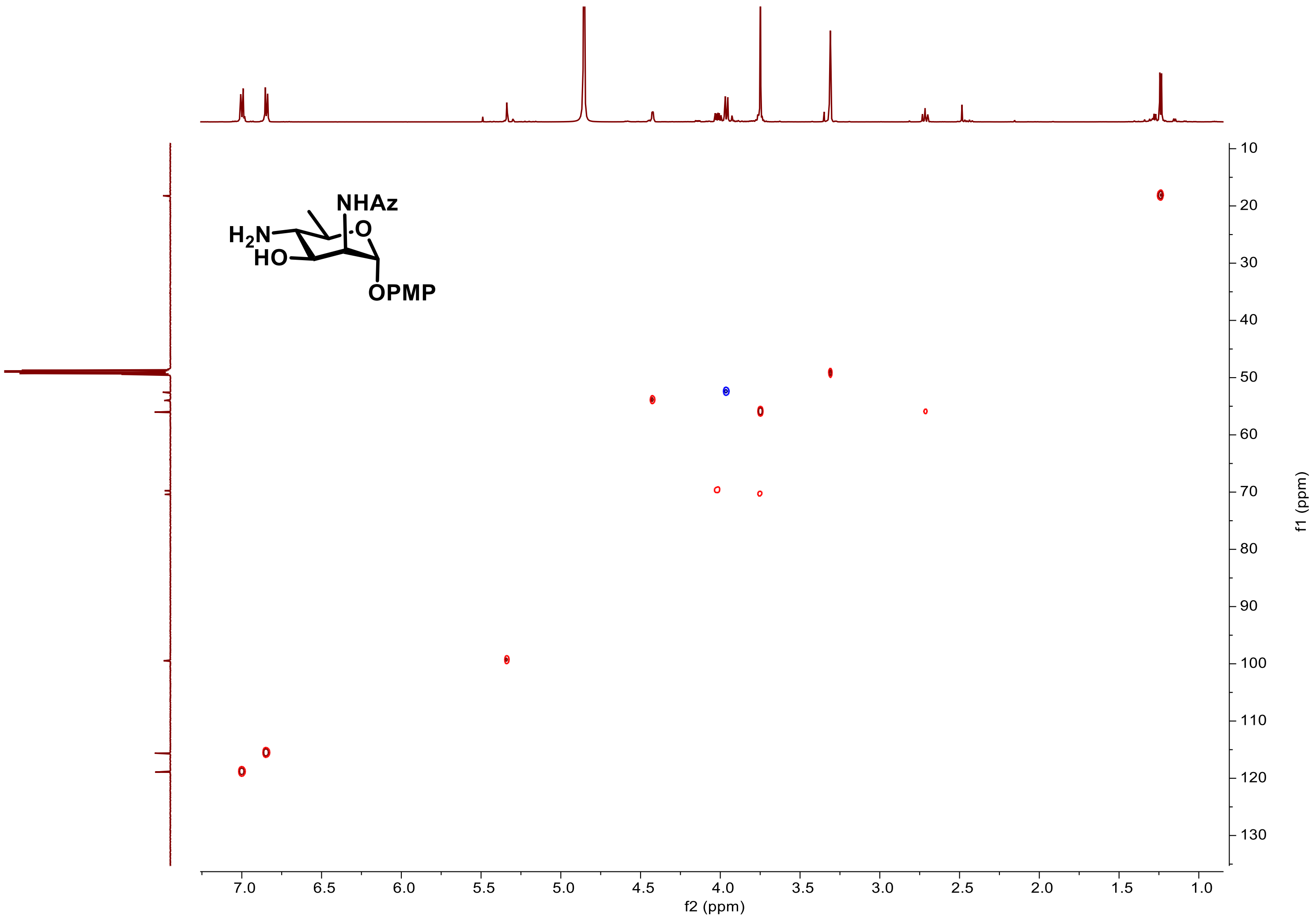

^{1}H NMR spectrum of 17 (600 MHz, CD_3OD)

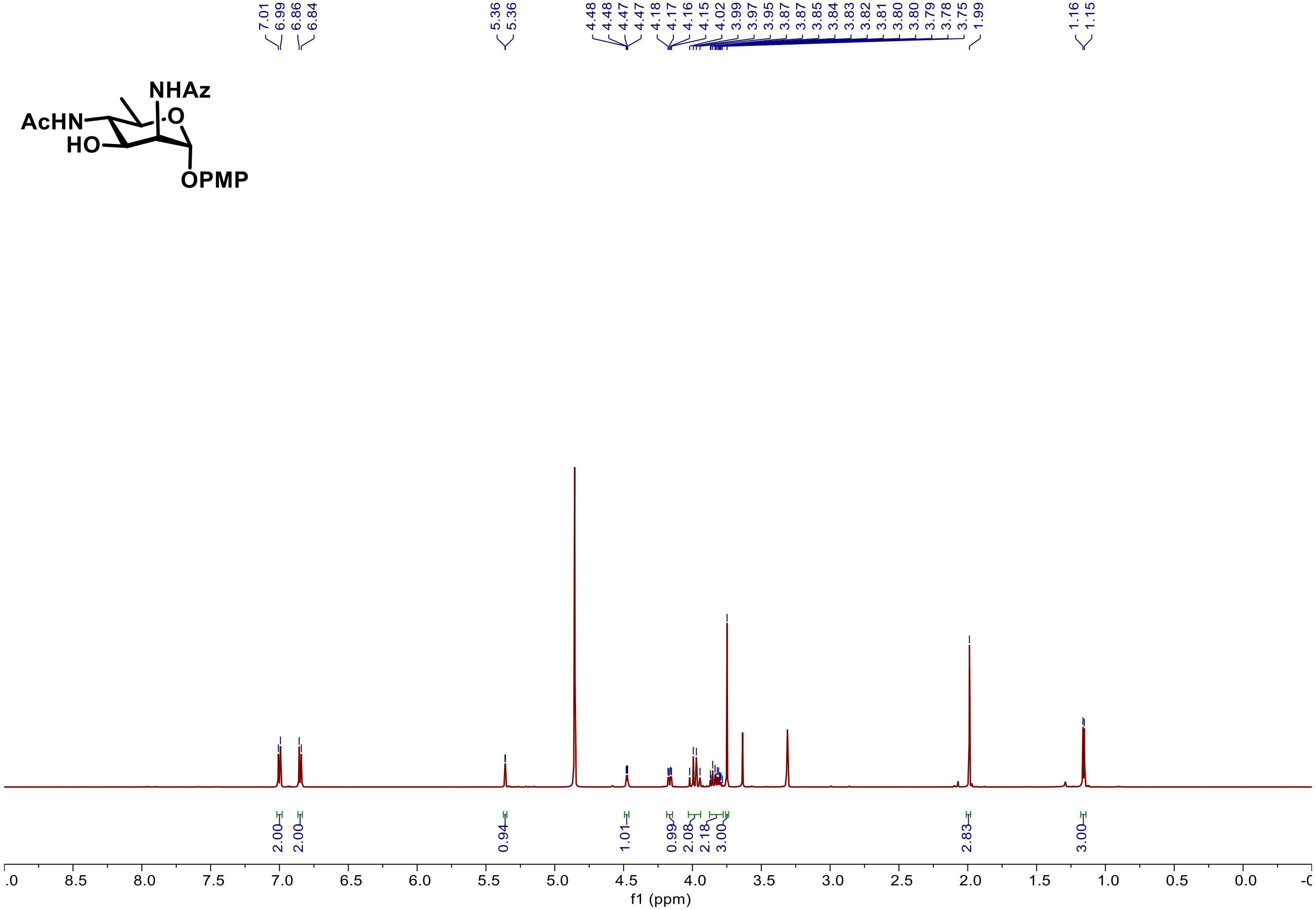

^{13}C NMR spectrum of 17 (151 MHz, CD_3OD)

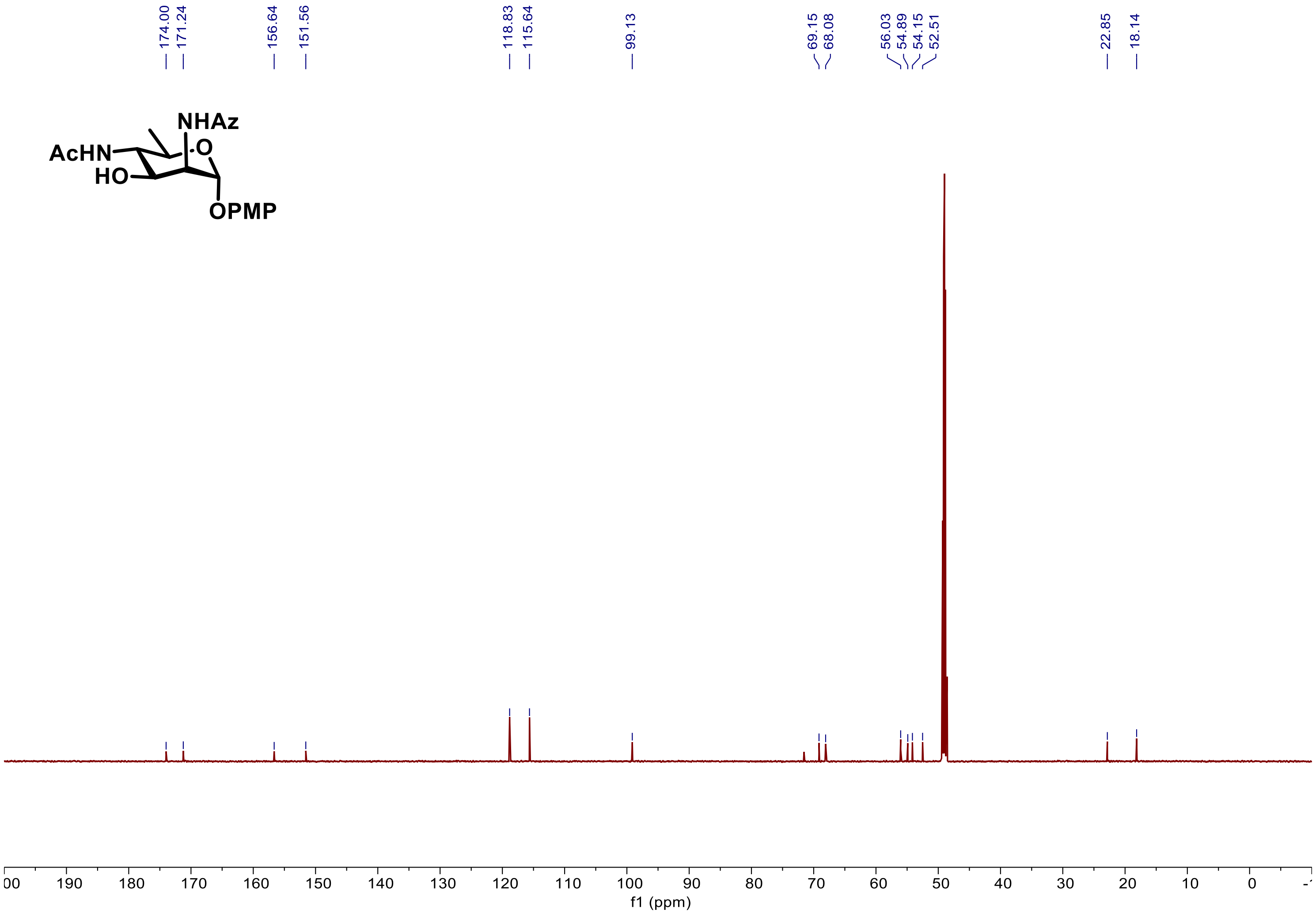

^{1}H NMR spectrum of 2 (600 MHz, CD_3OD)

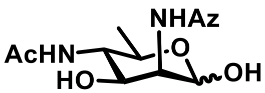

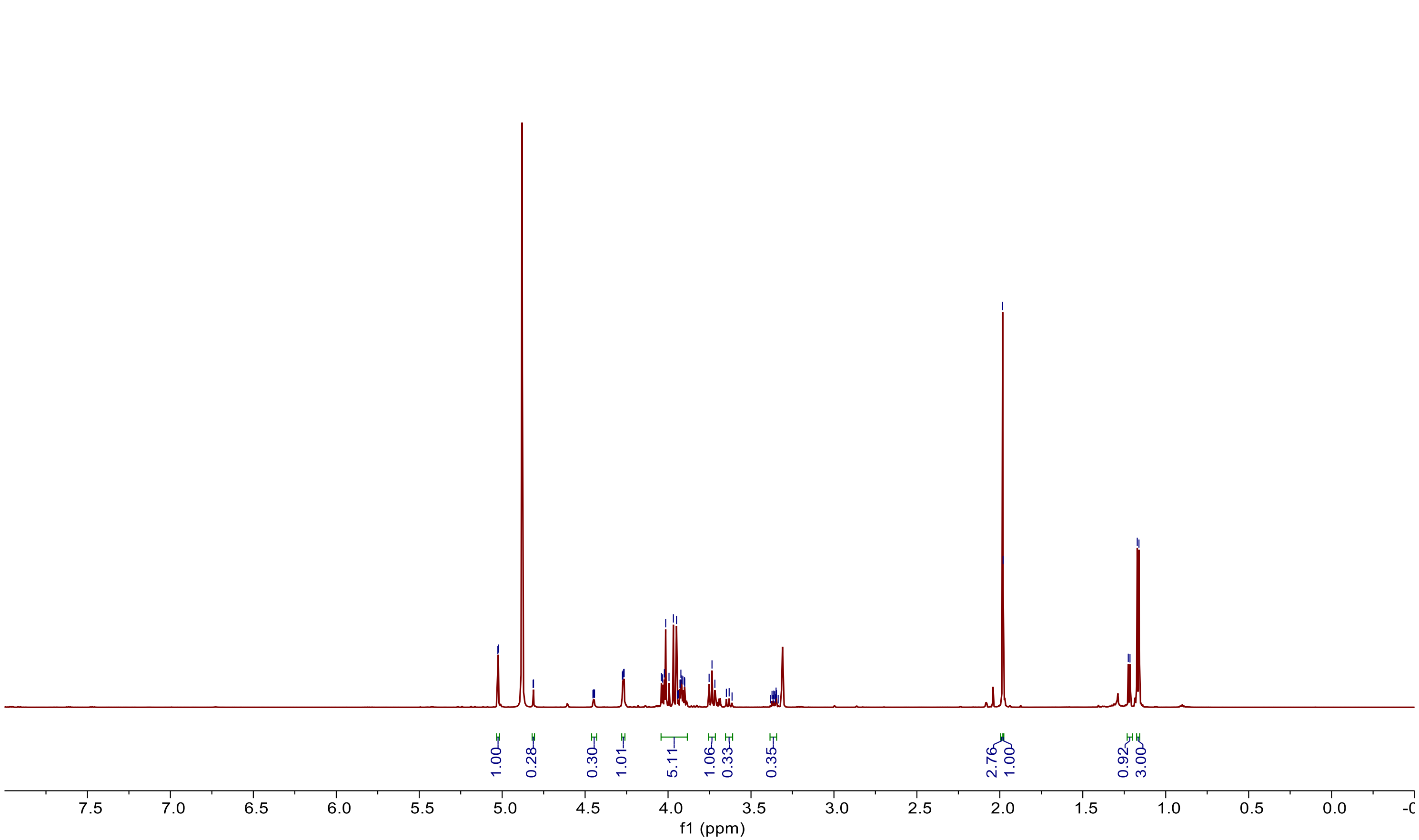

^{13}C NMR spectrum of 2 (151 MHz, CD_3OD)

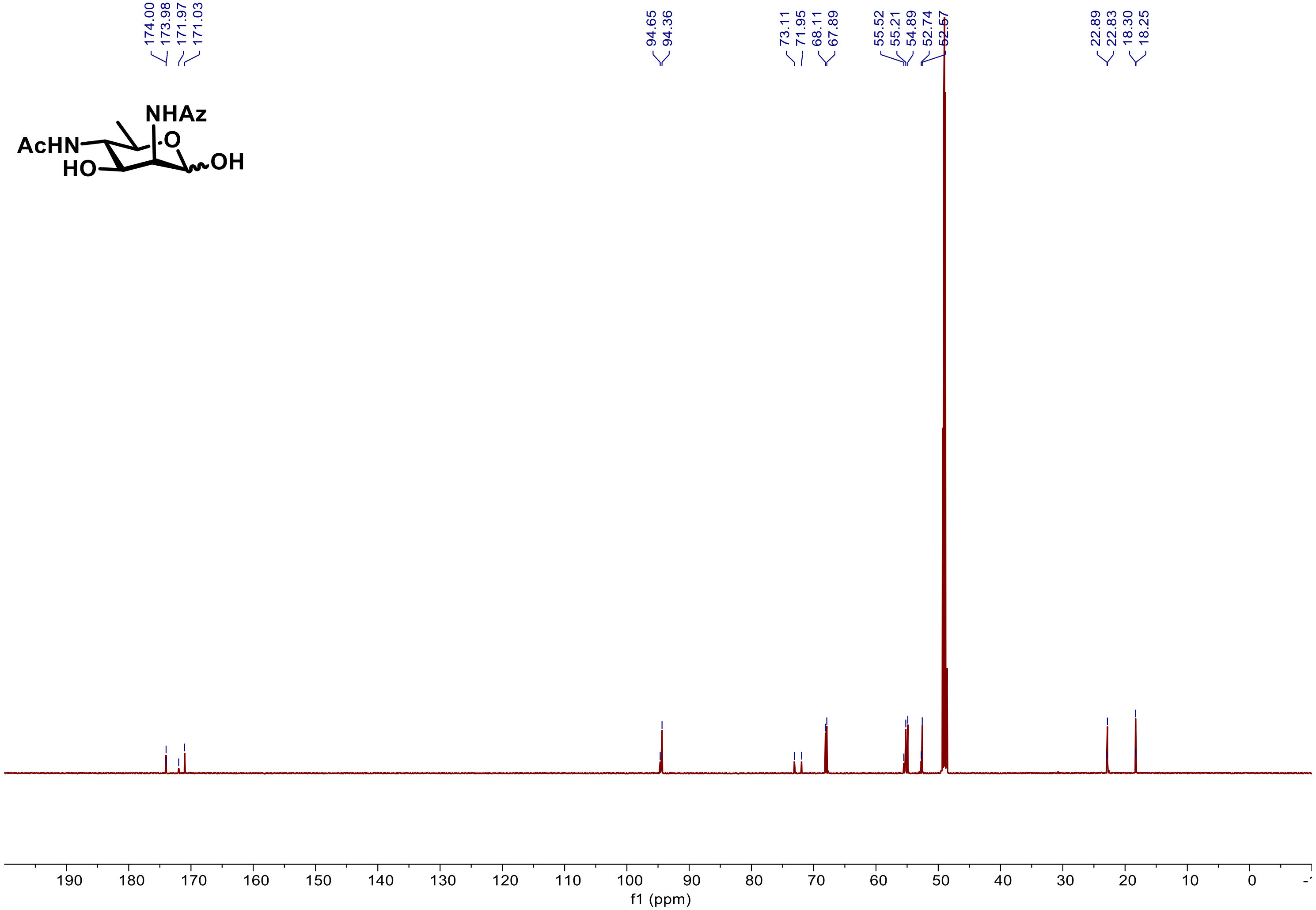

HSQC NMR spectrum of 2 (CD_3OD)

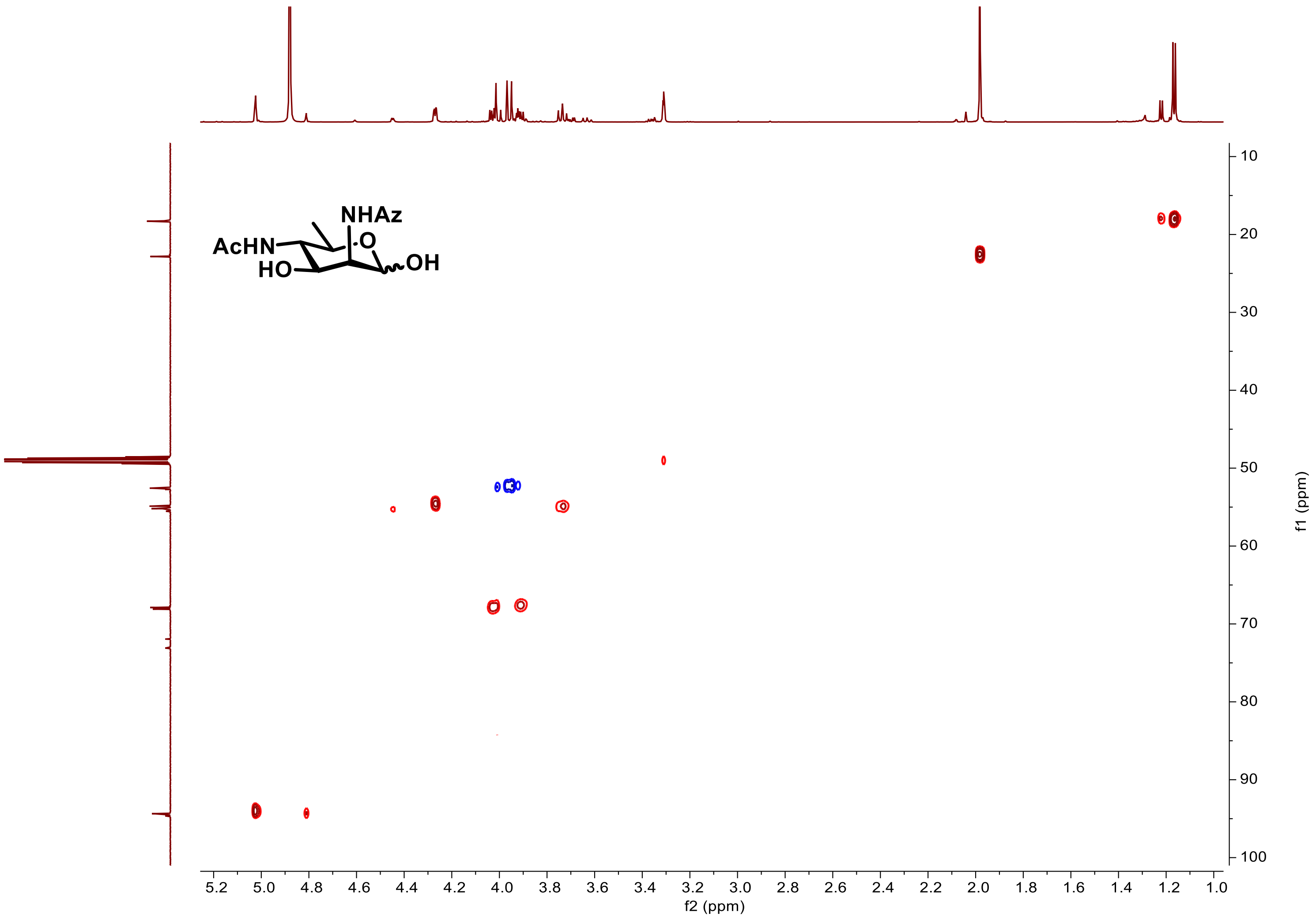

^{1}H NMR spectrum of 3 (600 MHz, CD_3OD)

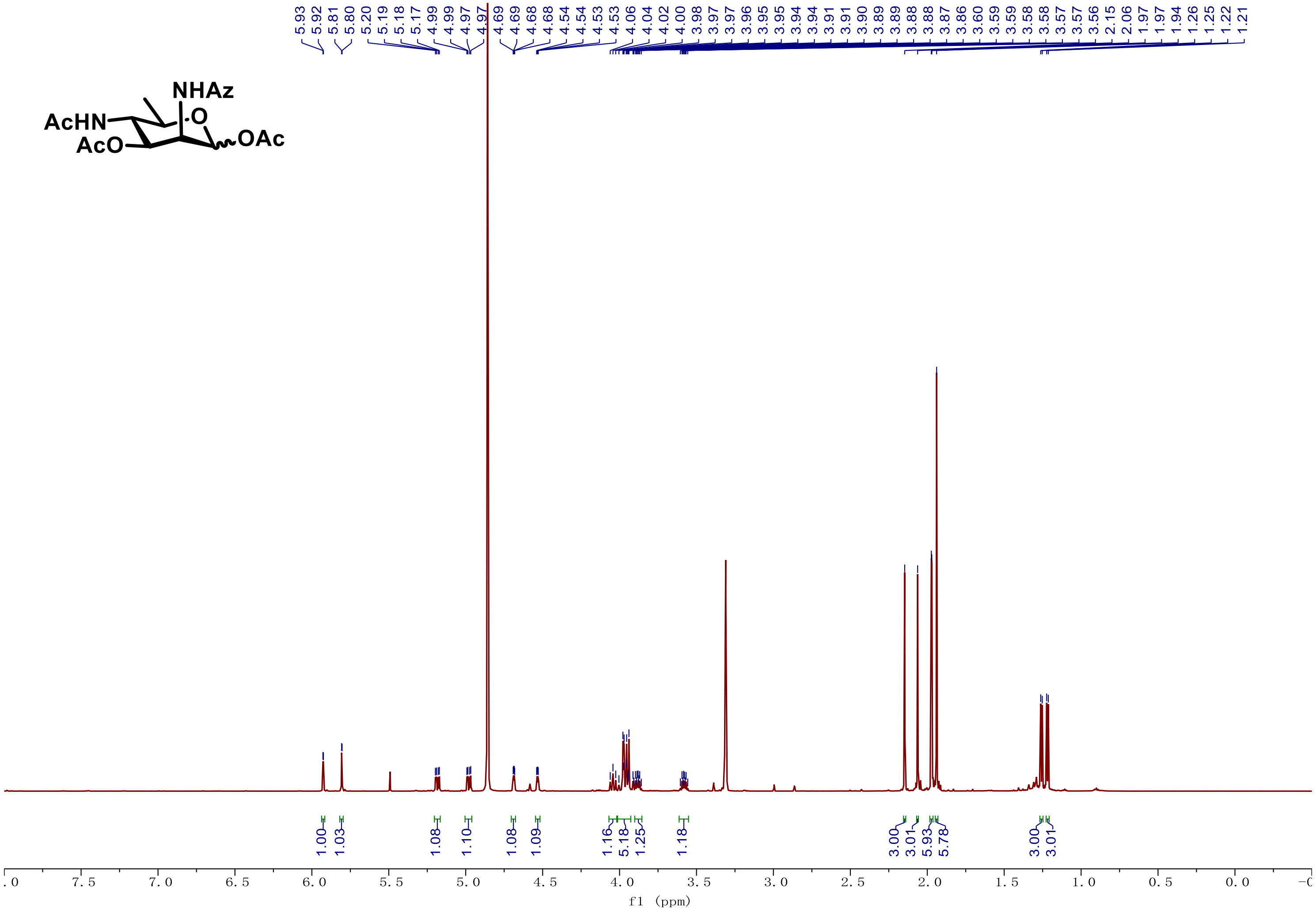

^{13}C NMR spectrum of 3 (151 MHz, CD_3OD)

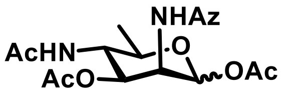

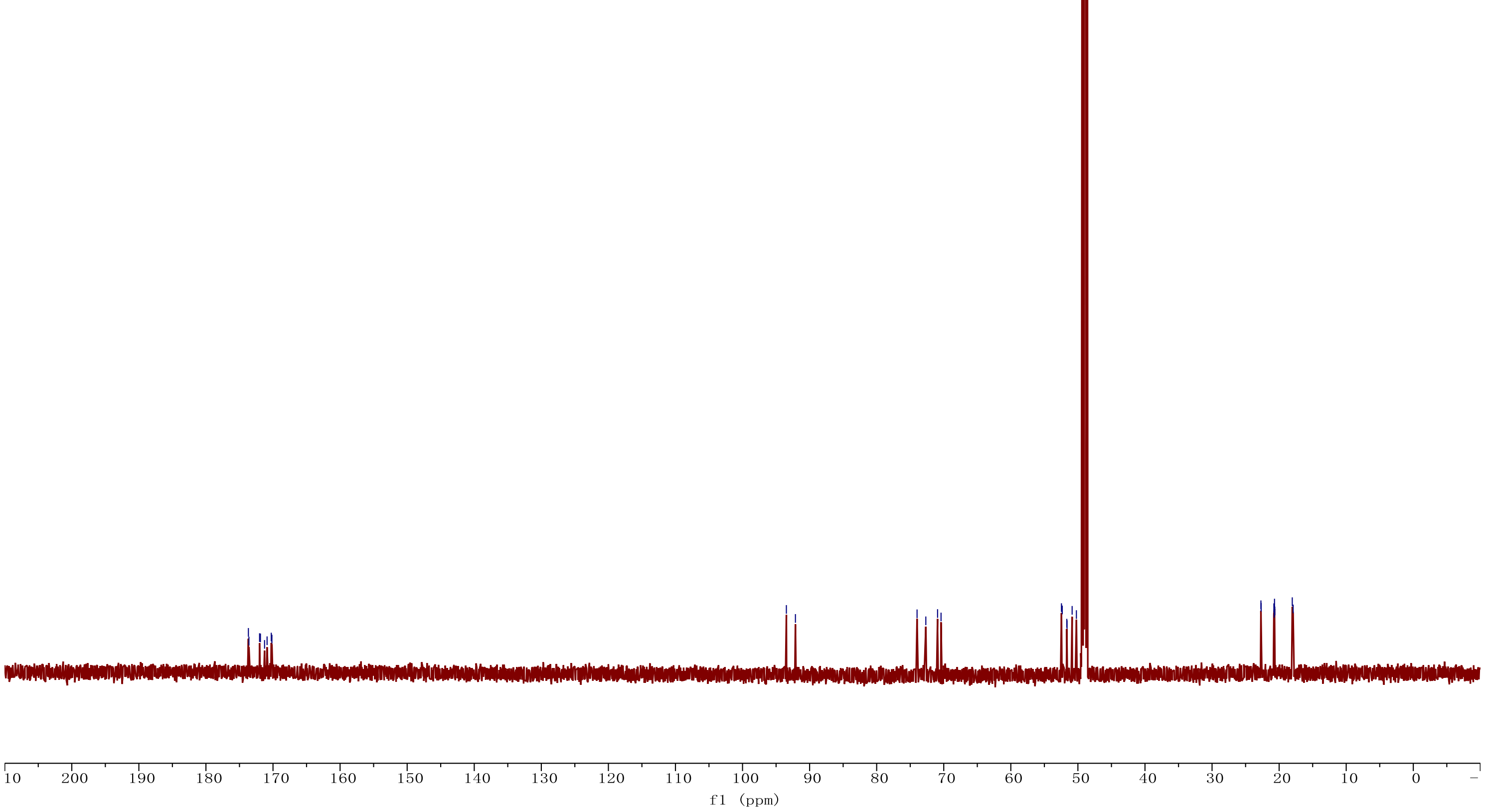

HSQC NMR spectrum of 3 (CD_3OD)

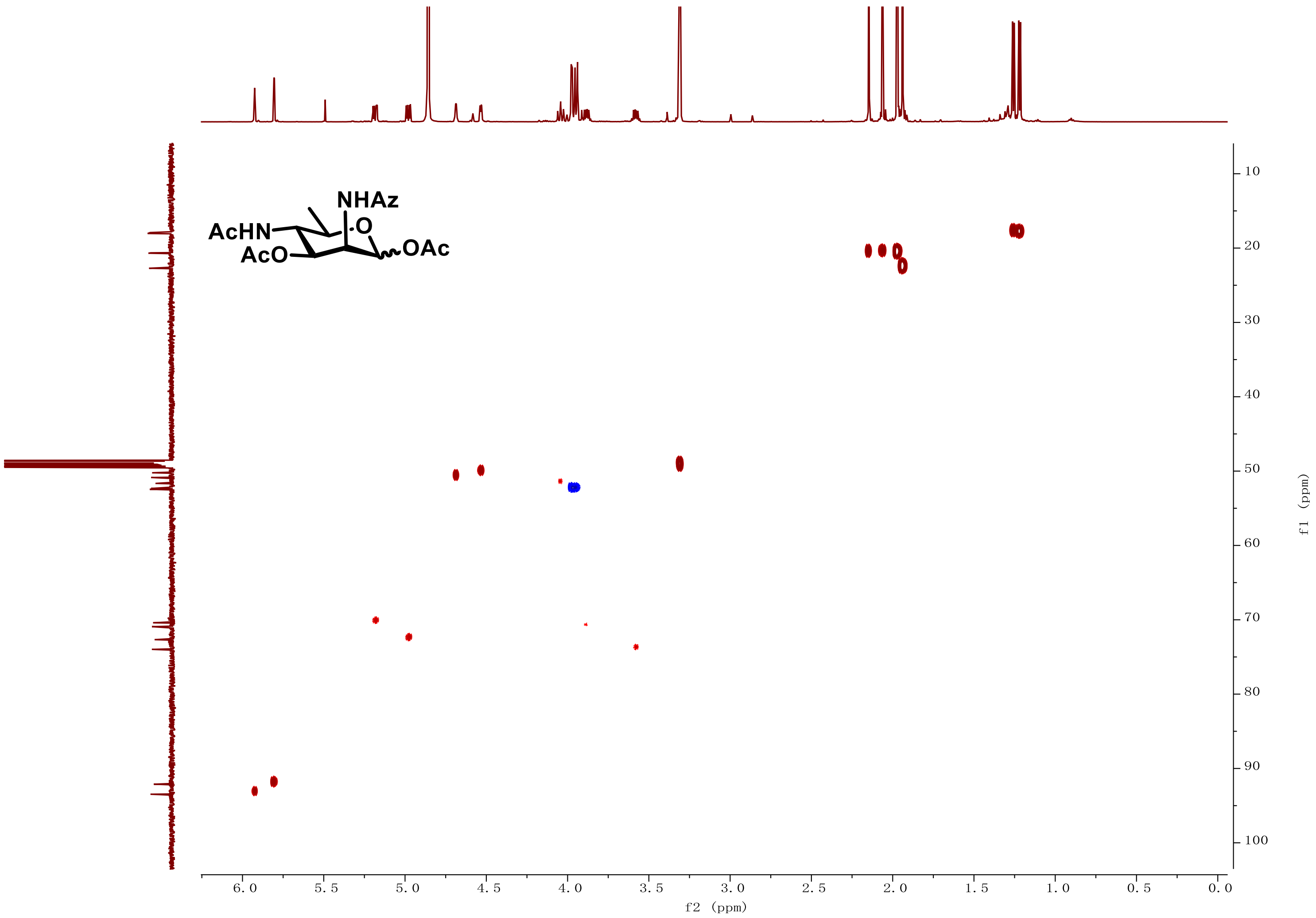

^{1}H NMR spectrum of S2 (600 MHz, CD_3OD)

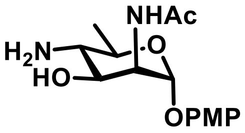

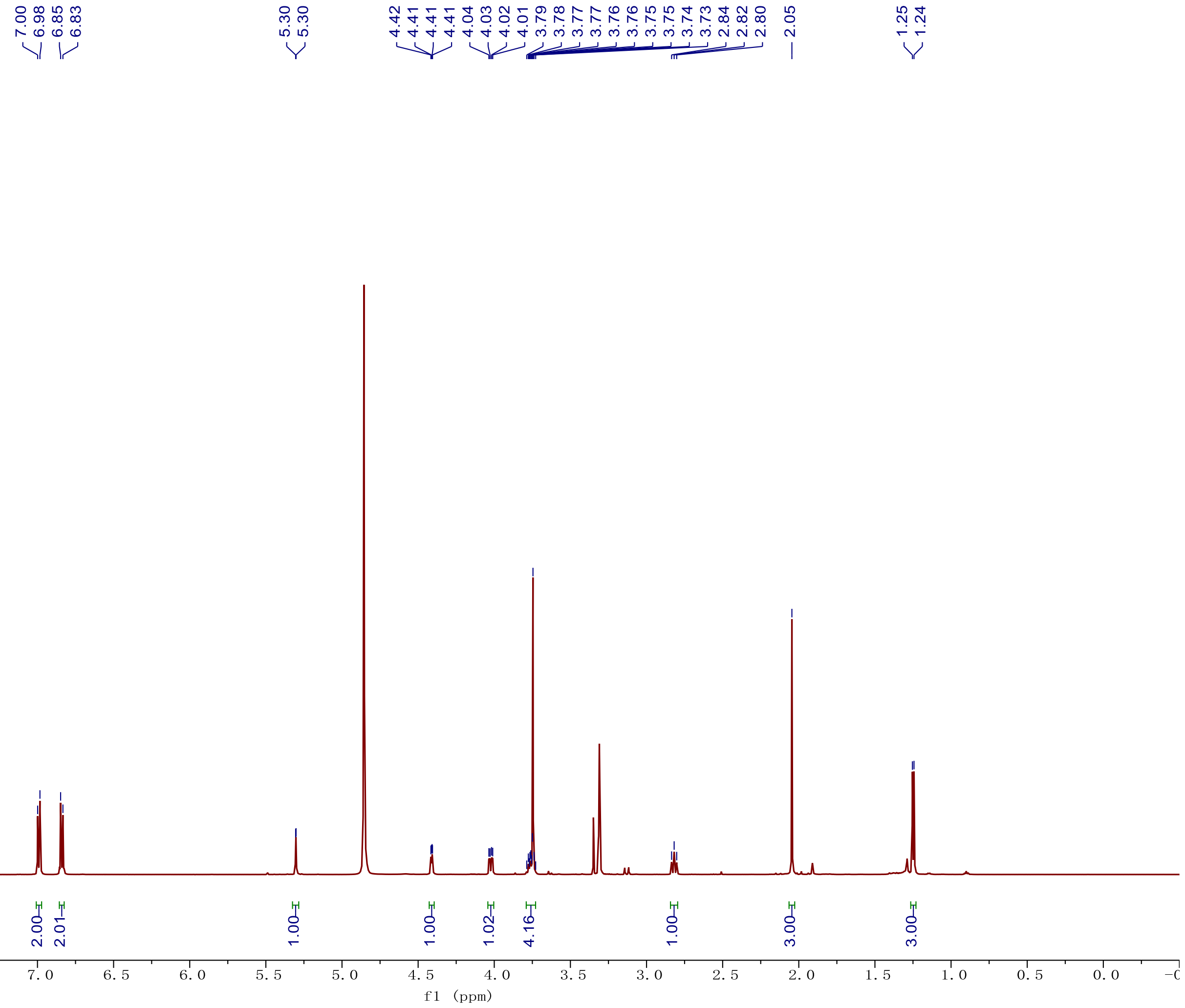

^{13}C NMR spectrum of S2 (151 MHz, CD_3OD)

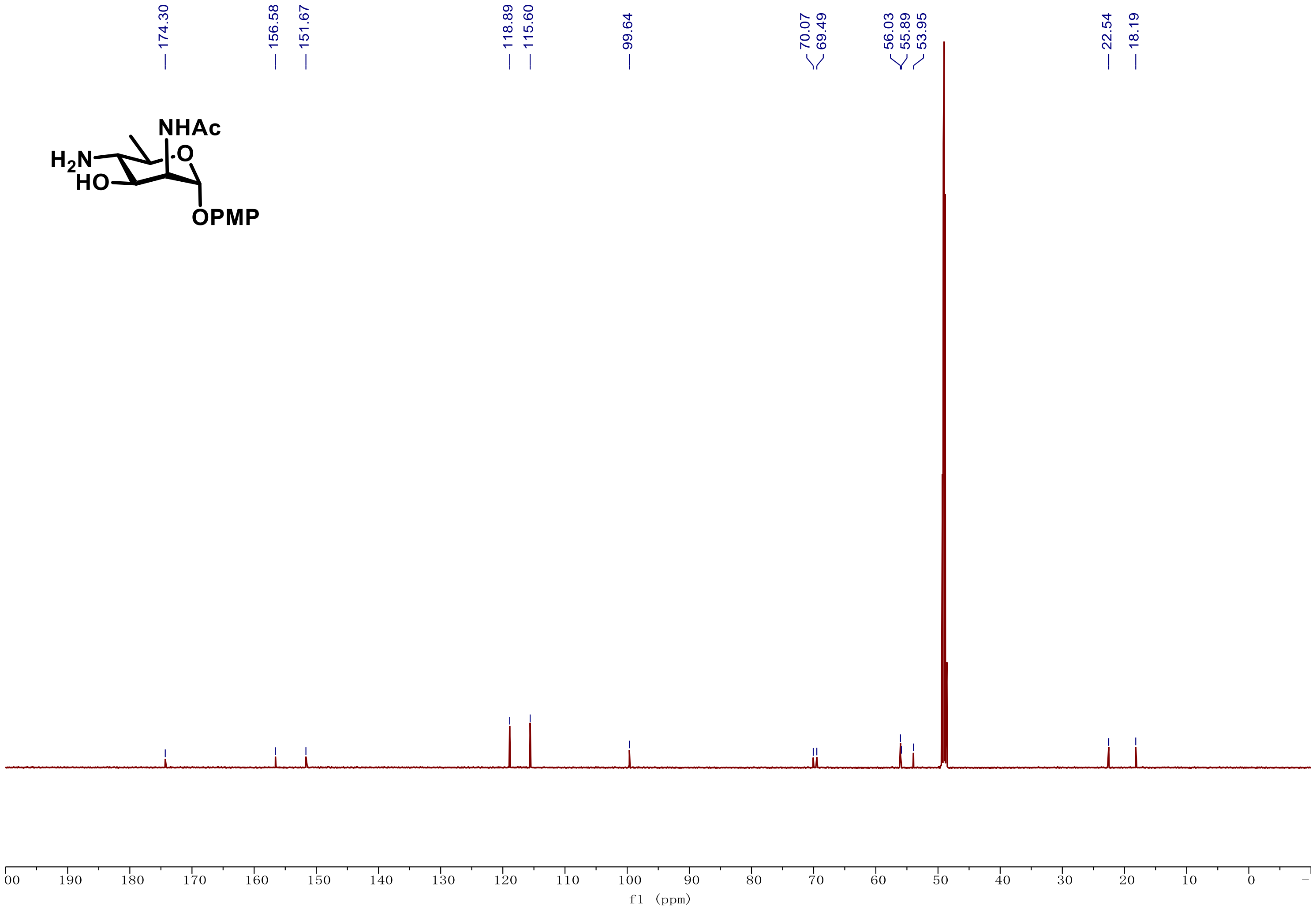

^{1}H NMR spectrum of 18 (600 MHz, CD_3OD)

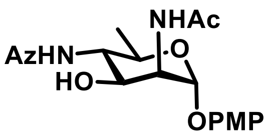

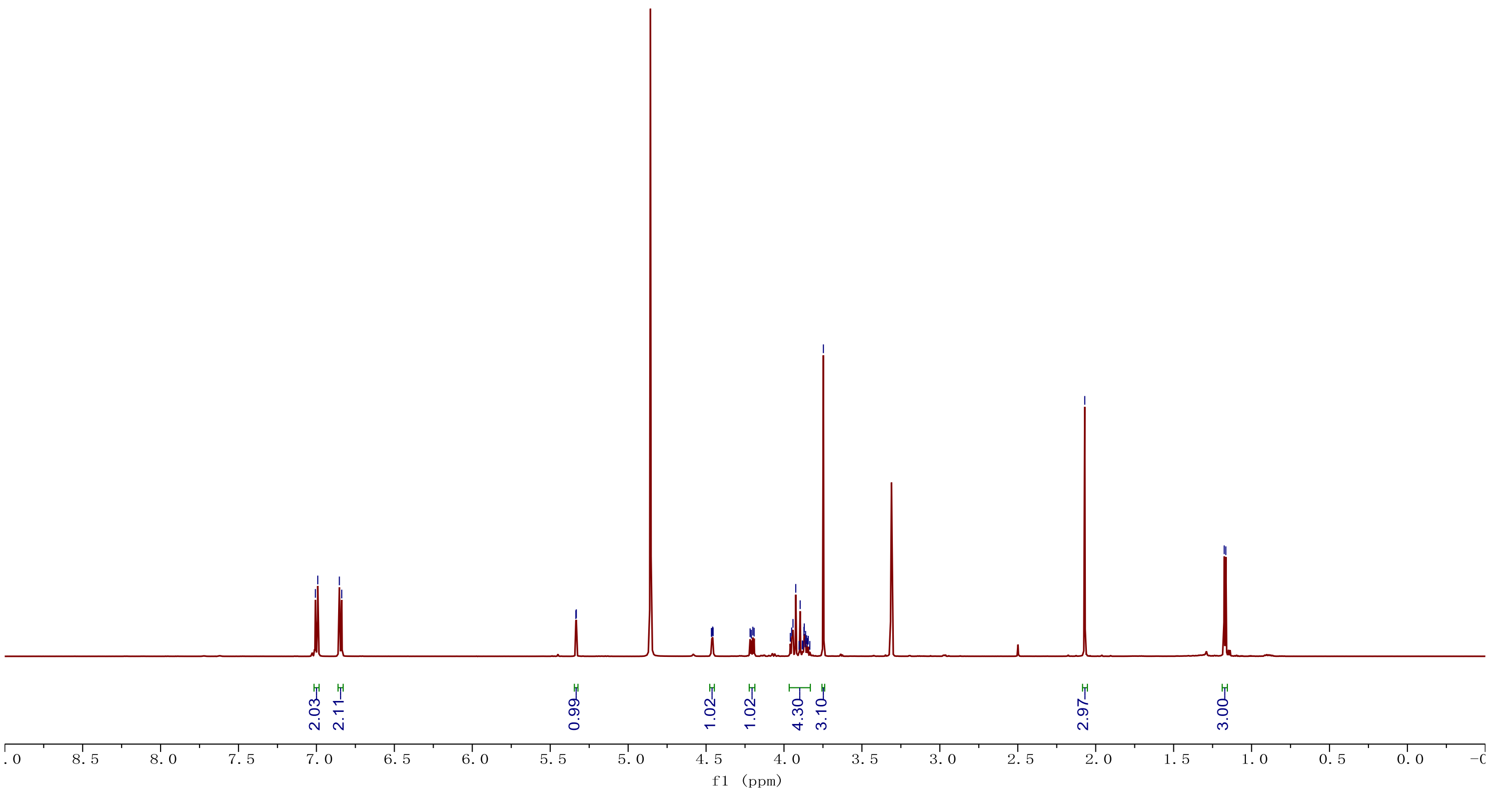

^{13}C NMR spectrum of 18 (151 MHz, CD3OD)

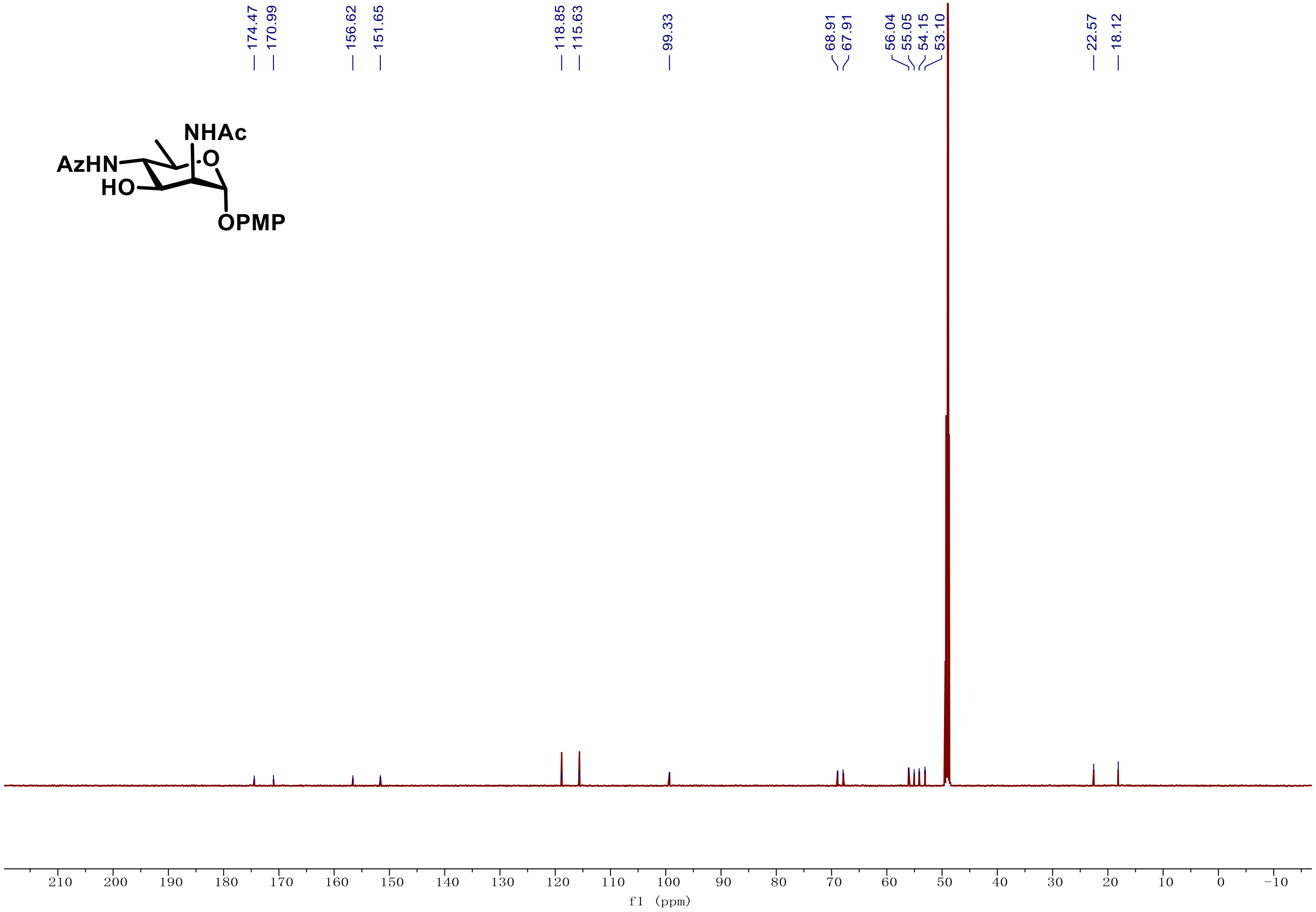

HSQC NMR spectrum of 18 (CD_3OD)

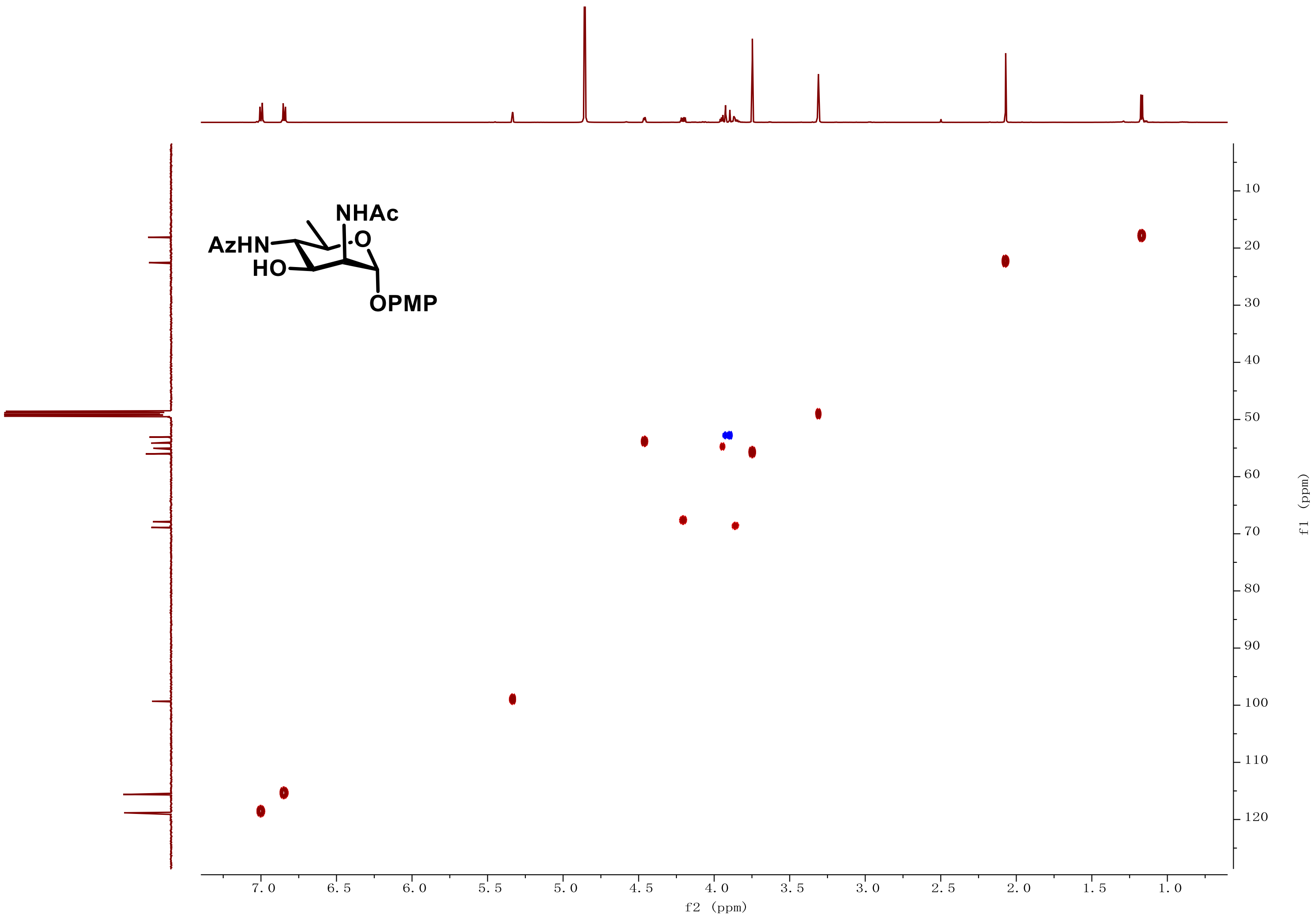

^{1}H NMR spectrum of 4 (600 MHz, CD_3OD)

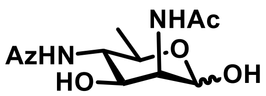

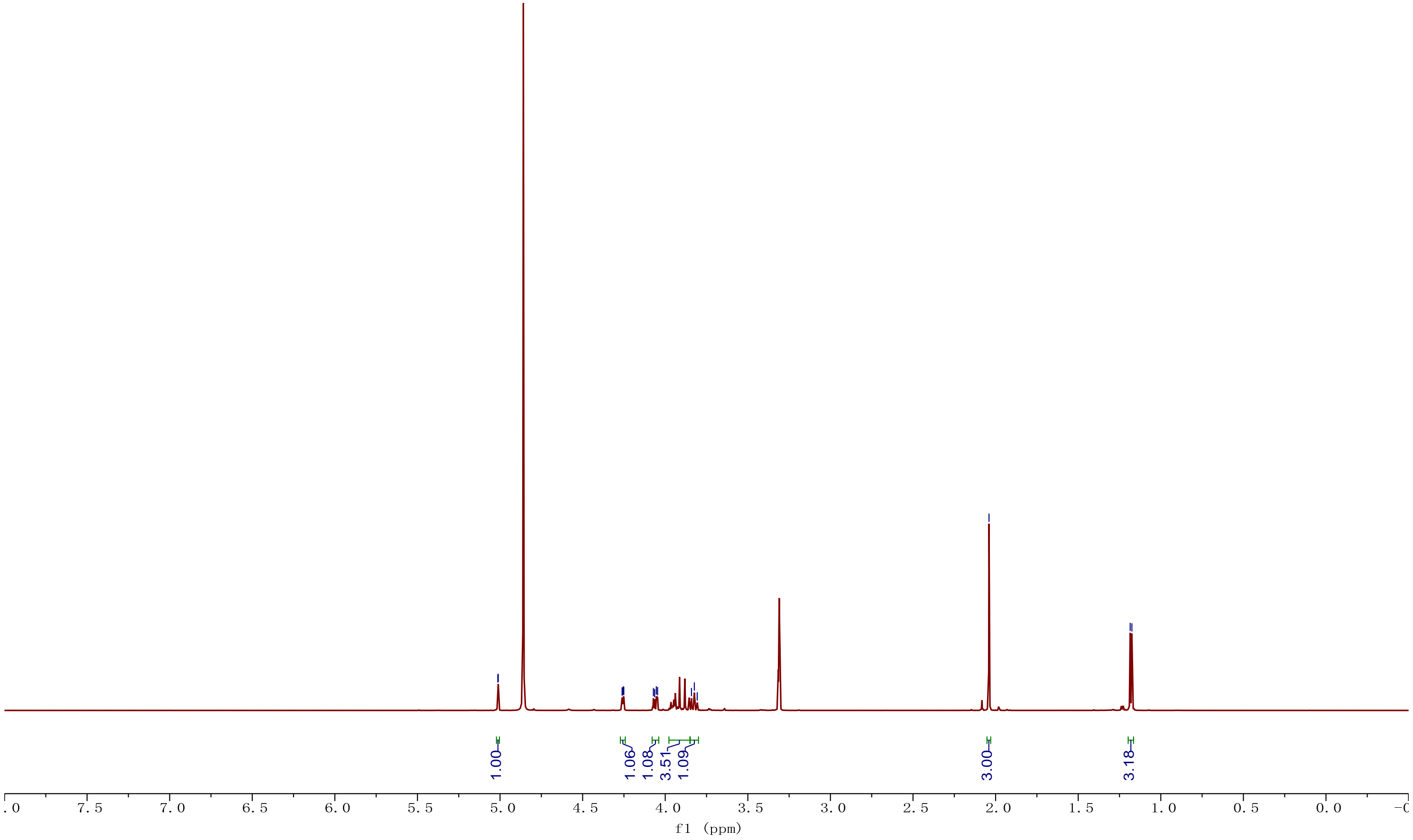

^{13}C NMR spectrum of 4 (151 MHz, CD_3OD)

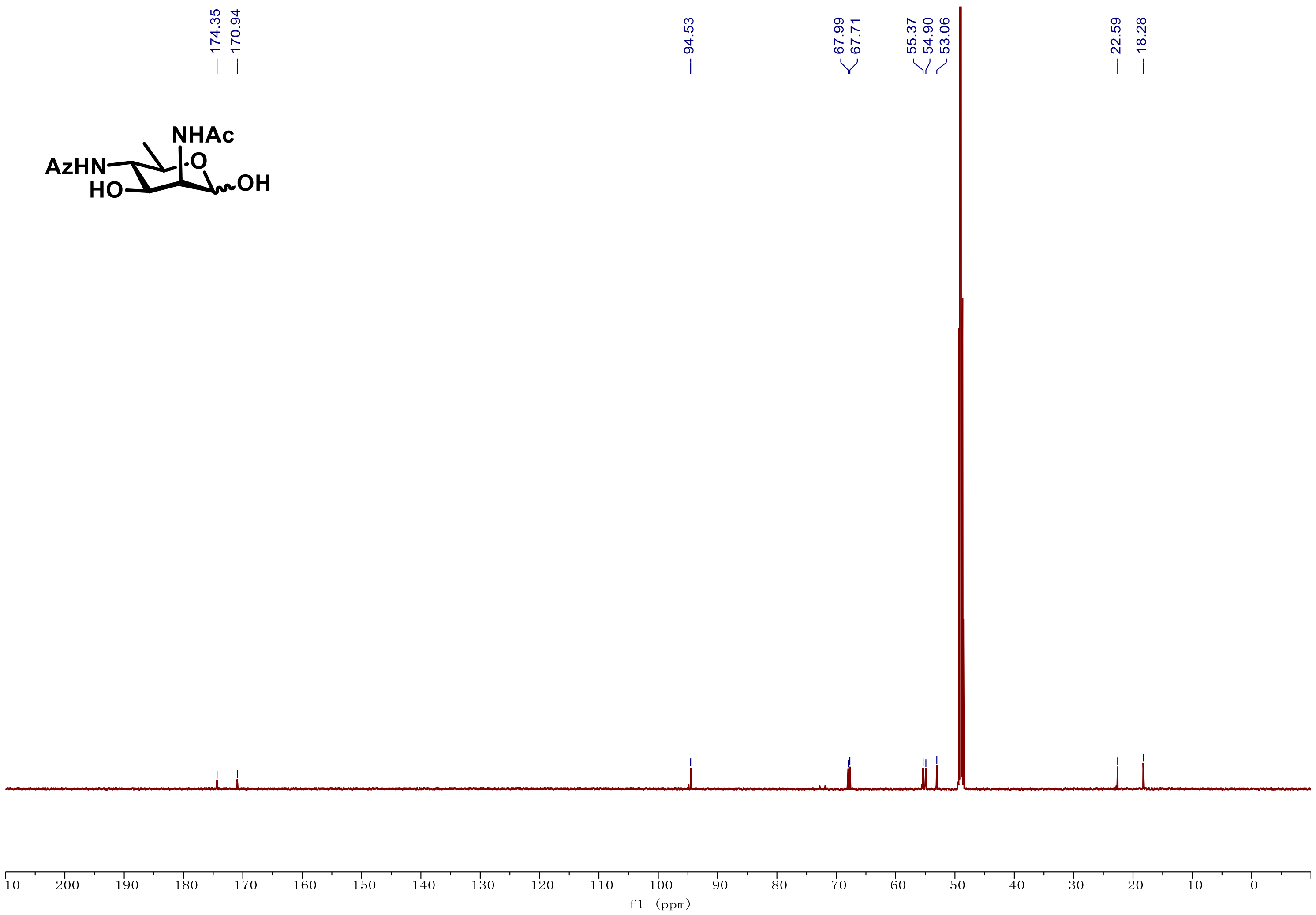

^{1}H NMR spectrum of 5 (600 MHz, CD_3OD)

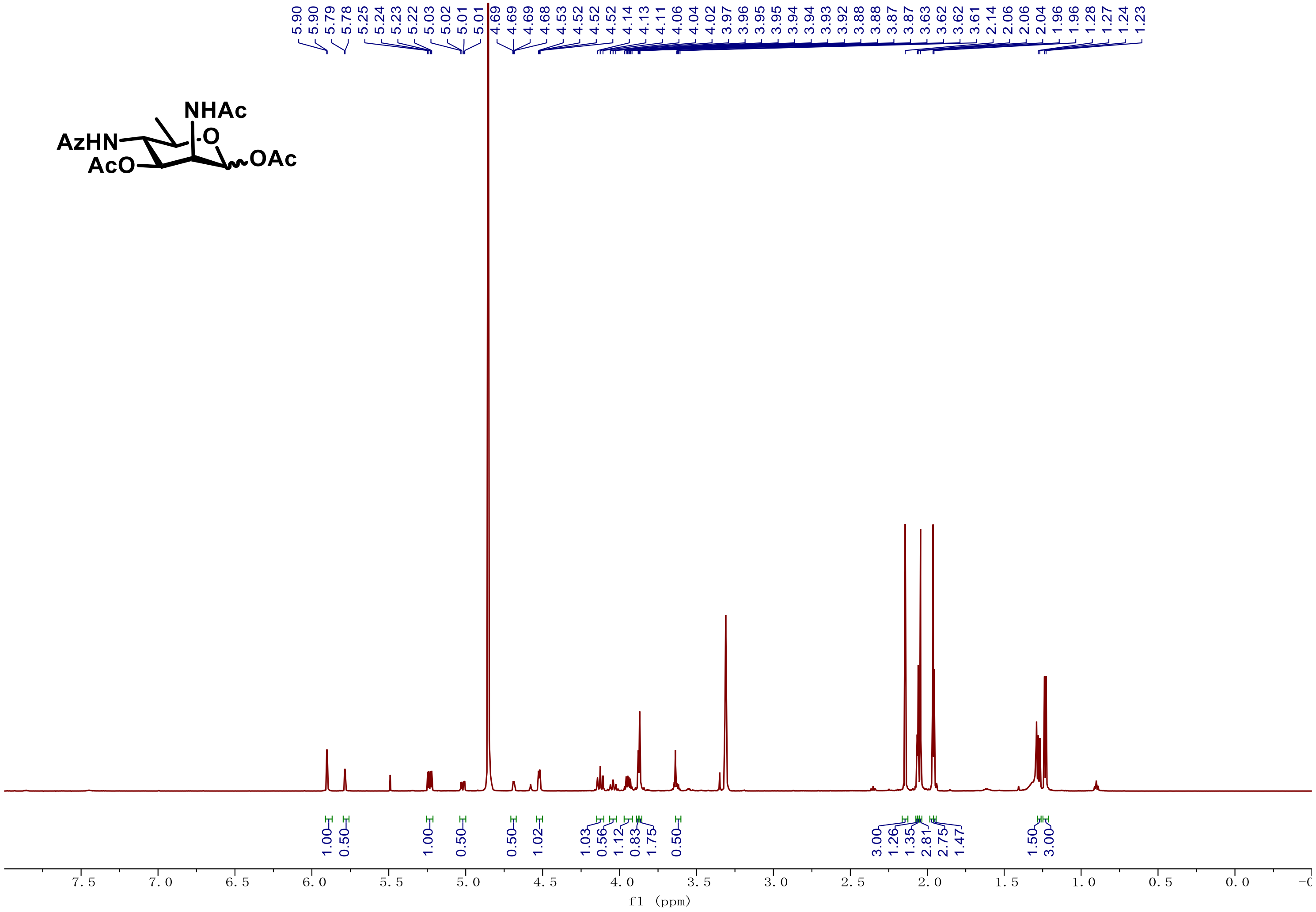

^{13}C NMR spectrum of 5 (151 MHz, CD$_3$OD)

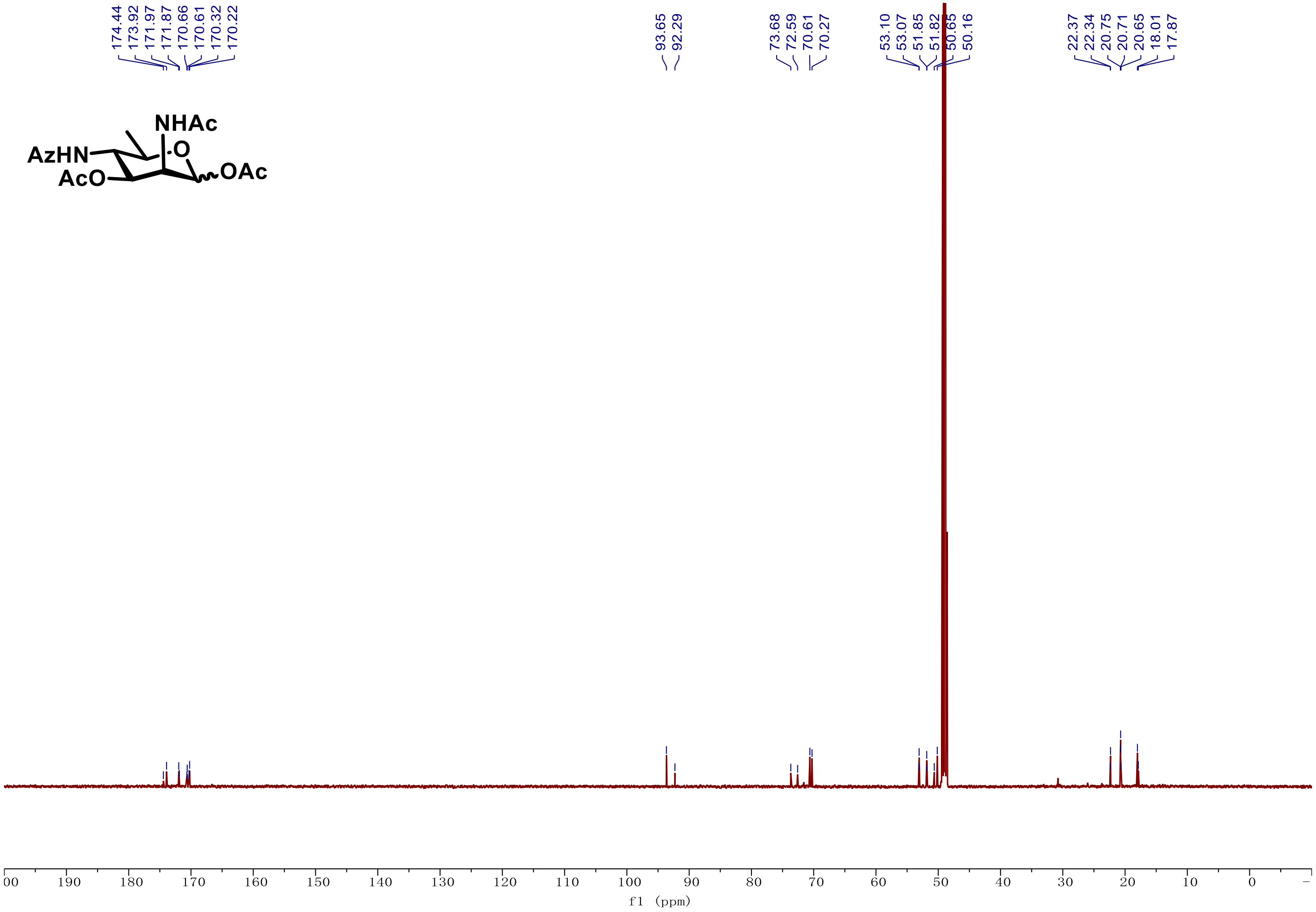

^{1}H NMR spectrum of 19 (600 MHz, CD_3OD)

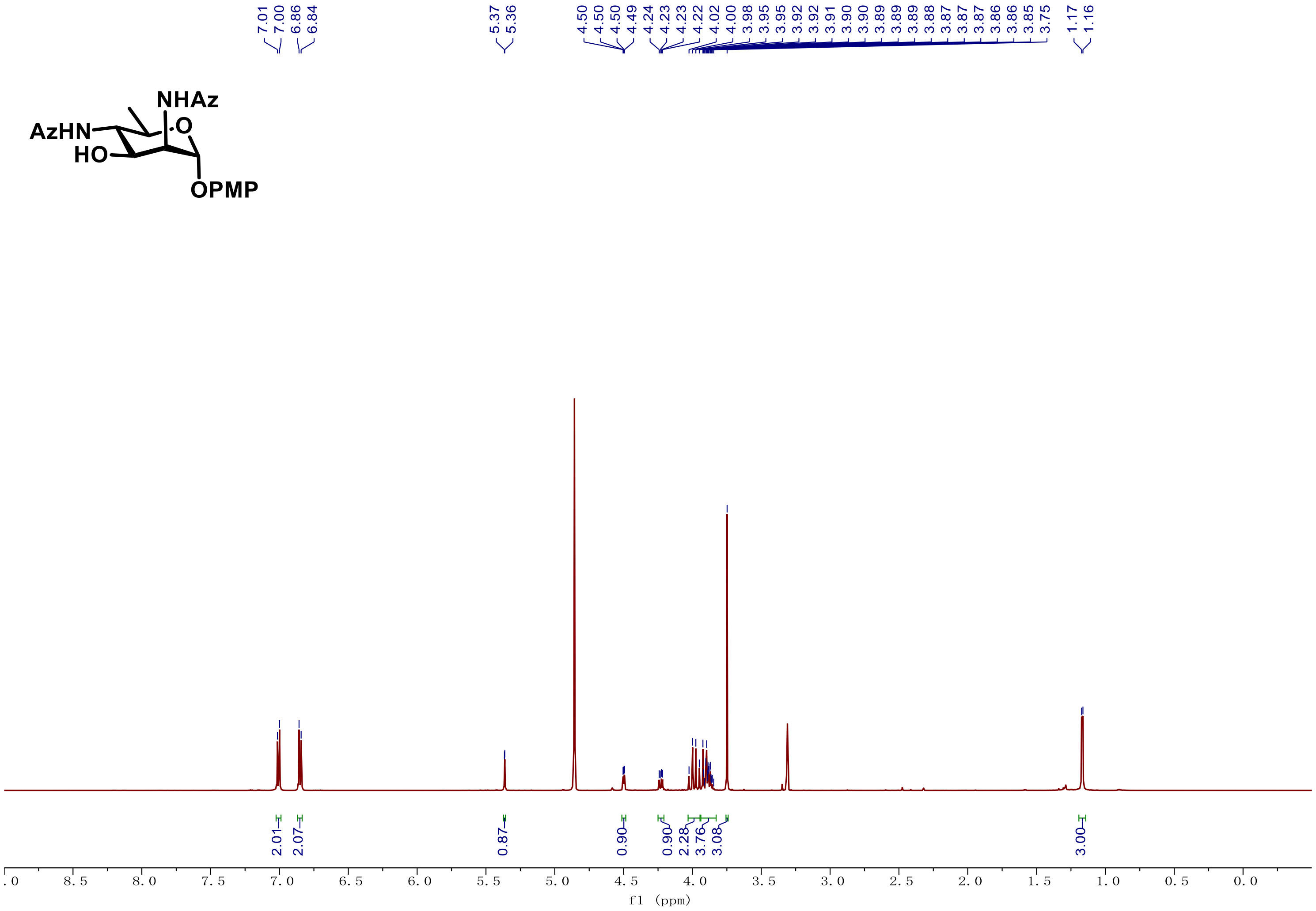

^{13}C NMR spectrum of 19 (151 MHz, CD_3OD)

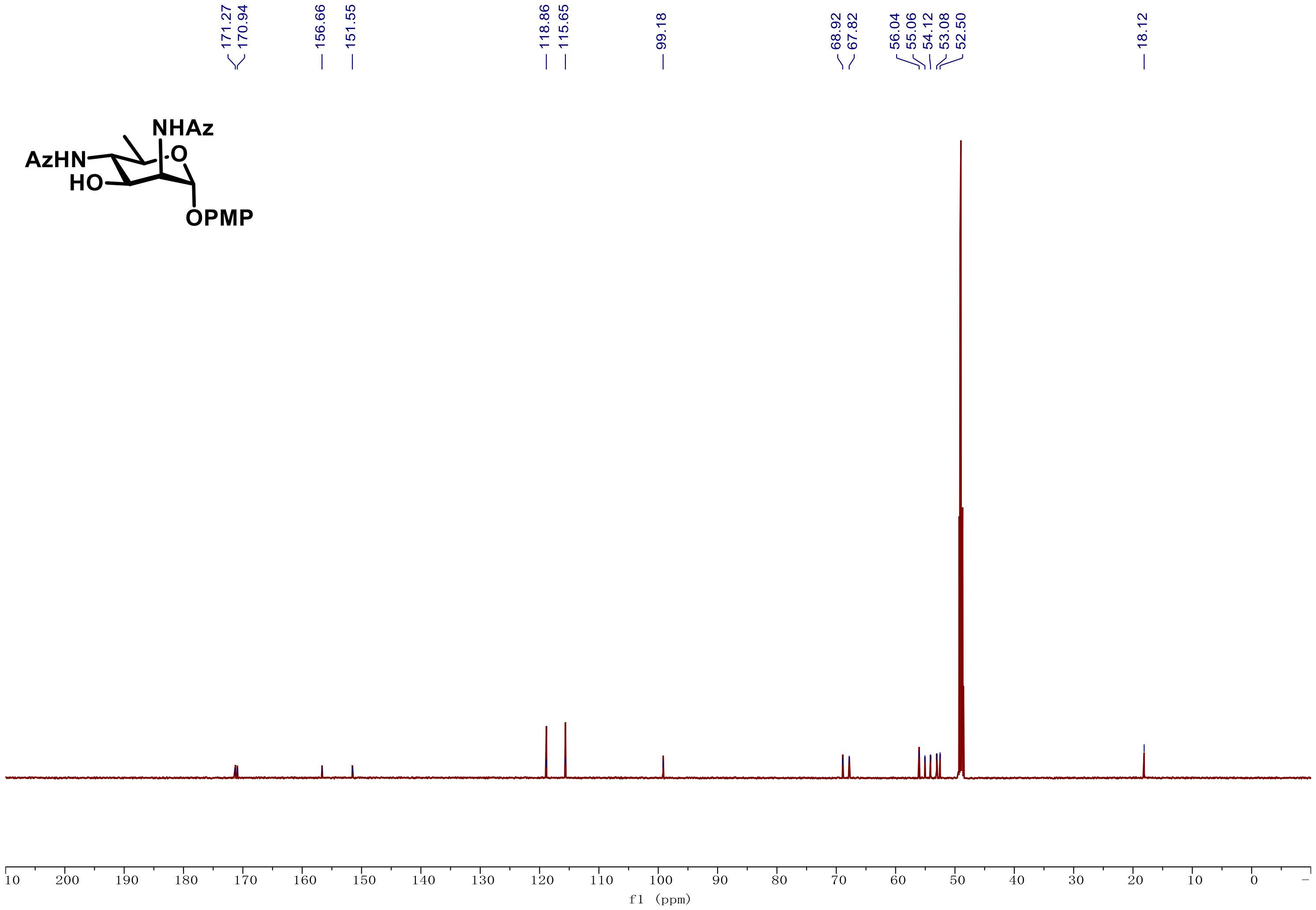

^{1}H NMR spectrum of 6 (600 MHz, CD_3OD)

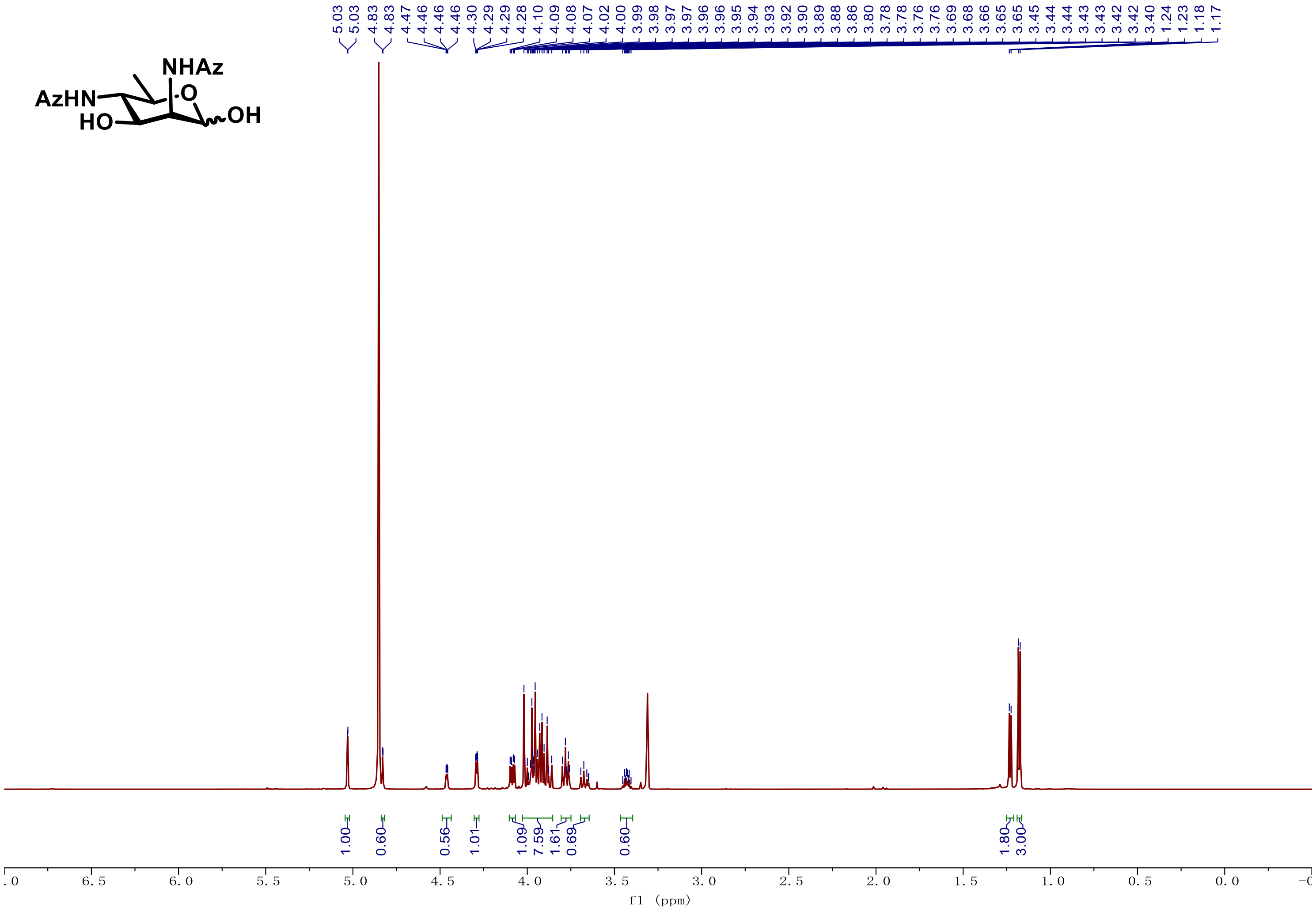

^{13}C NMR spectrum of 6 (151 MHz, CD_3OD)

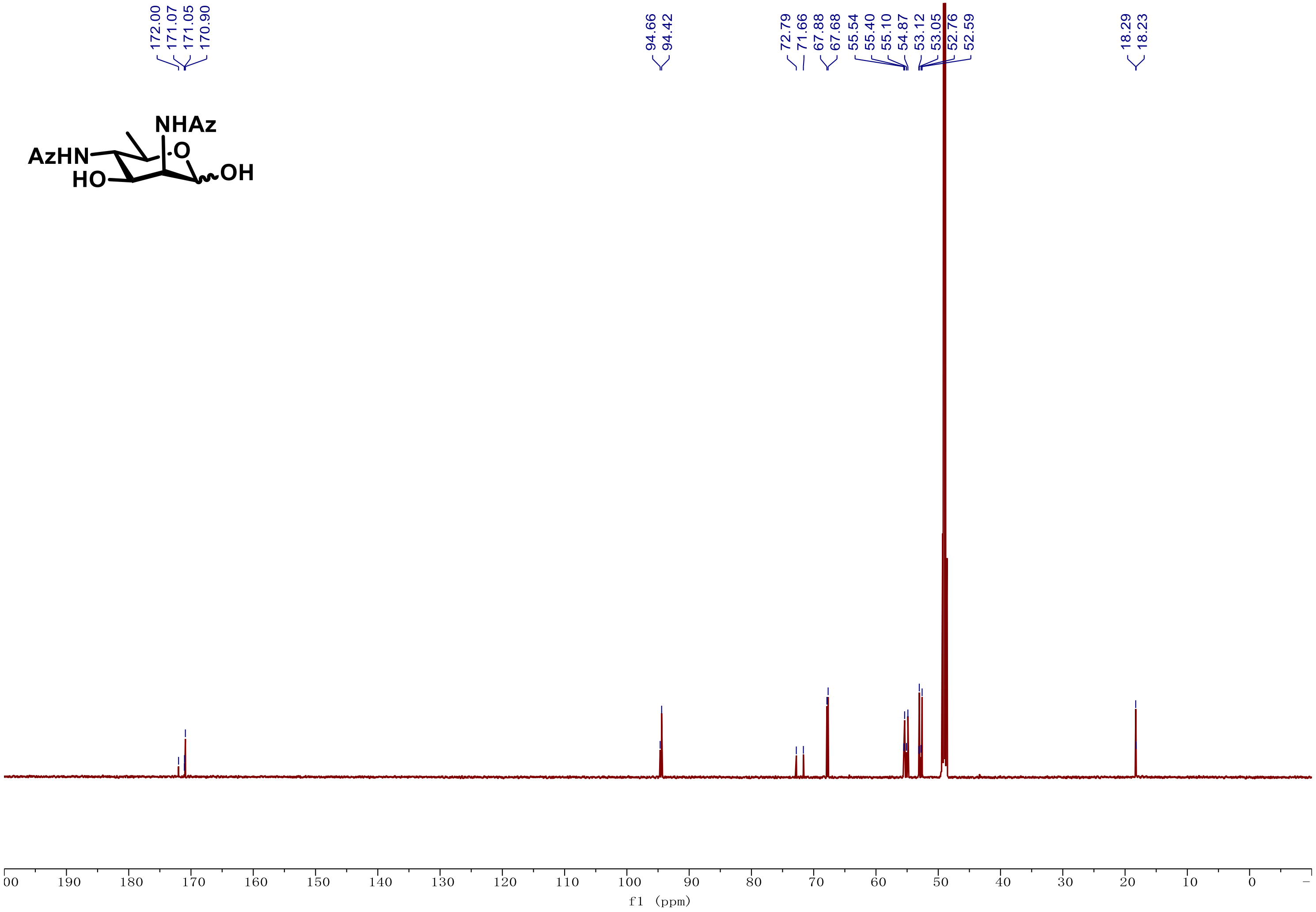

HSQC NMR spectrum of 6 (CD_3OD)

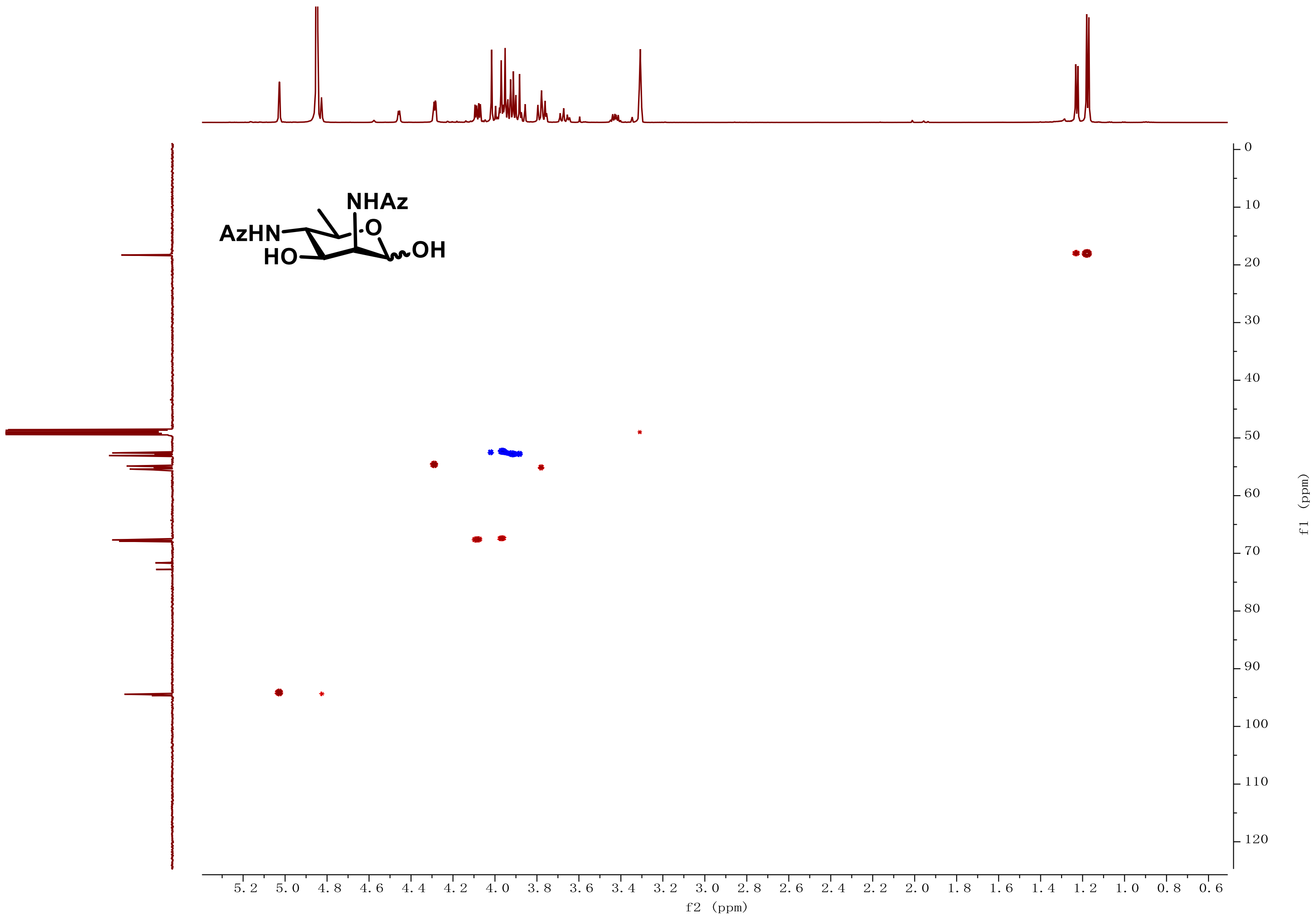

^{1}H NMR spectrum of 7 (600 MHz, CD$_3$OD)

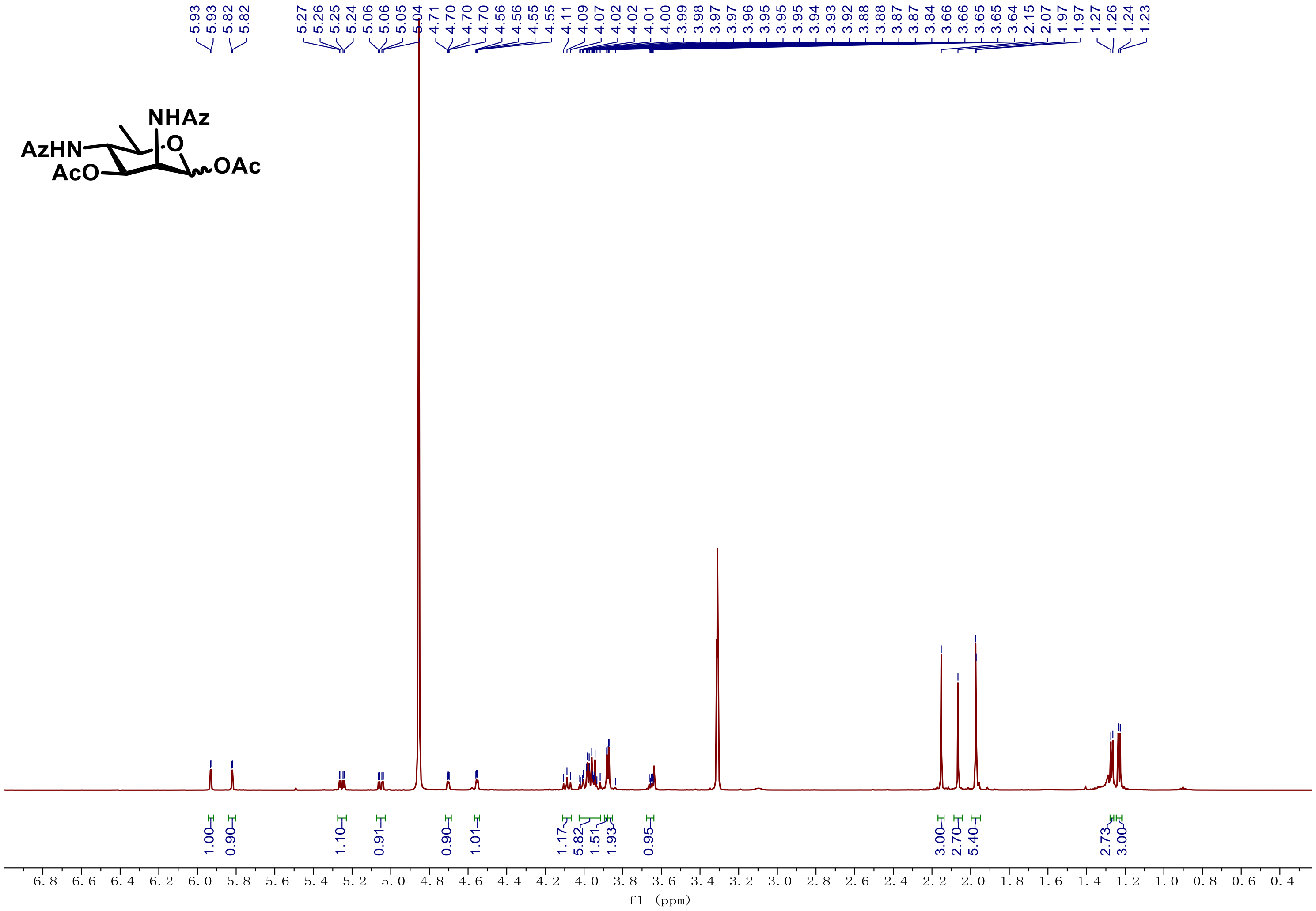

^{13}C NMR spectrum of 7 (151 MHz, CD_3OD)

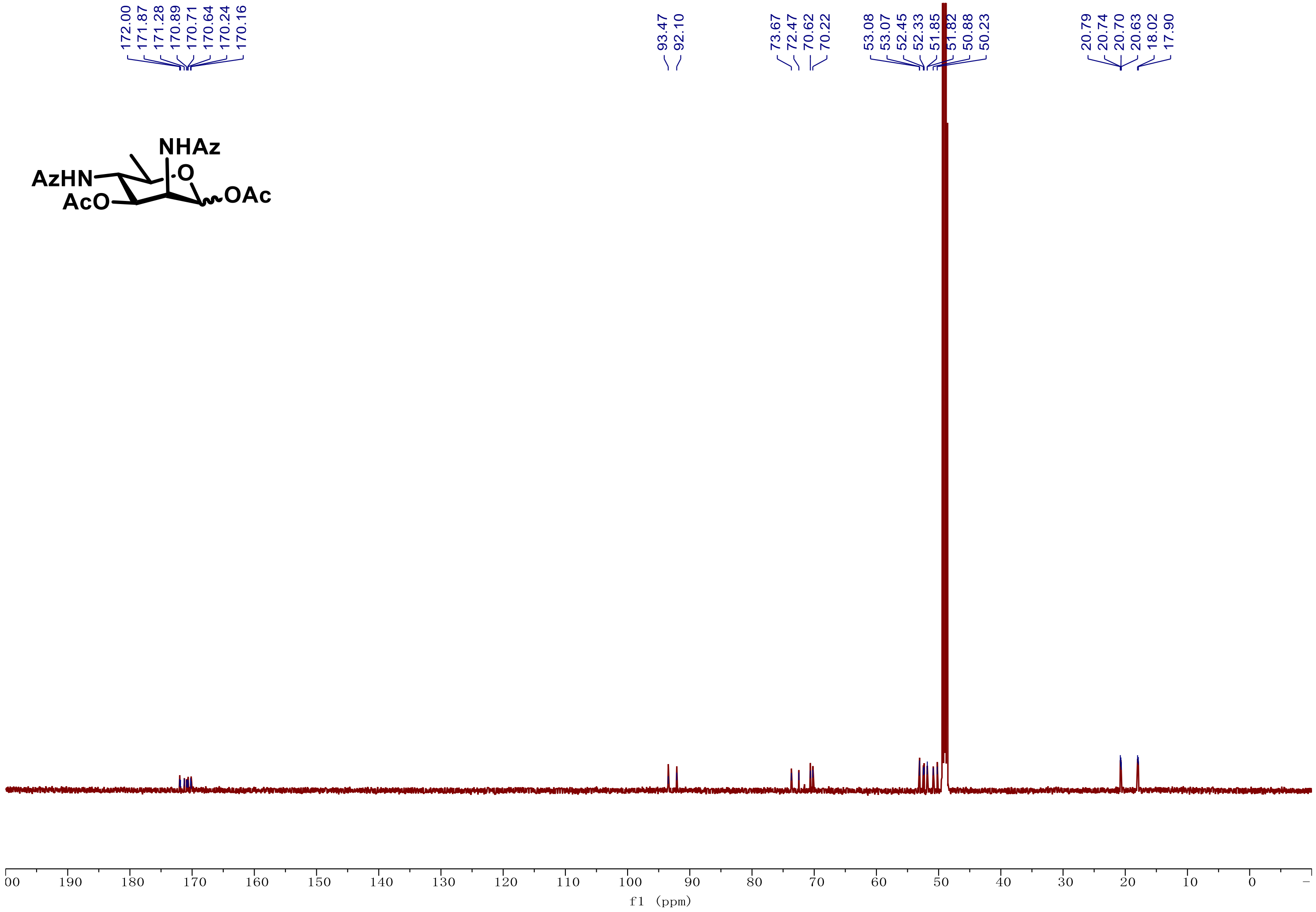

Supplement: Supplementary file 1 — Supporting Information [file ANIE-60-24811-s002.pdf]
